# Boosted Heterogeneous Catalysis by Surface‐Accumulated Excess Electrons of Non‐Oxidized Bare Copper Nanoparticles on Electride Support

**DOI:** 10.1002/advs.202204248

**Published:** 2022-11-17

**Authors:** Sung Su Han, Athira Thacharon, Jun Kim, Kyungwha Chung, Xinghui Liu, Woo‐Sung Jang, Albina Jetybayeva, Seungbum Hong, Kyu Hyoung Lee, Young‐Min Kim, Eun Jin Cho, Sung Wng Kim

**Affiliations:** ^1^ Department of Chemistry Chung‐Ang University Seoul 06974 Republic of Korea; ^2^ Department of Energy Science Sungkyunkwan University (SKKU) Suwon 16419 Republic of Korea; ^3^ Department of Materials Science and Engineering KAIST Daejeon 34141 Republic of Korea; ^4^ Department of Materials Science and Engineering Yonsei University Seoul 03722 Republic of Korea

**Keywords:** electrides, heterogeneous catalysts, non‐oxidized bare copper nanoparticles, surface‐accumulated excess electrons

## Abstract

Engineering active sites of metal nanoparticle‐based heterogeneous catalysts is one of the most prerequisite approaches for the efficient production of chemicals, but the limited active sites and undesired oxidation on the metal nanoparticles still remain as key challenges. Here, it is reported that the negatively charged surface of copper nanoparticles on the 2D [Ca_2_N]^+^∙e^−^ electride provides the unrestricted active sites for catalytic selective sulfenylation of indoles and azaindoles with diaryl disulfides. Substantial electron transfer from the electride support to copper nanoparticles via electronic metal–support interactions results in the accumulation of excess electrons at the surface of copper nanoparticles. Moreover, the surface‐accumulated excess electrons prohibit the oxidation of copper nanoparticle, thereby maintaining the metallic surface in a negatively charged state and activating both (aza)indoles and disulfides under mild conditions in the absence of any further additives. This study defines the role of excess electrons on the nanoparticle‐based heterogeneous catalyst that can be rationalized in versatile systems.

## Graphical abstract

A combination of copper nanoparticle and exotic electride support provides novel heterogenous catalysts for selective sulfenylation of (aza)indoles with diaryl disulfides under mild conditions in the absence of any additives. Surface‐accumulated excess electrons of the negatively charged copper nanoparticles anchored on dicalcium nitride, [Ca_2_N]^+^∙e^−^ electride are responsible for the catalytic reaction that has not been accomplished with typical copper nanoparticles.

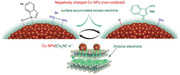

## 1 Introduction

Heterogeneous catalysis plays a critical role in the chemical and energy industries with the undisputable advantages such as the sustainability and facile product separation.^[^ ^1^ ^−^ ^3^ ^]^ Among the state‐of‐the‐art heterogeneous catalyst systems, metal nanoparticles (NPs) anchored on a support have been of great interest, demonstrating an efficient heterogeneous reaction for catalyzed organic compounds such as ammonia and sulfuric acid in an industrial scale process.^[^ ^4^, ^5^ ^]^ It has been well established that the electronic and geometric structures of metal NPs are the key component to design a catalyst and control its catalytic performance, thus manipulating the size, shape, and composition of metal NPs is the primary approach for developing an efficient catalyst.^[^ ^6^, ^7^ ^]^ Furthermore, a profound impact on the catalytic behavior of metal NPs is made upon the metal–support interaction (MSI), which includes the phenomena such as charge transfer and the interfacial perimeter.^[^ ^6^ ^]^ Indeed, the redistribution of electrons within both NPs and supports can induce the change in oxidation states of metal atoms of NPs and metal ions of supports. However, this phenomenon is typically limited to a few atomic layers of the interfacial region.^[^ ^8^, ^9^ ^]^ Considering that the difference in work function (*ϕ*) values between the metal NP and the support determines the direction of the charge transfer and its magnitude,^[^ ^10^ ^]^ an appropriate selection of a support material with extremely low *ϕ* can further drive the charge transfer to metal NPs, ultimately activating the metal atoms to have an intriguing electronic state and thus realizing numerous active sites over the whole surface area of NPs.

A strong electronic interaction between an electroactive support material with high‐density mobile electrons and metal NP enables a substantial charge transfer, leading to the negatively charged surface state of metal NPs according to the Gauss's law.^[^ ^11^ ^−^ ^14^ ^]^ When excess electrons are transferred to the metal NPs, which is a good conductor, excess electrons are not bound to a particular metal ion but rather free to move around. These free electrons will be rearranged and accumulated near the metal atoms at the surface of NPs. Indeed, this phenomenon can be found in metal clusters such as Na and Ag clusters.^[^ ^15^ ^]^ More critically, in the metal NPs with surface‐accumulated excess electrons, the intriguing negatively charged surface state can offer numerous sites for reactant activation to generate the intermediates in the targeted chemical reaction; therefore, facilitating the massive charge transfer from a support material to metal NP is a straightforward approach for an efficient metal NPs‐based heterogeneous catalyst. Recently, it is found that the bare copper NPs (Cu NPs) grown on the [Gd_2_C]^2+^·2e^−^ electride with the *ϕ* of ≈2.8 eV show the negatively charged surface state due to the surface‐accumulated excess electrons and the ultrastrong oxidation resistance in air, keeping the metallic surface state during a long period over several months.^[^ ^16^ ^]^ Thus, in order to prove the potential of the electride as a support material and demonstrate the metal NPs with numerous active sites on excess electrons accumulated surface, we design the bare Cu NPs supported on [Ca_2_N]^+^·e^−^ electride (Cu NPs/[Ca_2_N]^+^·e^−^) for an efficient heterogeneous catalyst.

It is noted that the [Ca_2_N]^+^·e^−^ electride has a low *ϕ* value of ≈2.6–3.5 eV depending on the surface orientation.^[^ ^17^ ^]^ Moreover, benefited from the fully delocalized nature of anionic electrons at interlayer space, the electronic mobility of ≈160 cm^2^ V^−1^ s at 300 K was obtained, while the [Gd_2_C]^2+^·2e^−^ electride with strongly localized anionic electrons showed the value of ≈20 cm^2^ V^−1^ s at 300 K.^[^ ^17^, ^18^ ^]^ Owing to the low *ϕ* originating from the high electron density with ≈2.3 × 10^22^ cm^−3^ and delocalized nature of anionic electrons, the 2D [Ca_2_N]^+^∙e^−^ electride spontaneously transfers its highly mobile electrons to the Cu NPs (≈4.5 eV). In contrast to the low selectivity of conventional Cu‐based heterogeneous catalysts due to various Cu oxidation states originated from the formation of Cu oxides,^[^ ^19^ ^−^ ^21^ ^]^ the Cu NPs on the [Ca_2_N]^+^·e^−^ electride can improve the oxidation state‐ and site‐dependent catalytic activity because the system retains the metallic surface with the transferred excess electrons that can provide numerous active sites regardless of morphology and facet. Here, we show an efficient catalytic sulfenylation of indole compounds as a model substitution reaction utilizing Cu NPs/[Ca_2_N]^+^·e^−^ catalyst. Among the indole derivatives, 3‐sulfenylindoles are currently attracting considerable attention due to their therapeutic value in the treatment of HIV, cancer, obesity, heart disease, and allergies.^[^ ^22^ ^−^ ^24^ ^]^ While several strategies have been explored for the 3‐sulfenylation of indoles with thiols, disulfides, sulfenyl halides, *N*‐thioimides, sulfonium salts, quinone mono‐*O*,*S*‐acetals, and arylsulfonyl chlorides, the required excess sulfenylating agents or additives, limited substrate scope, and undesired byproducts have been unsettled obstacles.^[^ ^25^ ^−^ ^40^ ^]^ The present sulfenylation reaction using Cu NPs/[Ca_2_N]^+^·e^−^ catalyst is successfully accomplished with various functional groups on both reactants, with a broad substrate scope. The performed high‐yield regioselective synthesis of 3‐sulfenylindole compounds is ascribed to the surface‐accumulated excess electron on the Cu NPs, suggesting that the catalytic activity of Cu NPs can be effectively enhanced by inducing a negatively charged surface state through a strong electronic MSI.

## 2 Results and Discussions

### 2.1 Charge Transfer for Strong Metal–Support Interactions

**Figure 1:**
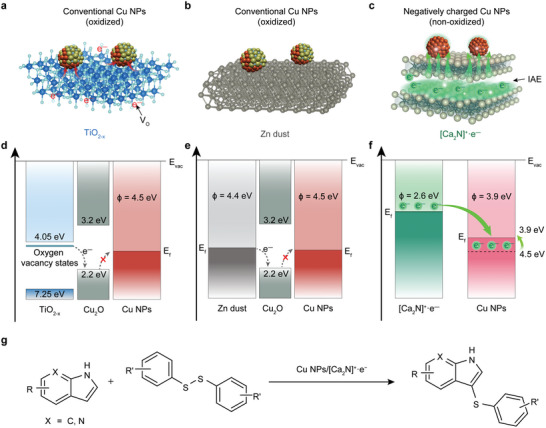
Schematic illustrations of heterogeneous catalyst systems of Cu NPs on different supports. a−c) Cu NPs on TiO_2−_ *_x_* a), Zn dust b), and [Ca_2_N]^+^·e^−^ electride c). Oxygen vacancies (*V*_o_) in the reduced titanium dioxide provide additional electrons to Cu NPs (red arrows in a). The inherent anionic interstitial electrons are transferred to Cu NPs (green arrows in c). d−f) Comparison of the energy levels for electrons and their flow to Cu NPs. Oxidized Cu NPs on TiO_2−_ *_x_* a) and Zn dust b) act as a barrier for the charge transfer from supports d,e). Green arrows in f) represent the efficient charge transfer of anionic interstitial electrons due to the non‐oxidized Cu NPs and the decreased work function of Cu NPs on the [Ca_2_N]^+^·e^−^ electride support. g) Regioselective sulfenylation of (aza)indoles with disulfide catalyzed by Cu NPs/[Ca_2_N]^+^·e^−^.

**Figure** **1** highlights the advantage of the electride support with a low *ϕ* for enhancing charge transfer to the Cu NPs by comparing the energy diagrams with typical heterogeneous catalysts. The zinc dust and reduced titania (Figure 1a,b), which are conventional supports for NPs, have two major difficulties in transferring the electrons to the Cu NPs; 1) a scant difference in *ϕ* value and 2) natural formation of copper oxide (Figure 1d,e). These phenomena restrict the formation of active sites for catalytic reactions to the interfacial region. On the contrary, the large difference in *ϕ* values between Cu NPs and [Ca_2_N]^+^·e^−^ induces a strong interfacial electric potential, triggering the charge transfer to Cu NPs (Figure 1c,f). The transferred electrons are excessive for the Cu NPs and expelled to the surface region, resulting in the negatively charged surface state. Consequently, the surface‐accumulated excess electrons lead to the decrease in the *ϕ* value of Cu NPs and acts as a barrier for the oxidation in air. This electron‐rich Cu NPs/[Ca_2_N]^+^·e^−^ system is evaluated for the catalytic activity without any additives in the regioselective sulfenylation of (aza)indoles with disulfides (Figure 1g).

### 2.2 Catalytic Sulfenylation of Indoles

**Table** **1** summarizes the results of catalytic sulfenylation for the Cu NPs/[Ca_2_N]^+^·e^−^ as well as other systems of Cu NPs and possible Cu reagents (1.0 equiv. of reagent represents 0.1 mmol of [Ca_2_N]^+^·e^−^ electride or other support materials containing 0.15 equiv. of Cu) under the optimized solvent and reaction temperature conditions (see Table S1 in the Supporting Information for the details). The reaction was carried out using indole **1a** and diphenyl disulfide **2a** as model substrates (scheme in Table 1). For a relevant evaluation of the catalytic activity, we first compared the catalytic performance of Cu NPs/[Ca_2_N]^+^·e^−^ with commercial Cu NPs (average size ≈32 nm) and [Ca_2_N]^+^·e^−^ electride (entries 1 and 2). It is noticeable that sole Cu NPs and [Ca_2_N]^+^·e^−^ electride showed no reaction, whereas the reaction of Cu NPs/[Ca_2_N]^+^·e^−^ produced the desired 3‐phenylthioindole (**3aa**) in 93% yield (entry 3), indicating that the catalytic activity was verified in the present heterogenous system of the Cu NPs grown on the [Ca_2_N]^+^·e^−^ electride support. Furthermore, we also examined the catalytic performance of the separated Cu NPs from the electride^[^ ^41^ ^]^ (Table S2, Supporting Information) which exhibited no detectable yield of the sulfenylation. Importantly, when the Cu NPs/[Ca_2_N]^+^·e^−^ loading was lowered, the reaction yield was sustained (entries 4 and 5). To validate the efficiency of the electride support in the sulfenylation reaction, alternative electron donating supports, such as sodium ascorbate and Zn dust, were examined with Cu NPs and CuI compound (entries 6–8). These reactions showed no catalytic activity, emphasizing the excellent charge transfer ability of the [Ca_2_N]^+^·e^−^ electride that activates the Cu NPs as a catalyst. Furthermore, other tested Cu compounds, such as CuI and CuBr_2_ showed no reaction (entries 9 and 10), but their combined systems with the [Ca_2_N]^+^·e^−^ electride produced the product **3aa** in 91% yield (entries 11 and 12). These results indicate that Cu^+^ and Cu^2+^ cations of the CuI and CuBr_2_ compounds can be reduced by the excess electrons from the electride, probably forming negatively charged Cu clusters and acting as active sites for the catalytic sulfenylation.

**Table 1:** Results obtained from the catalytic sulfenylation of indole (**1a**) with various catalysts

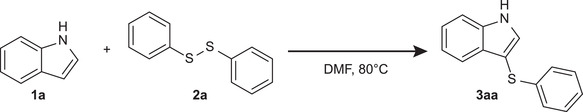			
Entry	Catalyst & reagent	Amount of catalyst^a)^	Yield^b)^
1	Cu NPs	1.0 eq	N.R.
2	[Ca_2_N]^+^·e^−^	1.0 eq	N.R.
3	Cu NPs/[Ca_2_N]^+^·e^−^	1.0 eq	93%
4	Cu NPs/[Ca_2_N]^+^·e^−^	0.75 eq	92%
5	Cu NPs/[Ca_2_N]^+^·e^−^	0.5 eq	85%
6	Cu NPs + Sodium ascorbate	1.0 eq	N.R.
7	Cu NPs + Zn dust	1.0 eq	N.R.
8	CuI + Zn dust	1.0 eq	N.R.
9	CuI	1.0 eq	N.R.
10	CuBr_2_	1.0 eq	N.R.
11	CuI + [Ca_2_N]^+^·e^−^	1.0 eq	91%
12	CuBr_2_ + [Ca_2_N]^+^·e^−^	1.0 eq	91%

^a)^ All reactions were carried out at 0.1 mmol scales (**1a**: 0.1 mmol, **2a**: 0.1 mmol);

^b)^ The yield was determined by GC‐MS spectroscopy using *n*‐dodecane as the internal standard. N.R.: no reaction.

### 2.3 Negatively Charged and Non‐Oxidized Surface of Cu NPs

**Figure 2:**
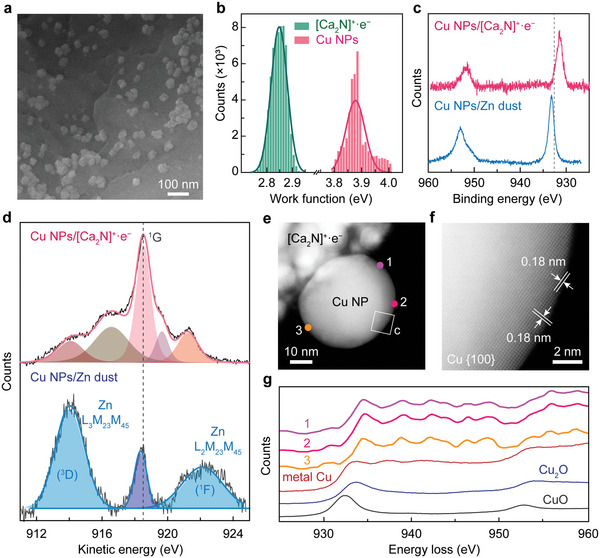
Negatively charged and non‐oxidized Cu NPs on the [Ca_2_N]^+^·e^−^ electride support. a) SEM image of Cu NPs on the surface of [Ca_2_N]^+^·e^−^ electride. b) Histogram of work function values for the Cu NPs and [Ca_2_N]^+^·e^−^ measured by KPFM. c) XPS spectra of Cu 2*p* for the Cu NPs on Zn dust (blue) and [Ca_2_N]^+^·e^−^ electride (red) supports. Vertical dashed line is the binding energy of Cu 2*p*_3/2_ of Cu metal. d) Auger spectra of Cu L_3_M_45_M_45_ of Cu NPs on the Zn dust (blue) and [Ca_2_N]^+^·e^−^ electride (red) supports. e,f) High‐resolution STEM images of a Cu NP on the [Ca_2_N]^+^·e^−^ electride e), showing the non‐oxidized surface structure as confirmed by the interplanar distance of 0.18 nm of *fcc* Cu {100} plane at the outermost layer f). g) EELS data from the surface region of positions 1−3 in e), showing no white lines of Cu oxides.

To elucidate the origin of the catalytic sulfenylation, we characterize the Cu NPs supported by the [Ca_2_N]^+^·e^−^ electride and compare its electronic and surface state with those of the commercial Cu NPs, the separated Cu NPs from the electride,^[^ ^41^ ^]^ and Cu NPs supported by Zn dust. **Figure** **2**a shows the scanning electron microscopy (SEM) image of the synthesized Cu NPs on [Ca_2_N]^+^·e^−^ electride by solid‐state reaction (see the Experimental Section), where Cu NPs with a 30 ± 7 nm diameter form on the surface of [Ca_2_N]^+^·e^−^ electride. MSI‐induced charge transfer significantly changed the *ϕ* value of the Cu NPs. The *ϕ* histograms obtained with Kelvin probe force microscopy (KPFM) show an average value of ≈3.9 eV for the Cu NPs, which is lower than the *ϕ* value of Cu metal (4.5 eV),^[^ ^42^ ^]^ suggesting that the electride with a *ϕ* of ≈2.9 eV provides excess electrons to the grown Cu NPs on the surface (Figure 2b). It is remarkable that all the measured *ϕ* value of Cu NPs are below 4.0 eV, highlighting the excellent electron donating ability of the [Ca_2_N]^+^·e^−^ electride. The transferred excess electrons are accumulated at the surface of metallic Cu NPs and consequently affect the surface electronic state. Moreover, 3D profile for the work function of the heterogeneous catalyst of Cu NPs on [Ca_2_N]^+^∙e^−^ electride having low work function region of ≈3.9 eV for the negatively charged Cu NPs is provided in Figure S1, Supporting Information.

From the X‐ray photoelectron spectroscopy (XPS) measurements (Figure 2c), it is revealed that the binding energy of Cu atom for the Cu NPs/[Ca_2_N]^+^·e^−^ shows a negative shift from metallic Cu (932.6 eV, vertical dotted line) of Cu metal, while a slight positive shift for the Cu NPs/Zn dust is observed. For a comparison, XPS spectrum of Cu foil is provided in Figure S2, Supporting Information. This negative shift was also observed for the separated Cu NPs from the electride.^[^ ^41^ ^]^ In addition, the Cu compounds combined with the [Ca_2_N]^+^·e^−^ electride, which showed an efficient catalytic sulfenylation (entries 11 and 12 of Table 1) are also examined (Figure S3, Supporting Information). The observed peaks of Cu 2*p*_1/2_ and 2*p*_3/2_ are located at the energy range close to those of Cu metal, indicating that Cu^+^ and Cu^2+^ cations of the CuI and CuBr_2_ compounds are reduced by the excess electrons transferred from the [Ca_2_N]^+^·e^−^ electride.

The negatively charged state of Cu NPs induced by the surface‐accumulated excess electrons is beneficial in preventing the oxidation of Cu NPs and keeping the surface in a metallic state. As shown in the Cu L_3_M_45_M_45_ Auger spectrum (Figure 2d), the Cu NPs grown on the [Ca_2_N]^+^·e^−^ electride shows the characteristic ^1^G peak of Cu metal at 918.6 eV, indicating a non‐oxidized surface state.^[^ ^43^, ^44^ ^]^ In contrast, the Auger spectrum of Cu NPs combined with Zn dust support shows the features of surface oxidation, with a broad peak. This non‐oxidized surface was further confirmed by the annular dark‐field scanning transmission electron microscopy (ADF‐STEM) image, which shows the face‐centered cubic (*fcc*) structure at the surface with an interplanar distance of 0.18 nm, which corresponds to the *fcc* Cu {100} plane (Figure 2e,f). Moreover, electron energy loss spectroscopy (EELS) verified the absence of copper oxide moieties at the surface. The energy‐loss near‐edge structures (ELNES) spectrum of Cu L edges at the surface region of Cu NPs/[Ca_2_N]^+^·e^−^ (positions 1−3 in Figure 2e) corresponds to that of Cu metal, without any white‐lines of copper oxides (Figure 2g). In contrast, STEM observations of the commercial Cu NPs and Cu NPs supported by Zn dust revealed that the surface area of both Cu NPs was clearly oxidized (Figures S4 and S5, Supporting Information). However, the separated Cu NPs from the electride, which showed no sulfenylation, also maintained the negatively charged surface state as well as non‐oxidized surface.^[^ ^41^ ^]^ Considering the difference between the Cu NPs grown on the electride and separated from the electride, where both NPs are negatively charged, the number of surface‐accumulated excess electrons is probably responsible for triggering the reduction of **1a** and **2a**, and their bond formation for the completion of catalytic sulfenylation. From the previously established relation between the excess charge number per surface Cu atom and *ϕ* value,^[^ ^16^ ^]^ we obtained the excess charge of 0.9e^−^ and 1.6e^−^ for Cu NPs separated from the electride and grown on the electride, respectively. It is thus concluded that the non‐oxidized metallic surface with substantially high‐density excess electrons is the key element for activating the catalytic sulfenylation on the Cu NPs supported by the [Ca_2_N]^+^·e^−^ electride. Nevertheless, the critical number of excess electrons per surface Cu atom for the catalytic sulfenylation is still ambiguous, requiring a further study to give more clearer role of negatively charged Cu state with surface‐accumulated excess electrons.

### 2.4 Proposed Mechanism for the Indole Sulfenylation

**Figure 3:**
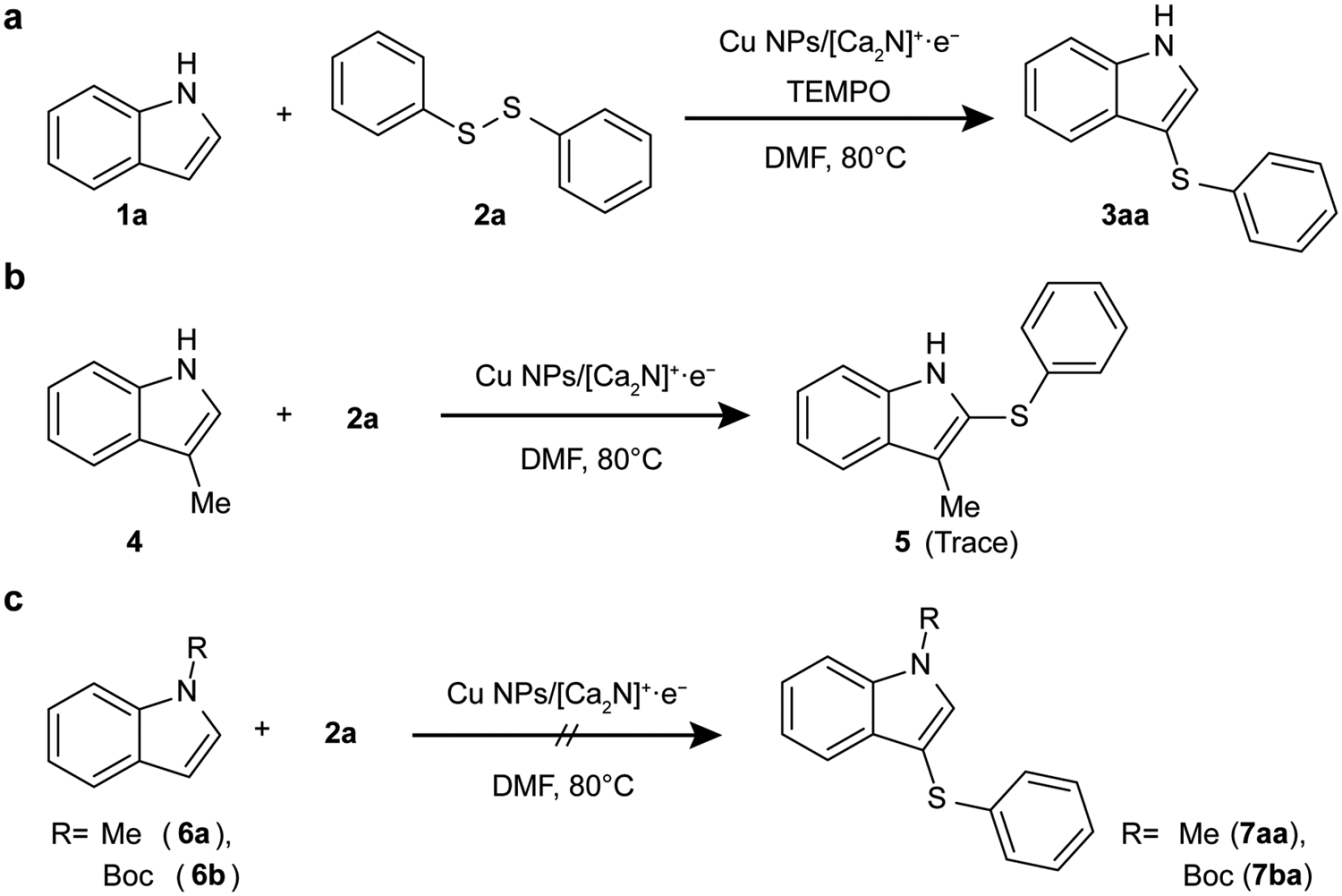
Mechanistic study for the sulfenylation of (aza)indoles with disulfide by the Cu NPs/[Ca_2_N]^+^·e^−^ catalyst. a) Reaction between **1a** and **2a** in the presence of a radical scavenger, TEMPO. b) Reaction between **2a** and 3‐methyl indole (**4**). c) Reactions of **2a** and N‐substituted indoles **6a** and **6b**.

**Figure 4:**
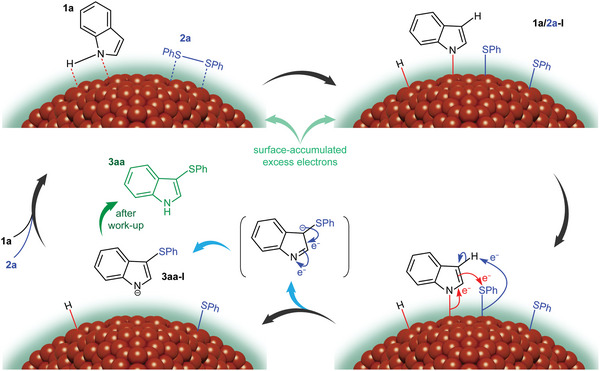
Proposed mechanism for the sulfenylation of indoles, using the depicted molecules **1a** and **2a** as examples.

We performed a series of experiments to examine the mechanism of the Cu NPs/[Ca_2_N]^+^·e^−^‐mediated sulfenylation of (aza)indoles with disulfide (**Figure** **3**). First, the reaction between **1a** and **2a** was conducted under the optimized conditions in the presence of a radical scavenger, TEMPO. The lack of reaction suppression under these conditions indicated the absence of radical species intermediates in this process (Figure 3a). As previously mentioned, the reaction regioselectively generates 3‐sulfenyl (aza)indoles. We tested the reaction with 3‐methyl indole (**4**), with the 3‐position blocked, and observed that the sulfenylation did not occur at the 2‐position (Figure 3b). Finally, we evaluated the use of N‐protected indoles and concluded that the reaction requires the presence of the free N—H functionality, since the reactions of N‐substituted indoles **6a** and **6b** did not proceed (Figure 3c). The proposed mechanism for the sulfenylation of indoles, based on the above results, is schematically illustrated in **Figure** **4**, using **1a** and **2a** as the examples. Both free N—H indole **1a** and disulfide **2a** are adsorbed on the surface of the Cu NPs where addition of Cu into N—H and S—S bonds generates the activated species **1a/2a‐I**. The increased electron density of indole moiety by its coordination to the negatively charged Cu species can lead to the facile sulfenylation with the ‐SPh moiety at the surface of Cu NPs. Immediately, deprotonation process occurs fast to afford the sulfenylated indole species **3aa‐I**. The Cu‐catalytic cycle repeats efficiently and the fact that the use of 0.75 equiv. of **2a** did not lower down the yield (entry 9, Table S1, Supporting Information) indicates that both sulfenyl moieties in disulfide reagent can participate in the process, showing the high atom‐economy of the transformation. Finally, protonation of **3aa‐I** upon aqueous work‐up generates product **3aa**.

### 2.5 Scope of Sulfenylation Using Various Disulfide and Indole Derivatives

Furthermore, we investigated the substrate scope of the sulfenylation of indole derivatives (**Table** **2**). The reactions of various aryl disulfide derivatives proceeded under the optimal conditions, regardless of the electron density and substituent position, generating the corresponding 3‐arylthioindole products in high yields. Reactions of substrates with electron‐donating substituents, such as *p*‐methyl (**2b**), *p*‐methoxy (**2c**), and *o*‐amino (**2d**) groups as well as electron‐withdrawing substituents, such as *p*‐chloro (**2e**), *m*‐trifluoromethyl (**2g**), and *o*‐bromo (**2f**) furnished the corresponding 3‐arylthioindoles (**3**). Importantly, the reactions were highly regioselective with respect to the 3‐sulfenyl products. The reactions of substituted indoles (**1b**, **1c**, **1d**, **1e**) afforded the corresponding 3‐arylthioindole products (**3**) in good yields. Notably, the mild reaction conditions tolerated various functional groups, such as —Cl, —Br, —CF_3_, —NO_2_, and —NH_2_, in both **1** and **2**. The synthetic utility of the transformation was examined in a gram‐scale synthesis, where the product **3aa** was prepared on a 5 mmol scale, and the yield was similar to that obtained on the 0.2 mmol scale reaction. Upon investigation of the indole framework scope, we extended the transformation to 7‐azaindole (**8**) (Table S3, Supporting Information). The reactions of **8** with various diaryl disulfides (**2**) containing both electron‐donating and withdrawing groups effectively proceeded under the standard conditions to generate the corresponding 3‐arylthioazaindoles (**9**) in high yields.

**Table 2:** Substrate scope of the sulfenylation of indole derivatives.^a),b)^

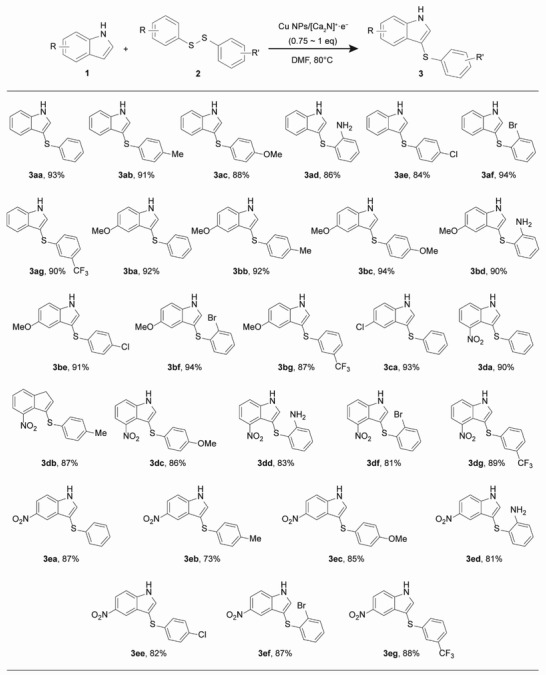

^a)^ All reactions were carried out at 0.2 mmol scales (**1**: 0.2 mmol, **2**: 0.15 mmol);

^b)^ Isolated yields.

## 3 Conclusion

A heterogeneous catalytic system of negatively charged Cu NPs grown on [Ca_2_N]^+^·e^−^ electride support has been developed for the regioselective sulfenylations of (aza)indoles, producing various 3‐arylthio(aza)indoles in a high yield. While the catalytic activity of the commercial Cu NPs and negatively charged Cu NPs separated from the electride for the sulfenylation has been of scarce, the negatively charged surface of the Cu NPs supported by the [Ca_2_N]^+^·e^−^ electride, which has excess charge of 1.6e^−^ per surface Cu atom, shows the efficient catalytic reactions in the absence of any additives. In particular, the critical role of substantially high‐density surface excess electrons on the negatively charged Cu NPs to trigger the catalytic reactions was evidently demonstrated from the comparative experiments and mechanistic studies. We believe that our protocol represents a practical sulfenylation reaction and provides a milestone for the application of the unique electride support with excellent electron donating ability and negatively charged Cu NPs in synthetic chemistry. Because in principle the metal NPs can be charged in a negative state by the charge transfer via a physical contact with an electride support having a lower *ϕ* value than that of the metal NPs, this work will stimulate the further utilization of negatively charged metal NPs supported by the electride for multiple electrons involving catalytic reactions.

## 4 Experimental Section

### Catalyst Preparation

All the synthesis processes were carried out in glove boxes filled with high‐purity argon gas (Ar 99.999%) to minimize the oxidation of raw materials and synthesized [Ca_2_N]^+^·e^−^ electride. To synthesize a stoichiometric polycrystalline [Ca_2_N]^+^·e^−^ electride, Ca_3_N_2_ powder and Ca shots in a 1:1 ratio was mixed, and then pressed them into a pellet form. The pelletized mixture was annealed at 800 °C for 48 h under vacuum (≈10^−3^ Pa) in a silica tube. After annealing, the sample was quenched into water. The synthesized sample was pulverized into a powder in an agate mortar and reannealed under the same conditions to improve homogeneity. To synthesized the Cu NPs on the [Ca_2_N]^+^·e^−^ electride, polycrystalline [Ca_2_N]^+^·e^−^ powder (5.31 mmol), and dried Cu(II) acetate (0.0786 mmol) were mixed with *n*‐heptane in an agate mortar in the glove box and transferred into a silica tube for heating at 150 °C under vacuum (≈10^−3^ Pa) for 24 h. The synthetic procedures of separated Cu NPs from the electride can be found in the previous report.^[^ ^41^ ^]^

### General Experimental Procedure for Sulfenylation of (aza)indole Derivatives

An oven‐dried resealable test tube, equipped with a magnetic stir bar, was charged with (aza)indoles derivatives (0.2 mmol), and aryl disulfide derivatives (0.15 mmol). In a nitrogen‐filled glove box, the reaction mixture was charged with Cu NPs/[Ca_2_N]^+^·e^−^ (15 mg, 0.15 mmol; corresponds to 0.022 mmol of Cu) and dimethylformamide, DMF (1 mL). The reaction was allowed to proceed at 80 °C and the progress was monitored using thin layer chromatography or gas chromatography. After completion (~ 4 h), the reaction mixture was then diluted with EtOAc and washed with water. The organic layers were dried over MgSO_4_, concentrated in vacuo, and purified by silica gel flash column chromatography using a hexane‐ethyl acetate mixture as the eluent to give the corresponding 3‐arylthio(aza)indole product.

### Characterization

The morphology of Cu NPs/[Ca_2_N]^+^·e^−^ was analyzed using field‐emission SEM (JEOL, JSM‐7600F). STEM‐EELS measurements were performed using a Cs‐corrected TEM (JEM‐ARM200F, JEOL) operating at 200 kV. All samples were dispersed in n‐heptane, applied to a 300‐mesh gold holey carbon grid (TED PELLA INC.) in glovebox, and dried in a vacuum chamber. To prevent oxidation of the STEM sample, a single‐tilt vacuum transfer holder (Model VTST‐4006, Gatan) was used. EELS measurements were performed in the STEM mode using the same microscope equipped with a Gatan imaging filter (GIF) detector (Gatan Quantum 965ER). XPS and AES measurements were performed to investigate chemical and electronic states of Cu NPs on the surface of [Ca_2_N] ^+^∙e^−^ electride. The photoemission results were obtained using a Scienta R4000 electron analyzer with an Al K*α* X‐ray source (1486.7 eV) and a discharge lamp that emitted an excitation line of He I*α* (21.2 eV). Core spectrum of Cu 2*p* and Auger spectrum of Cu L_3_M_45_M_45_ were measured for analysis, and all spectra were fitted by comparing the binding energies of the component peaks with CasaXPS. AES was modeled by a symmetric mixed Gaussian–Lorentzian function. Every peak was calibrated by the spectrum of hydrocarbon on the sample and reference Au attached next to the sample. The surface work function of the Cu NPs/[Ca_2_N]^+^·e^−^ placed on an Au‐coated Si substrate was measured by an atomic force microscope (MFP‐3D AFM, Asylum Research) equipped with a sealed electrochemistry cell filled with argon gas. The KPFM experiments were conducted using Ti/Ir‐coated Si probe (ASYELEC.01‐R2, Asylum Research) with a force constant of 2.8 N m^−1^ and a resonance frequency of 75 kHz. The work function of the probe was calibrated using a highly ordered pyrolytic graphite (HOPG) as a reference with the work function of ≈4.6 eV.^[^ ^45^ ^]^ During the KPFM scanning process, the scan rate and set point were 0.8 Hz and 0.5 V, respectively. Moreover, an a.c. voltage *V*_a.c._  =  1 V was applied to the tip, and the tip was lifted up 30 nm from the sample surface.

### Statistical Analysis

All statistical analyses of the STEM‐EELS spectra, KPFM data, XPS, and AES spectra were performed using the Origin software.

## Conflict of Interest

The authors declare no conflict of interest.

## Supporting information

Supporting Information

## Data Availability

The data that support the findings of this study are available from the corresponding author upon reasonable request.
