# Supplementary material for: Boosted Heterogeneous Catalysis by Surface‐Accumulated Excess Electrons of Non‐Oxidized Bare Copper Nanoparticles on Electride Support
Source: Adv Sci (Weinh). 2022 Nov 17;10(2):2204248. doi: 10.1002/advs.202204248 (PMC9839873; doi:10.1002/advs.202204248)
Supplement: Supplementary file 1 — Supporting Information [file ADVS-10-2204248-s001.pdf]

ADVANCED SCIENCE
Open Access

Supporting Information

for *Adv. Sci.*, DOI 10.1002/advs.202204248

Boosted Heterogeneous Catalysis by Surface-Accumulated Excess Electrons of Non-Oxidized Bare Copper Nanoparticles on Electride Support

Sung Su Han, Athira Thacharon, Jun Kim, Kyungwha Chung, Xinghui Liu, Woo-Sung Jang, Albina Jetybayeva, Seungbum Hong, Kyu Hyoung Lee, Young-Min Kim, Eun Jin Cho and Sung Wng Kim**

Supporting Information

Boosted Heterogeneous Catalysis by Surface-Accumulated Excess Electrons of Non-Oxidized Bare Copper Nanoparticles on Electride Support

Sung Su Han, Athira Thacharon, Jun Kim, Kyungwha Chung, Xinghui Liu, Woo-Sung Jang, Albina Jetybayeva, Seungbum Hong, Kyu Hyoung Lee, Young-Min Kim, Eun Jin Cho* and Sung Wng Kim*

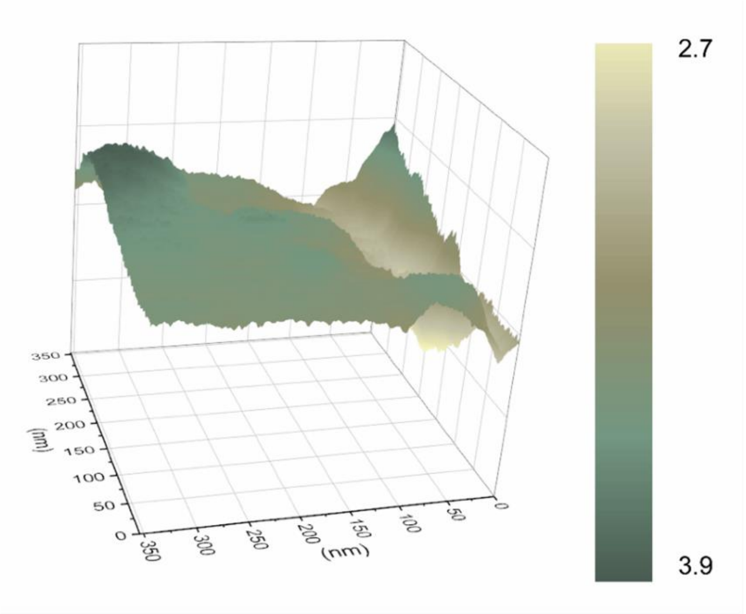

Figure S1: Work function mapping image of Cu NPs grown on $[Ca_2N]^+ \cdot e^-$ electride.

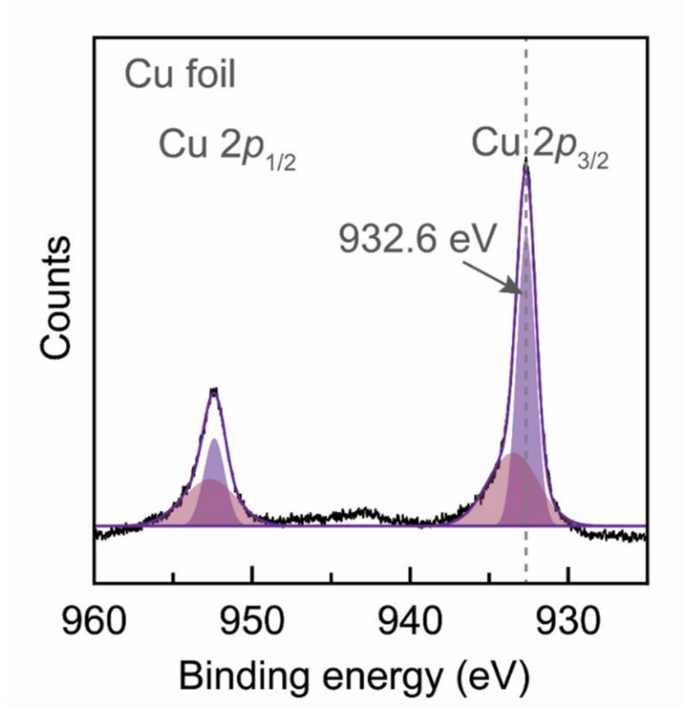

Figure S2: XPS spectrum of Cu foil.

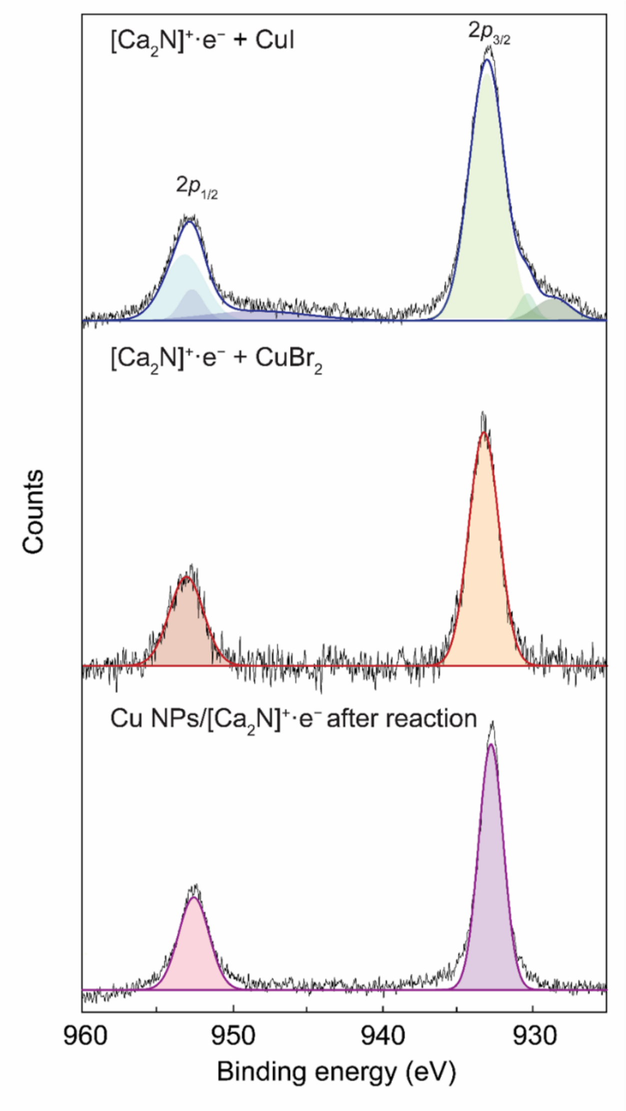

Figure S3: XPS measurement results of entry 11 ($[Ca_2N]^+\cdot e^-$ + CuI), entry 12 ($[Ca_2N]^+\cdot e^-$ + $CuBr_2$), and after reaction of entry 3 (Cu NPs/$[Ca_2N]^+\cdot e^-$) in Table 1.

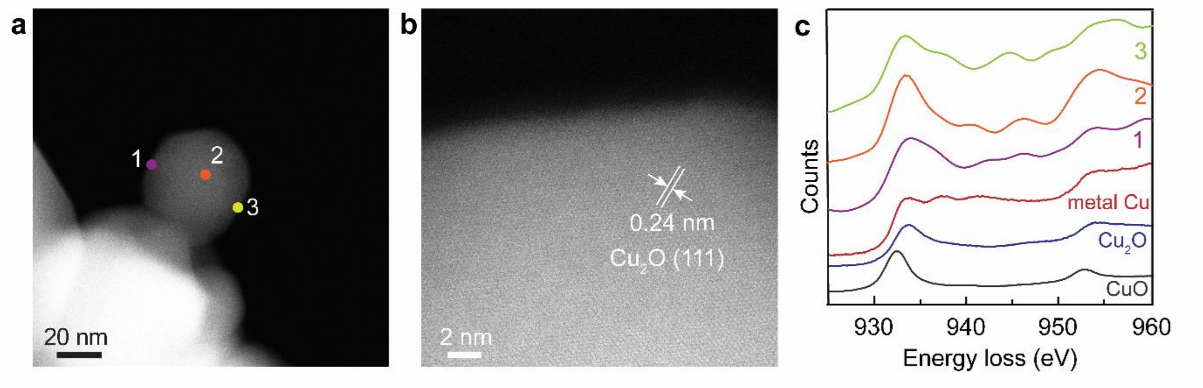

Figure S4: a) ADF-STEM image of commercial Cu NPs. b) High Resolution ADF-STEM image of commercial Cu NPs. c) EEL spectra from the marked positions 1–3 in a.

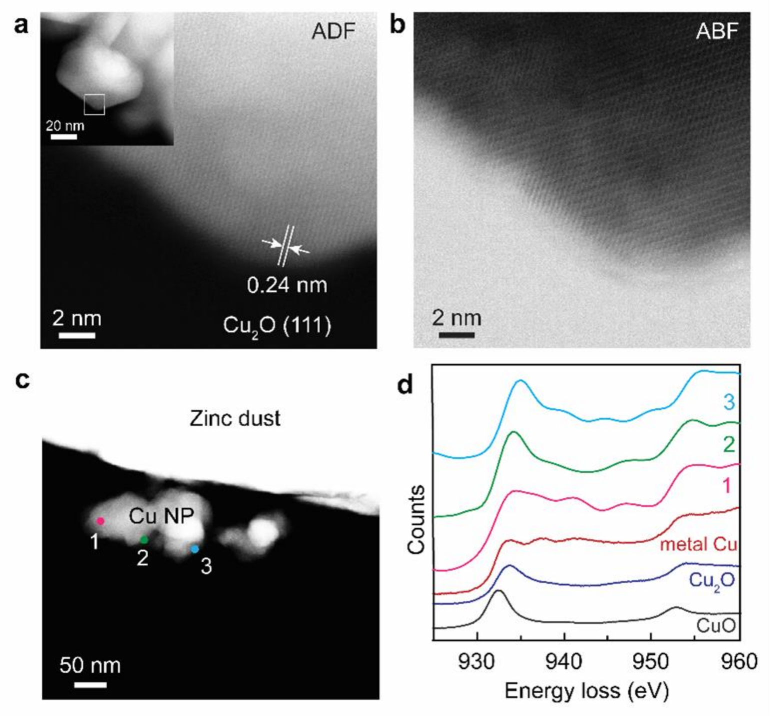

Figure S5: a) High resolution ADF-STEM image of Cu/Zn dust from boxed region in the top-left inset. b) High resolution ABF-STEM image of a. c-d) STEM image and EEL spectra from the marked positions 1–3 from c.

Table S1: Optimization of sulfenylation of **1a** using Cu NPs/$[Ca_2N]^+\cdot e^-$ catalyst[a].

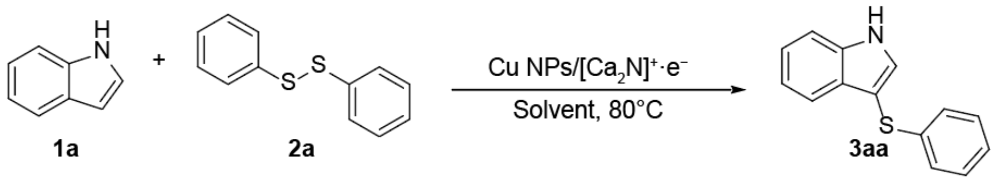

Entry	Solvent	Cu NPs/$[Ca_2N]^+\cdot e^-$	Variation	Yield[b]
1	DMF	1 equiv.		93%
2	DMSO	1 equiv.		71%
3	Ethanol	1 equiv.		N.R.
4	Acetonitrile	1 equiv.		16%
5	THF	1 equiv.		N.R.
6	DMF	1 equiv.	room temperature	N.R.
7	DMF	1 equiv.	50°C	Trace[c]
8	DMF	1 equiv.	0.5 equiv. **2a**	74%
9	DMF	1 equiv.	0.75 equiv. **2a**	91%

[a]All reactions were carried out on 0.1 mmol scales. [b]Yields were determined by GC-MS using *n*-dodecane as the internal standard. [c]Unidentified products were also observed. N.R: no reaction

We started the investigation with indole **1a** and diphenyl disulfide **2a** as the model substrates (Table S1). Remarkably, the sulfenylation of **1a** occurred in the presence of Cu NPs/$[Ca_2N]^+\cdot e^-$ (1 eq, 0.1 mmol of electride contain 15 mol% of Cu(0)) and **2a** in DMF to give 3-phenylthioindole **3a** in 93% yield at 80°C (entry 1). The reaction did not display improved reactivity in the other organic solvents (entries 2, 3, 4, and 5), and worked best at 80 °C (entries 6 and 7). The use of substoichiometric disulfide still delivered the product in good yields, indicating that the two −SPh groups in **2a** are utilized in the reaction, highlighting the efficiency of the transformation (entries 8 and 9). Notably, the reaction does not require any additives.

Table S2. Results obtained from the catalytic sulfenylation of indole (**1a**) with negatively charged Cu NPs seperated from the electride and heterogeneous system of Cu NPs grown on the electride.

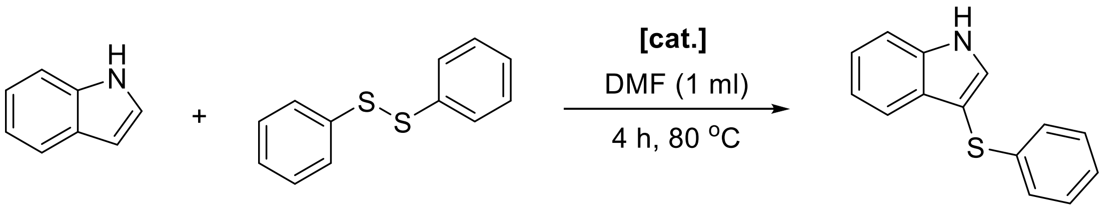

**1a**	**2a**	**3aa**
0.1 mmol	0.1 mmol	

**[cat.]**	catalyst loading	conversion[a]
CuNPs/e^-	1.4 mg (0.0225 mmol)	0%
CuNPs/e^-	5 mg	0%
CuNPs/e^-	10 mg	0%
CuNPs/$[Ca_2N]^{+\cdot}e^-$	15 mg	Full conversion

a) convsersion of **1a** into **3aa**. determined by GC-MS using *n*-dodecane as an internal standard.

Table S3: Substrate scope of the sulfenylation of 7-azaindole[a].

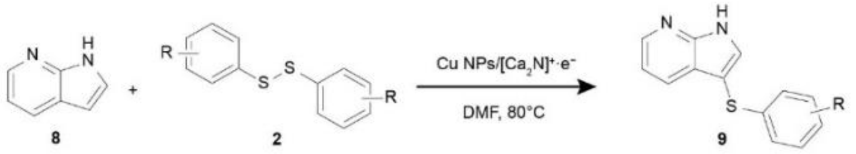

Entry	Dissulfide (2)	Product (9)	Yield[b]
1	**2a**	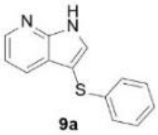 **9a**	95%
2	**2b**	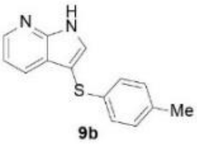 **9b**	90%
3	**2c**	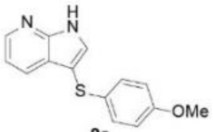 **9c**	86%
4	**2d**	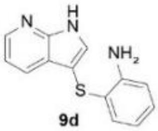 **9d**	91%
5	**2e**	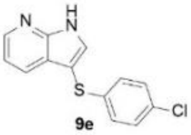 **9e**	84%
6	**2f**	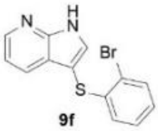 **9f**	90%
7	**2g**	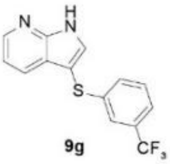 **9g**	85%

[a] All reactions were carried out at 0.2 mmol scales (**1**: 0.2 mmol, **2**: 0.15 mmol). [b] Isolated yields.

General analytical information

The synthesized 3-arylthio(aza)indole derivatives were characterized by ^{1}H NMR, ^{13}C NMR, ^{19}F NMR, and FT-IR spectroscopy. NMR spectra were recorded on a Varian 600 MHz instrument (600 MHz for ^{1}H NMR, 151 MHz for ^{13}C NMR, and 564 MHz for ^{19}F NMR). Copies of ^{1}H and ^{13}C NMR spectra can be found at the end of the Supporting Information. ^{1}H NMR experiments are reported in units, parts per million (ppm), and were measured relative to residual chloroform (7.26 ppm) or dimethyl sulfoxide (2.50 ppm) in the deuterated solvent. ^{13}C NMR spectra are reported in ppm relative to deuterochloroform (77.23 ppm) or dimethyl sulfoxide (39.52 ppm), and all were obtained with ^{1}H decoupling. ^{19}F NMR spectra are reported in ppm, and all were taken composite pulse decoupling (CPD) mode. Coupling constants were reported in Hz. FT-IR spectra were recorded on a Nicolet 6700 Thermo Scientific FT-IR spectrometer. Reactions were monitored by GC-MS of the crude reaction mixture using dodecane as internal standard and products were detected by GC-MS using the Agilent GC 7890B/5977A inert MSD with Triple-Axis Detector. Mass spectral data of all unknown compounds were acquired at the Korea Basic Science Institute (Daegu) on a Jeol JMS 700 high-resolution mass spectrometer. A quadrupole mass analyzer was used for HRMS measurements.

Analytic Data for 3-arylthio(aza)indoles.

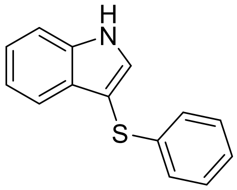

3-(phenylthio)-1H-indole, **3aa**[2]: white solid; **^{1}H NMR (600 MHz, $CDCl_3$)** δ 8.38 (s, 1H), 7.63 (dd, *J* = 7.2, 1.3 Hz, 1H) , 7.49 (d, *J* = 2.6 Hz, 1H), 7.45 (d, *J* = 8.1 Hz, 1H), 7.28 (ddd, *J* = 8.1, 7.8, 1.1 Hz, 1H), 7.18 (dd, *J* = 7.9, 1.1 Hz, 1H), 7.16 (ddd, *J* = 7.8, 7.2, 1.3 Hz, 2H), 7.12 (dd, *J* = 7.8, 1.3 Hz, 2H), 7.06 (ddd, *J* = 7.9, 7.8, 1.1 Hz, 1H); **^{13}C NMR (151 MHz, $CDCl_3$)** δ = 139.42, 136.70, 130.84, 129.32, 128.89, 126.08, 124.98, 123.27, 121.13, 119.90, 111.76, 103.13; **IR (neat):** ν_{max} = 3413, 3127, 3052, 1648, 1580, 1472, 1436, 1407, 1232, 735 cm^{-1}; $\boldsymbol{R_f}$ = 0.51 (hex/EtOAc 2/1).

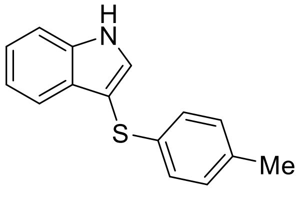

3-(p-tolylthio)-1H-indole, **3ab**[2]: white solid; **^{1}H NMR (600 MHz, $CDCl_3$)** δ 8.30 (s, 1H), 7.65 (d, *J* = 7.9 Hz, 1H), 7.45 (s, 1H), 7.42 (d, *J* = 8.2 Hz, 1H), 7.30 – 7.27 (dd, *J* = 8.0, 7.9 Hz, 1H), 7.20 – 7.17 (dd, *J* = 8.2, 8.0 Hz, 1H), 7.07 (d, *J* = 8.1 Hz, 2H), 7.00 (d, *J* = 8.1 Hz, 2H),

2.28 (s, 3H); **^{13}C NMR (151 MHz, CDCl$_3$)** δ = 136.46, 135.48, 134.68, 130.43, 129.50, 129.11, 126.29, 122.98, 120.84, 119.68, 111.55, 103.50, 20.87; **IR (neat):** ν_{max} = 3044, 1490, 1454, 1406, 805, 744 cm^{-1}; ***R$_f$*** = 0.53 (hex/EtOAc 2/1).

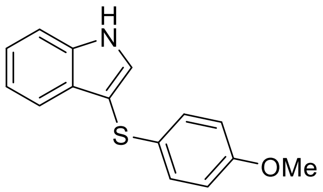

3-((4-methoxyphenyl)thio)-1H-indole, **3ac**[2]: white solid; **^{1}H NMR (600 MHz, CDCl$_3$)** δ 8.30 (s, 1H), 7.66 (d, *J* = 7.9 Hz, 1H), 7.42 (s, 1H), 7.39 (d, *J* = 8.2 Hz, 1H), 7.27 (dd, *J* = 7.9, 7.1 Hz, 1H), 7.19 (dd, *J* = 8.2, 7.1 Hz, 1H), 7.16 (d, *J* = 8.8 Hz, 2H), 6.76 (d, *J* = 8.8 Hz, 2H), 3.74 (s, 3H); **^{13}C NMR (151 MHz, CDCl$_3$)** δ = 157.94, 136.61, 130.24, 129.69, 129.15, 128.74, 123.10, 120.94, 119.77, 114.69, 111.73, 104.66, 55.51; **IR (neat):** ν_{max} = 3404, 1492, 1454, 1285, 1240, 1176, 1029, 823, 746 cm^{-1}; ***R$_f$*** = 0.44 (hex/EtOAc 2/1).

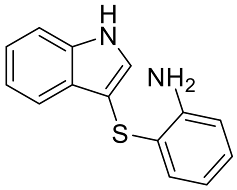

2-((1H-indol-3-yl)thio)aniline, **3ad**[4]: white solid; **^{1}H NMR (600 MHz, CDCl$_3$)** δ 8.34 (s, 1H), 7.69 (d, *J* = 7.8 Hz, 1H), 7.32 (d, *J* = 7.9 Hz, 1H), δ 7.31 (s, 1H), 7.23 (dd, *J* = 7.7,7.5 Hz, 1H), 7.22 (d, *J* = 7.9 Hz, 1H), 7.16 (dd, *J* = 7.8, 7.4 Hz, 1H), 7.03 (dd, *J* = 7.9, 7.5 Hz, 1H), 6.69 (d, *J* = 7.9 Hz, 1H), 6.63 (dd, *J* = 7.9, 7.4 Hz, 1H), 4.00 (s, 2H); **^{13}C NMR (151 MHz, CDCl$_3$)** δ = 145.68, 136.49, 132.06, 129.29, 128.89, 128.19, 122.99, 121.04, 120.81, 119.55, 119.15, 115.61, 111.77, 104.23; **IR (neat):** ν_{max} = 3399, 2924, 1609, 1477, 1452, 744 cm^{-1}; ***R$_f$*** = 0.56 (hex/EtOAc 1/1).

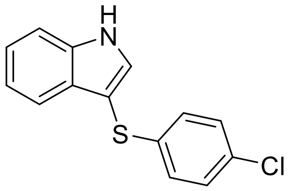

3-((4-chlorophenyl)thio)-1H-indole, **3ae**[2]: white solid; **^{1}H NMR (600 MHz, CDCl$_3$)** δ 8.40 (s, 1H), 7.58 (d, *J* = 7.9 Hz, 1H), 7.49 (s, 1H), 7.45 (d, *J* = 7.9 Hz, 1H), 7.29 (dd, *J* = 7.9, 7.3 Hz, 1H), 7.19 (dd, *J* = 7.9 , 7.3 Hz, 1H), 7.13 (d, *J* = 8.5 Hz, 2H), 7.03 (d, *J* = 8.5 Hz, 2H); **^{13}C NMR (151 MHz, CDCl$_3$)** δ = 138.01, 136.71, 130.88, 130.76, 129.01, 128.96, 127.33, 123.43, 121.28, 119.72, 111.86, 102.72; **IR (neat):** ν_{max} = 3392, 1739, 1474, 1091, 1009, 814, 750 cm^{-1}; ***R$_f$*** = 0.50 (hex/EtOAc 2/1).

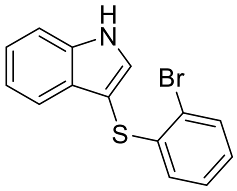

3-((2-bromophenyl)thio)-1H-indole, **3af**[3]: white solid; **^{1}H NMR (600 MHz, CDCl$_3$)** δ 8.47 (s, 1H), 7.61 (d, *J* = 7.9 Hz, 1H), 7.51 (d, *J* = 7.8 Hz, 1H), 7.50 (s, 1H), 7.47 (d, *J* = 8.1 Hz, 1H), 7.31 (dd, *J* = 8.1, 7.4 Hz, 1H), 7.20 (dd, *J* = 7.8, 7.4 Hz, 1H), 6.98 (dd, *J* = 7.9, 7.4 Hz, 1H), 6.92 (dd, *J* =

7.7, 7.4 Hz, 1H), 6.65 (d, J = 7.7Hz, 1H); **^{13}C NMR (151 MHz, $CDCl_3$)** δ = 140.61, 136.76, 132.71, 131.49, 129.07, 127.70, 126.60, 125.86, 123.46, 121.34, 119.80, 119.77, 111.93, 102.05; **IR (neat):** ν_{max} = 3409, 1444, 1427, 1406, 1236, 1018, 743 cm^{-1}; ***R*$_f$** = 0.47 (hex/EtOAc 2/1).

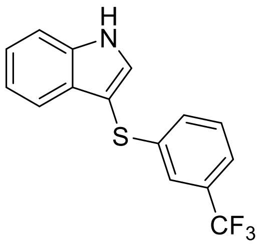

3-((3-(trifluoromethyl)phenyl)thio)-1H-indole, **3ag**[3]: colorless liquid; **^{1}H NMR (600 MHz, $CDCl_3$)** δ 8.44 (s, 1H), 7.62 (d, J = 7.6 Hz, 1H), 7.50 (s, 1H), 7.47 (s, 1H), 7.46 (d, J = 8.3 Hz, 1H), 7.33 (d, J = 7.2 Hz, 1H), 7.32 (dd, J = 7.2, 6.8 Hz, 1H), 7.25 (dd, J = 8.3, 7.6 Hz, 1H), 7.23 (d, J = 7.6 Hz, 1H), 7.22 (dd, 7.6, 7.4 Hz, 1H); **^{13}C NMR (151 MHz, $CDCl_3$)** δ = 141.18, 136.73, 131.20, 131.05 (q, $^2J_{C-F}$ = 32.2 Hz), 129.24, 129.00, 128.93, 123.93 (q, $^1J_{C-F}$ = 272.8 Hz), 123.51, 122.34 (q, $^3J_{C-F}$ = 3.6 Hz), 121.51 (q, $^3J_{C-F}$ = 3.6 Hz), 121.37, 119.56, 111.96, 101.72; **^{19}F NMR (564 MHz, $CDCl_3$)** δ = –62.6988; **IR (neat):** ν_{max} = 3406, 1738, 1322, 1166, 1125, 1073, 745 cm^{-1}; ***R*$_f$** = 0.45 (hex/EtOAc 2/1).

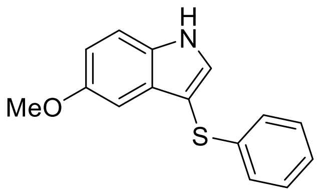

5-methoxy-3-(phenylthio)-1H-indole, **3ba**[2]: white solid; **^{1}H NMR (600 MHz, $CDCl_3$)** δ 8.35 (s, 1H), 7.44 (d, J = 2.6 Hz, 1H), 7.32 (d, J = 8.8 Hz, 1H), 7.18 (dd, J = 7.9, 7.7 Hz, 2H), 7.11 (d, J = 7.7 Hz, 2H), 7.07 (dd, J =7.4, 2.4 Hz, 1H), 7.06 (d, J = 7.9 Hz, 1H), 6.93 (d, J = 8.8, 2.6 Hz, 1H), 3.79 (s, 3H); **^{13}C NMR (151 MHz, $CDCl_3$)** δ = 155.13, 139.32, 131.36, 131.31, 129.96, 128.70, 125.67, 124.71, 113.59, 112.41, 102.20, 100.82, 55.79; **IR (neat):** ν_{max} = 3416, 1460, 1445, 1366, 1230, 1217, 740 cm^{-1}; ***R*$_f$** = 0.38 (hex/EtOAc 2/1).

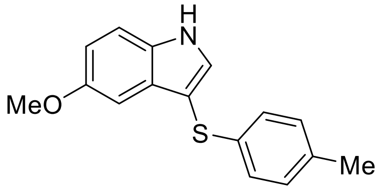

5-methoxy-3-(p-tolylthio)-1H-indole, **3bb**[4]: white solid; **^{1}H NMR (600 MHz, $CDCl_3$)** δ 8.32 (s, 1H), 7.39 (s, 1H), 7.29 (d, J = 8.8 Hz, 1H), 7.11 (s, 1H), 7.07 (d, J = 7.6 Hz, 2H), 7.02 (d, J = 7.6 Hz, 2H), 6.94 (d, J = 8.8 Hz, 1H), 3.81 (s, 3H), 2.29 (s, 3H); **^{13}C NMR (151 MHz, $CDCl_3$)** δ = 155.18, 135.77, 134.76, 131.53, 131.38, 130.12, 129.68, 126.20, 113.60, 112.61, 102.81, 101.02, 55.95, 21.00; **IR (neat):** ν_{max} = 3406, 1738, 1486, 1455, 1207, 1169, 804 cm^{-1}; ***R*$_f$** = 0.46 (hex/EtOAc 2/1).

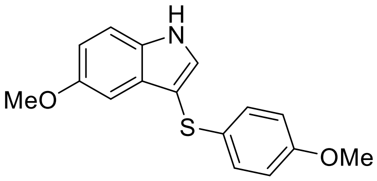

5-methoxy-3-((4-methoxyphenyl)thio)-1H-indole, **3bc**[5]: white solid; **^{1}H NMR (600 MHz, CDCl$_3$)** δ 8.34 (s, 1H), 7.39 (d, *J* = 2.4 Hz, 1H), 7.27 (d, *J* = 8.8 Hz, 1H), 7.14 (d, *J* = 8.8 Hz, 2H), 7.09 (s, 1H), 6.91 (dd, *J* = 8.8, 2.4 Hz, 1H), 6.76 (d, *J* = 8.8 Hz, 2H), 3.81 (s, 3H), 3.74 (s, 3H); **^{13}C NMR (151 MHz, CDCl$_3$)** δ = 157.87, 155.16, 131.53, 131.00, 130.01, 129.85, 128.41, 114.69, 113.54, 112.57, 103.94, 101.05, 55.97, 55.51; **IR (neat):** ν_{max} = 3406, 1491, 1457, 1439, 1285, 1241, 1207, 1170, 1031, 823 cm^{-1}; $\boldsymbol{R_f}$ = 0.37 (hex/EtOAc 2/1).

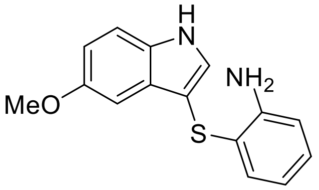

2-((5-methoxy-1H-indol-3-yl)thio)aniline, **3bd**[6]: white solid; **^{1}H NMR (600 MHz, CDCl$_3$)** δ 8.31 (s, 1H), 7.31 (d, *J* = 2.4 Hz, 1H), 7.21 (d, *J* = 8.8 Hz, 1H), 7.18 (dd, *J* = 7.8, 1.3 Hz, 1H), 7.12 (d, *J* = 2.4 Hz, 1H), 7.01 (ddd, *J* = 7.9, 1.3, 1.1 Hz, 1H), 6.87 (dd, *J* = 8.8, 2.4 Hz, 1H), 6.68 (dd, *J* = 7.9, 1.1 Hz, 1H), 6.63 (ddd, *J* = 7.8, 1.3, 1.1 Hz, 1H), 4.15 (s, 2H), 3.80 (s, 3H); **^{13}C NMR (151 MHz, CDCl$_3$)** δ = 155.04, 145.56, 131.67, 131.45, 129.95, 129.70, 128.05, 121.17, 119.14, 115.59, 113.44, 112.57, 103.53, 101.01, 55.96; **IR (neat):** ν_{max} = 3367, 2926, 1609, 1581, 1479, 1285, 1206, 1168, 1030, 780, 750 cm^{-1}; $\boldsymbol{R_f}$ = 0.53 (hex/EtOAc 1/1).

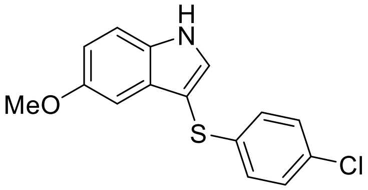

3-((4-chlorophenyl)thio)-5-methoxy-1H-indole, **3be**[7]: white solid; **^{1}H NMR (600 MHz, CDCl$_3$)** δ 8.40 (s, 1H), 7.44 (d, *J* = 2.5 Hz, 1H), 7.32 (d, *J* = 8.8 Hz, 1H), 7.13 (d, *J* = 8.7 Hz, 2H), 7.02 (d, *J* = 8.7 Hz, 2H), 7.02 (s, 1H), 6.94 (dd, *J* = 8.8, 2.5 Hz, 1H), 3.81 (s, 3H); **^{13}C NMR (151 MHz, CDCl$_3$)** δ = 155.40, 138.11, 131.57, 130.65, 129.87, 128.94, 127.09, 113.87, 112.73, 101.92, 101.91, 100.89, 56.00; **IR (neat):** ν_{max} = 3415, 1484, 1475, 1230, 1028, 1091, 1010, 814 cm^{-1}; $\boldsymbol{R_f}$ = 0.46 (hex/EtOAc 2/1).

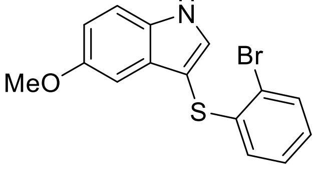

3-((2-bromophenyl)thio)-5-methoxy-1H-indole, **3bf**: white solid; m.p. 118-120 °C; **^{1}H NMR (600 MHz, CDCl$_3$)** δ 8.57 (s, 1H), 7.53 (d, *J* = 7.4 Hz, 1H), 7.43 (s, 1H), 7.33 (d, *J* = 8.8 Hz, 1H), 7.06 (s, 1H), 7.01 – 6.92 (m, 3H), 6.67 (d, *J* = 7.4 Hz, 1H), 3.80 (s, 3H); **^{13}C NMR (151 MHz, CDCl$_3$)** δ = 155.33, 140.54, 132.67, 132.13, 131.60, 129.87, 127.71, 126.42, 125.83, 119.64, 113.91, 112.85, 101.10, 100.78, 55.98; **IR (neat):** ν_{max} = 3419, 2970, 1444, 1366, 1229,

1216, 1206, 748 cm^{-1}; **HRMS** m/z (EI) calc. for **$C_{15}H_{12}BrNOS$** [M+] 334.9803, found 334.9796; ***R_f*** = 0.39 (hex/EtOAc 2/1).

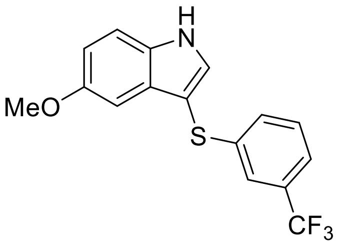

5-methoxy-3-((3-(trifluoromethyl)phenyl)thio)-1H-indole, **3bg**: colorless liquid; m.p. 69-71 ^{o}C; **^{1}H NMR (600 MHz, $CDCl_3$)** δ 8.45 (s, 1H), 7.46 (s, 1H), 7.43 (s, 1H), 7.34 (d, *J* = 8.8 Hz, 1H), 7.31 (d, *J* = 7.7 Hz, 1H), 7.25 (dd, *J* = 7.9, 7.7 Hz, 1H), 7.19 (d, *J* = 7.9 Hz, 1H), 7.03 (d, *J* = 2.4 Hz, 1H), 6.96 (dd, *J* = 8.8, 2.4 Hz, 1H), 3.81 (s, 3H); **^{13}C NMR (151 MHz, $CDCl_3$)** δ = 155.50, 141.25, 131.79, 131.62, 131.04 (q, $^{2}J_{C–F}$ = 32.3 Hz), 129.82, 129.26, 128.83, 123.92 (q, $^{1}J_{C–F}$ = 272.9 Hz), 122.18 (q, $^{3}J_{C–F}$ = 3.7 Hz), 121.44 (q, $^{3}J_{C–F}$ = 3.8 Hz), 114.01, 112.83, 101.07, 100.80, 56.01; **^{19}F NMR (564 MHz, $CDCl_3$)** δ = –62.7683; **IR (neat):** ν_{max} = 3405, 1582, 1485, 1323, 1207, 1167, 1124, 1072, 794 cm^{-1}; **HRMS** m/z (EI) calc. for **$C_{16}H_{12}F_3NOS$** [M+] 323.0592, found 323.0591; ***R_f*** = 0.44 (hex/EtOAc 2/1).

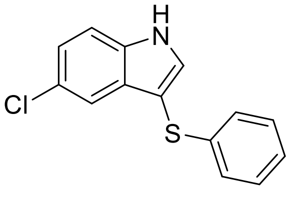

5-chloro-3-(phenylthio)-1H-indole, **3ca**[8]: white solid; **^{1}H NMR (600 MHz, $CDCl_3$)** δ 8.45 (s, 1H), 7.60 (d, *J* = 1.9 Hz, 1H), 7.49 (d, *J* = 2.4 Hz, 1H), 7.34 (d, *J* = 8.6 Hz, 1H), 7.22 (dd, *J* = 8.6, 1.9 Hz, 1H), 7.19 (t, *J* = 8.1, 7.5 Hz, 2H), 7.10 (d, *J* = 8.1 Hz, 2H), 7.09 (d, *J* = 7.5 Hz, 1H); **^{13}C NMR (151 MHz, $CDCl_3$)** δ = 138.69, 134.81, 132.01, 130.38, 128.81, 126.91, 125.91, 125.04, 123.53, 119.12, 112.68, 102.84; **IR (neat):** ν_{max} = 3398, 1579, 1475, 1445, 1099, 1023, 891, 869, 801, 691 cm^{-1}; ***R_f*** = 0.49 (hex/EtOAc 2/1).

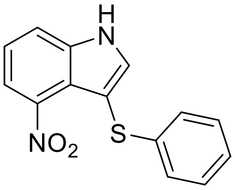

4-nitro-3-(phenylthio)-1H-indole, **3da**: yellow solid; m.p. 143-145 ^{o}C; **^{1}H NMR (600 MHz, DMSO-*d_6*)** δ 12.50 (s, 1H), 8.03 (s, 1H), 7.87 (dd, *J* = 8.1, 0.6 Hz, 1H), 7.69 (dd, *J* = 7.7, 0.6 Hz, 1H), 7.35 (dd, *J* = 8.1, 7.8 Hz, 1H), 7.18 (dd, *J* = 7.8, 7.6Hz, 2H), 7.05 (dd, *J* = 7.7, 7.6 Hz, 1H), 6.96 (d, *J* = 7.6 Hz, 2H); **^{13}C NMR (151 MHz, DMSO-*d_6*)** δ = 144.42, 142.44, 139.70, 139.18, 137.71, 128.76, 125.46, 124.83, 121.40, 119.31, 117.83, 116.82, 98.81; **IR (neat):** ν_{max} = 3403, 1738, 1366, 1217, 1050, 1025, 1004, 761, 732 cm^{-1}; **HRMS** m/z (EI) calc. for **$C_{14}H_{10}FN_2O_2S$** [M+] 270.0463, found 270.0460; ***R_f*** = 0.37 (hex/EtOAc 1/1).

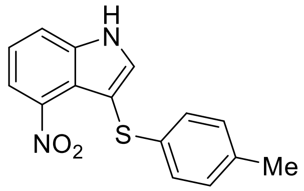

4-nitro-3-(p-tolylthio)-1H-indole, **3db**: yellow solid; m.p. 162-164 °C; **^{1}H NMR (600 MHz, DMSO-*d$_6$*)** δ 12.44 (s, 1H), 7.98 (s, 1H), 7.85 (d, *J* = 8.1 Hz, 1H), 7.67 (d, *J* = 7.7 Hz, 1H), 7.33 (dd, *J* = 8.1, 7.7 Hz, 1H), 6.99 (d, *J* = 8.3 Hz, 2H), 6.89 (d, *J* = 8.3 Hz, 2H), 2.18 (s, 3H); **^{13}C NMR (151 MHz, DMSO-*d$_6$*)** δ = 142.45, 139.13, 137.27, 135.87, 134.29, 129.42, 126.10, 121.31, 119.20, 117.75, 116.70, 99.73, 20.40; **IR (neat):** ν_{max} = 3352, 1738, 1518, 1363, 1333, 1229, 1217, 731 cm^{-1}; **HRMS** m/z (EI) calc. for $\mathbf{C_{15}H_{12}N_2O_2S}$ [M+] 284.0619, found 284.0618; $\boldsymbol{R_f}$ = 0.40 (hex/EtOAc 1/1).

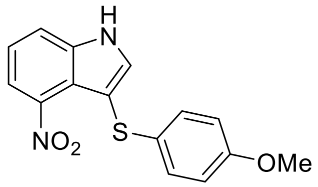

3-((4-methoxyphenyl)thio)-4-nitro-1H-indole, **3dc**: yellow solid; m.p. 147-149 °C; **^{1}H NMR (600 MHz, DMSO-*d$_6$*)** δ 12.37 (s, 1H), 7.92 (s, 1H), 7.83 (d, *J* = 8.1 Hz, 1H), 7.66 (d, *J* = 7.7 Hz, 1H), 7.31 (dd, *J* = 8.1, 7.7 Hz, 1H), 7.02 (d, *J* = 8.8 Hz, 2H), 6.80 (d, *J* = 8.8 Hz, 2H), 3.67 (s, 3H); **^{13}C NMR (151 MHz, DMSO-*d$_6$*)** δ = 157.64, 142.43, 139.06, 136.49, 129.21, 128.99, 121.23, 118.89, 117.70, 116.59, 114.59, 101.47, 55.10; **IR (neat):** ν_{max} = 3369, 2970, 1493, 1365, 1230, 1217 cm^{-1}; **HRMS** m/z (EI) calc. for $\mathbf{C_{15}H_{12}N_2O_3S}$ [M+] 300.0569, found 300.0571; $\boldsymbol{R_f}$ = 0.35 (hex/EtOAc 1/1).

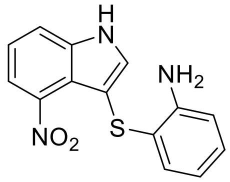

2-((4-nitro-1H-indol-3-yl)thio)aniline, **3dd**: yellow solid; m.p. 171-173 °C; **^{1}H NMR (600 MHz, DMSO-*d$_6$*)** δ 12.20 (s, 1H), 7.82 (d, *J* = 8.0 Hz, 1H), 7.72 (d, *J* = 7.8 Hz, 1H), 7.64 (s, 1H), 7.29 (dd, *J* = 8.0, 7.8 Hz, 1H), 6.97 (ddd, *J* = 8.1, 7.7, 1.2 Hz, 1H), 7.72 (dd, *J* = 7.4, 1.2 Hz, 1H), 6.68 (dd, *J* = 8.1, 0.9 Hz, 1H), 6.45 (ddd, *J* = 7.7, 7.4, 0.9 Hz, 1H), 5.21 (s, 2H); **^{13}C NMR (151 MHz, DMSO-*d$_6$*)** δ = 147.75, 142.05, 139.02, 133.94, 132.42, 128.47, 120.91, 118.58, 118.09, 118.05, 116.75, 116.67, 114.60, 102.41; **IR (neat):** ν_{max} = 2970, 1738, 1446, 1366, 1229, 1217 cm^{-1}; **HRMS** m/z (EI) calc. for $\mathbf{C_{14}H_{11}N_3O_2S}$ [M+] 285.0572, found 285.0569; $\boldsymbol{R_f}$ = 0.46 (hex/EtOAc 1/2).

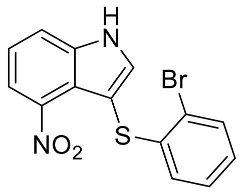

3-((2-bromophenyl)thio)-4-nitro-1H-indole, **3df**: yellow solid; m.p. 203-205 °C; **^{1}H NMR (600 MHz, DMSO-*d$_6$*)** δ 8.09 (s, 1H), 7.91 (d, *J* = 7.6 Hz, 1H), 7.76 (d, *J* = 7.7 Hz, 1H), 7.55 (d, *J* = 7.9 Hz, 1H), 7.39 (dd, *J* = 7.7, 7.4 Hz, 1H), 7.11 (dd, *J* = 7.6, 7.4 Hz, 1H), 6.99 (dd, *J* = 7.7, 7.9 Hz, 1H),

6.51 (d, J = 7.7 Hz, 1H); **^{13}C NMR (151 MHz, DMSO-d_6)** δ = 142.22, 141.21, 139.46, 138.62, 132.27, 132.15, 127.90, 125.94, 121.62, 119.48, 118.24, 117.25, 109.53, 97.41; **HRMS** m/z (EI) calc. for **$C_{14}H_9BrN_2O_2S$** [M+] 349.9548, found 349.9571; **IR (neat):** ν_{max} = 3415, 1050, 1024, 1004, 824, 761, 527 cm^{-1}; ***R$_f$*** = 0.34 (hex/EtOAc 1/1).

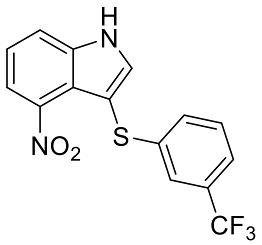

4-nitro-3-((3-(trifluoromethyl)phenyl)thio)-1H-indole, **3dg**: yellow liquid; m.p. 127-129 ^{o}C; **^{1}H NMR (600 MHz, DMSO-d_6)** δ 8.11 (s, 1H), 7.90 (d, J = 7.2 Hz, 1H), 7.72 (d, J = 6.6 Hz, 1H), 7.40 (s, 1H), 7.38 (d, J = 7.2, 6.6 Hz, 1H), 7.36 (d, J = 7.3 Hz, 1H), 7.25 (d, J = 7.1 Hz, H), 7.24 (dd, J = 7.3, 7.1 Hz, 1H); **^{13}C NMR (151 MHz, DMSO-d_6)** δ = 142.37, 142.16, 139.40, 138.47, 129.82, 129.64 (q, $^{2}J_{C–F}$ = 31.2 Hz), 129.10, 123.97 (q, $^{1}J_{C–F}$ = 273.3 Hz), 121.74, 121.45 (q, $^{3}J_{C–F}$ = 2.6 Hz), 121.25 (q, $^{3}J_{C–F}$ = 2.1 Hz), 119.35, 118.24, 117.23, 97.28; **^{19}F NMR (564 MHz, DMSO-d_6)** δ = –61.5080; **IR (neat):** ν_{max} = 3347, 2970, 1519, 1365, 1323, 1217, 1125, 791, 733 cm^{-1}; **HRMS** m/z (EI) calc. for **$C_{15}H_9F_3N_2O_2S$** [M+] 338.0337, found 338.0338; ***R$_f$*** = 0.39 (hex/EtOAc 1/1).

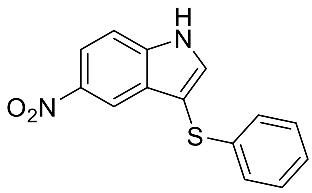

5-nitro-3-(phenylthio)-1H-indole, **3ea**[2]: yellow solid; **^{1}H NMR (600 MHz, CDCl$_3$)** δ 8.97 (s, 1H), 8.57 (d, J = 2.0 Hz, 1H), 8.17 (dd, J = 9.0, 2.0 Hz, 1H), 7.66 (s, 1H), 7.50 (d, J = 9.0 Hz, 1H), 7.19 (dd, J = 7.9, 7.4 Hz, 2H), 7.13 (d, J = 7.4 Hz, 2H), 7.13 (d, J = 7.9 Hz, 1H); **^{13}C NMR (151 MHz, CDCl$_3$)** δ = 142.90, 139.60, 137.92, 133.77, 129.06, 128.96 126.53, 125.64, 118.81, 116.98, 112.04, 106.66; **IR (neat):** ν_{max} = 3327, 1475, 1439, 1366, 1326, 1229, 1217, 1078, 737 cm^{-1}; ***R$_f$*** = 0.59 (hex/EtOAc 1/2).

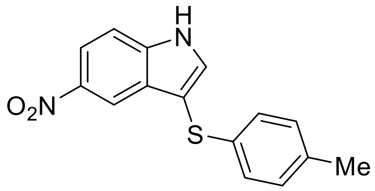

5-nitro-3-(p-tolylthio)-1H-indole, **3eb**[9]: yellow solid; **^{1}H NMR (600 MHz, DMSO-d_6)** δ 12.35 (s, 1H), 8.24 (d, J = 2.2 Hz, 1H), 8.07 (dd, J = 8.9, 2.3 Hz, 1H), 8.05 (s, 1H), 7.67 (d, J = 8.09Hz, 1H), 7.04 (d, J = 8.2 Hz, 2H), 7.00 (d, J = 8.2 Hz, 2H), 2.19 (s, 3H); **^{13}C NMR (151 MHz, DMSO-d_6)** δ = 141.51, 139.96, 136.17, 134.87, 134.23, 129.74, 128.12, 126.25, 117.53, 114.84, 113.15, 103.51, 20.39; **IR (neat):** ν_{max} = 3418, 1049, 1023, 1001, 823, 761, 515 cm^{-1}; ***R$_f$*** = 0.59 (hex/EtOAc 1/2).

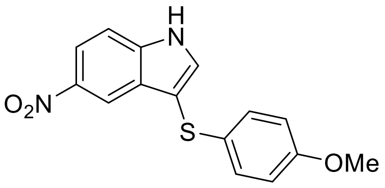

3-((4-methoxyphenyl)thio)-5-nitro-1H-indole, **3ec**[5]: yellow solid; **^{1}H NMR (600 MHz, DMSO-*d_6*) δ** 12.28 (s, 1H), 8.29 (d, *J* = 2.3 Hz, 1H), 8.05 (dd, *J* = 9.0, 2.3 Hz, 1H), 8.03 (s, 1H), 7.65 (d, *J* = 9.0 Hz, 1H), 7.13 (d, *J* = 8.8 Hz, 2H), 6.83 (d, *J* = 8.8 Hz, 2H), 3.67 (s, 3H); **^{13}C NMR (151 MHz, DMSO-*d_6*)** δ = 157.91, 141.52, 139.93, 135.71, 128.97, 128.10, 127.96, 117.53, 114.96, 114.91, 113.13, 105.01, 55.21; **IR (neat):** ν_{max} = 3422, 1050, 1023, 1002, 823, 760, 672 cm^{-1}; ***R_f*** = 0.53 (hex/EtOAc 1/2).

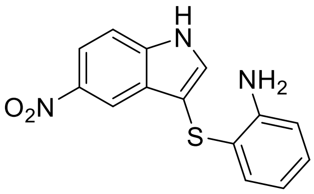

2-((5-nitro-1H-indol-3-yl)thio)aniline, **3ed**: yellow solid; m.p. 209-211 ^{o}C; **^{1}H NMR (600 MHz, DMSO-*d_6*)** δ 12.17 (s, 1H), 8.44 (d, *J* = 2.3 Hz, 1H), 8.03 (dd, *J* = 8.9, 2.3 Hz, 1H), 8.02 (s, 1H), 7.60 (d, *J* = 8.9 Hz, 1H), 7.10 (dd, *J* = 7.8, 1.4 Hz, 1H), 6.93 (ddd, *J* = 8.1, 1.4, 1.1 Hz, 1H), 6.70 (dd, *J* = 8.1, 1.1 Hz, 1H), 6.45 (ddd, *J* = 7.8, 1.4, 1.1 Hz, 1H), 5.42 (s, 2H); **^{13}C NMR (151 MHz, DMSO-*d_6*)** δ = 147.75, 141.30, 139.73, 134.69, 132.15, 128.44, 127.96, 117.51, 117.30, 116.69, 115.33, 114.80, 112.86, 105.45; **IR (neat):** ν_{max} = 2970, 1740, 1457, 1437, 1366, 1229, 1217 cm^{-1}; **HRMS** m/z (EI) calc. for **$C_{14}H_{11}N_3O_2S$** [M+] 285.0572, found 285.0574; ***R_f*** = 0.43 (hex/EtOAc 1/2).

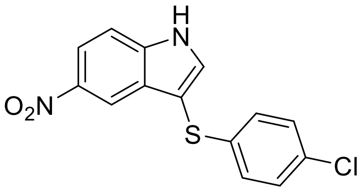

3-((4-chlorophenyl)thio)-5-nitro-1H-indole, **3ee**: yellow solid; m.p. 206-208 ^{o}C; **^{1}H NMR (600 MHz, DMSO-*d_6*)** δ 12.44 (s, 1H), 8.24 (d, *J* = 2.2 Hz, 1H), 8.10 (s, 1H), 8.08 (dd, *J* = 8.9, 2.2 Hz, 1H), 7.69 (d, *J* = 8.9 Hz, 1H), 7.27 (d, *J* = 8.6 Hz, 2H), 7.06 (d, *J* = 8.6 Hz, 2H); **^{13}C NMR (151 MHz, DMSO-*d_6*)** δ = 142.11, 140.45, 137.64, 137.18, 130.39, 129.44, 128.41, 127.74, 118.10, 115.13, 113.71, 102.59; **IR (neat):** ν_{max} = 3418, 1217, 1049, 1024, 1001, 823, 761, 672, 527 cm^{-1}; **HRMS** m/z (EI) calc. for **$C_{14}H_9ClN_2O_2S$** [M+] 304.0073, found 304.0075; ***R_f*** = 0.37 (hex/EtOAc 1/1).***R_f*** = 0.38 (hex/EtOAc 1/1).

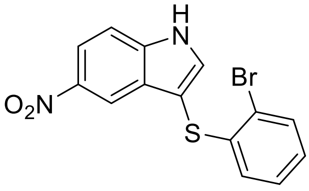

3-((2-bromophenyl)thio)-5-nitro-1H-indole, **3ef**: yellow solid; m.p. 193-195 ^{o}C; **^{1}H NMR (600 MHz, DMSO-*d_6*)** δ 8.21 (s, 1H), 8.17 (d, *J* = 8.1 Hz, 1H), 8.1 (d, *J* = 8.1 Hz, 1H), 7.7 (d, *J* = 7.7 Hz, 1H), 7.60 (d, *J* = 7.7 Hz, 1H), 7.11 (d, *J* = 8.5 Hz, 1H), 7.02 (d, *J* = 8.5Hz, 1H), 6.53 (s, 1H); **^{13}C NMR (151 MHz, DMSO-*d_6*)** δ = 141.77, 140.18, 139.18, 137.36, 132.65,

128.27, 128.04, 126.64, 126.07, 119.03, 117.77, 114.61, 113.43, 101.39; **IR (neat):** ν_{max} = 3394, 1217, 1023, 995, 824, 762, 672, 527 cm^{-1}; **HRMS** m/z (EI) calc. for $\mathbf{C_{14}H_9BrN_2O_2S}$ [M+] 349.9548, found 349.9539; $\boldsymbol{R_f}$ = 0.36 (hex/EtOAc 1/1).

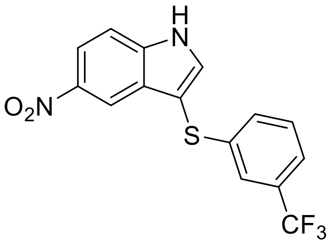

5-nitro-3-((3-(trifluoromethyl)phenyl)thio)-1H-indole, **3eg**: yellow solid; m.p. 166-168 °C; **^{1}H NMR (600 MHz, DMSO-*d$_6$*)** δ 8.26 (d, *J* = 2.2 Hz, 1H), 8.15 (s, 1H), 8.08 (dd, *J* = 9.0, 2.2 Hz, 1H), 7.71 (d, *J* = 9.0 Hz, 1H), 7.44 (d, *J* = 4.0 Hz, 1H), 7.43 (d, *J* = 3.6 Hz, 1H), 7.39 (s, 1H), 7.28 (dd, *J* = 4.0, 3.6 Hz, 1H)**; ^{13}C NMR (151 MHz, DMSO-*d$_6$*)** δ = 141.78, 140.27, 140.05, 137.15, 130.16, 129.86 (q, $^2J_{C-F}$ = 31.8 Hz), 129.28, 127.94, 123.79 (q, $^1J_{C-F}$ = 272.9 Hz), 121.96 (q, $^3J_{C-F}$ = 3.8 Hz), 121.58 (q, $^3J_{C-F}$ = 3.8 Hz), 117.75, 114.61, 113.38, 101.22; **^{19}F NMR (564 MHz, DMSO-*d$_6$*)** δ = –61.5435; **IR (neat):** ν_{max} = 3418, 1738, 1217, 1024, 1003, 823, 760, 527 cm^{-1}; **HRMS** m/z (EI) calc. for $\mathbf{C_{15}H_9F_3N_2O_2S}$ [M+] 338.0337, found 338.0334; $\boldsymbol{R_f}$ = 0.40 (hex/EtOAc 1/1).

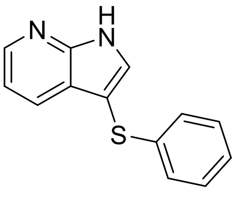

3-(phenylthio)-1H-pyrrolo[2,3-b]pyridine, **9a**[10]: white solid; **^{1}H NMR (600 MHz, DMSO-*d$_6$*)** δ 12.26 (s, 1H), 8.30 (dd, *J* = 4.6, 1.5 Hz, 1H), 7.92 (s, 1H), 7.78 (dd, *J* = 7.5, 1.5 Hz, 1H), 7.21 (dd, *J* = 7.9, 7.5 Hz, 2H), 7.13 (ddd, *J* = 7.8, 7.4, 4.6 Hz, 1H), 7.08 (dd, *J* = 7.8, 7.4 Hz, 1H), 7.04 (d, *J* = 7.9, 1.5 Hz, 2H); **^{13}C NMR (151 MHz, DMSO-*d$_6$*) δ** = 149.34, 144.13, 138.99, 133.63, 129.41, 127.13, 126.00, 125.54, 121.40, 117.04, 99.10; **IR (neat):** ν_{max} = 3423, 1050, 1024, 1003, 823, 760, 623 cm^{-1}; $\boldsymbol{R_f}$ = 0.38 (hex/EtOAc 1/2).

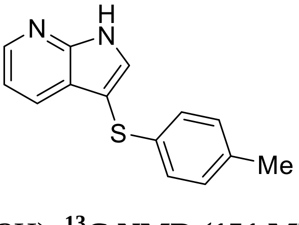

3-(p-tolylthio)-1H-pyrrolo[2,3-b]pyridine, **9b**[10]: white solid; **^{1}H NMR (600 MHz, CDCl$_3$)** δ 12.25 (s, 1H), 8.44 – 8.39 (dd, *J* = 4.8, 1.2 Hz, 1H), 7.97 (dd, *J* = 7.8, 1.2 Hz, 1H), 7.71 (s, 1H), 7.16 (dd, *J* = 7.8, 4.8 Hz, 1H), 7.05 (d, *J* = 8.2 Hz, 2H), 7.00 (d, *J* = 8.2 Hz, 2H), 2.26 (s, 3H); **^{13}C NMR (151 MHz, CDCl$_3$)** δ = 149.29, 143.11, 135.05, 134.98, 131.68, 129.54, 128.50, 126.48, 122.22, 116.67, 102.00, 20.85; **IR (neat):** ν_{max} = 3132, 3078, 3020, 2349, 1491, 1411, 1282, 1217, 803, 770, 672, 665 cm^{-1}; $\boldsymbol{R_f}$ = 0.39 (hex/EtOAc 1/2).

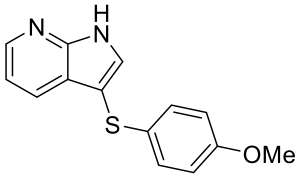

3-((4-methoxyphenyl)thio)-1H-pyrrolo[2,3-b]pyridine, **9c**[5]: white solid; **^{1}H NMR (600 MHz, $CDCl_3$)** δ 11.81 (s, 1H), 8.39 (s, 1H), 7.96 (d, *J* = 7.7 Hz, 1H), 7.66 (s, 1H), 7.15 (d, *J* = 8.8 Hz, 2H), 7.14(d, *J* = 7.7 Hz, 1H), 6.75 (d, *J* = 8.8 Hz, 2H), 3.74 (s, 3H); **^{13}C NMR (151 MHz, $CDCl_3$)** δ = 158.23, 149.33, 143.34, 131.27, 129.14, 128.64, 122.30, 116.93, 114.76, 103.52, 101.01, 55.54; **IR (neat):** ν_{max} = 3418, 1660, 1050, 1023, 1002, 823, 761, 526 cm^{-1}; ***R_f*** = 0.34 (hex/EtOAc 1/2).

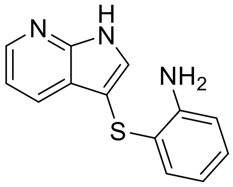

2-((1H-pyrrolo[2,3-b]pyridin-3-yl)thio)aniline, **9d**: brown solid; m.p. 128-130 °C; **^{1}H NMR (600 MHz, DMSO-*d_6*)** δ 12.04 (s, 1H), 8.25 (s, 1H), 7.91 (dd, *J* = 8.2, 7.6 Hz, 1H), 7.90 (d, *J* = 7.6 Hz, 1H), 7.13 (d, *J* = 7.9 Hz, 1H), 7.09 (d, *J* = 7.4 Hz, 1H), 6.92 (dd, *J* = 7.9, 7.7 Hz, 1H), 6.67 (d, *J* = 8.2 Hz, 1H), 6.43 (dd, *J* = 7.7, 7.4 Hz, 1H), 5.37 (s, 2H); **^{13}C NMR (151 MHz, DMSO-*d_6*)** δ = 148.66, 147.67, 143.34, 132.28, 131.52, 128.25, 126.86, 120.92, 118.02, 116.54, 116.17, 114.62, 101.20; **IR (neat):** ν_{max} = 2970, 1738, 1478, 1460, 1412, 1366, 1229, 1217, 750 cm^{-1}; **HRMS** m/z (EI) calc. for **$C_{13}H_{11}N_3S$** [M+] 241.0674, found 241.0675; ***R_f*** = 0.24 (hex/EtOAc 1/2).

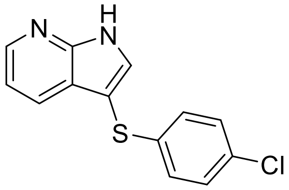

3-((4-chlorophenyl)thio)-1H-pyrrolo[2,3-b]pyridine, **9e**[10]: white solid; **^{1}H NMR (600 MHz, DMSO-*d_6*)** δ 12.39 (s, 1H), 8.33 (d, *J* = 4.8 Hz, 1H), 7.96 (s, 1H), 7.81 (d, *J* = 7.9 Hz, 1H), 7.27 (d, *J* = 8.7 Hz, 2H), 7.16 (dd, *J* = 7.9, 4.8 Hz, 1H), 7.04 (d, *J* = 8.7 Hz, 2H); **^{13}C NMR (151 MHz, DMSO-*d_6*)** δ = 148.51, 143.42, 137.72, 133.58, 129.65, 128.89, 127.14, 127.01, 120.97, 116.70, 98.20; **IR (neat):** ν_{max} = 2970, 1738, 1474, 1366, 1229, 1217, 770 cm^{-1}; ***R_f*** = 0.43 (hex/EtOAc 1/2).

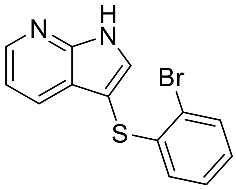

3-((2-bromophenyl)thio)-1H-pyrrolo[2,3-b]pyridine, **9f**: white solid; m.p. 195-197 °C; **^{1}H NMR (600 MHz, DMSO-*d_6*)** δ 12.41 (s, 1H), 8.34 (dd, *J* = 4.7, 1.6 Hz, 1H), 7.98 (s, 1H), 7.78 (dd, *J* = 7.9, 1.5 Hz, 1H), 7.58 (dd, *J* = 7.8, 1.3 Hz, 1H), 7.15 (dd, *J* = 7.8, 4.7 Hz, 1H), 7.11 (dd, *J* = 7.7, 1.3 Hz, 1H), 7.01 (dd, *J* = 7.7, 1.6 Hz, 1H), 6.52 (dd, *J* = 7.9, 1.5 Hz, 1H); **^{13}C NMR (151 MHz, DMSO-*d_6*)** δ = 149.04, 143.93, 139.76, 134.07, 132.59, 128.14, 126.66, 126.38, 125.96, 120.83, 118.83, 116.85, 97.34; **IR (neat):** ν_{max} = 3396, 1738, 1366, 1229, 1217, 1023, 999, 761, 672

cm^{-1}; **HRMS** m/z (EI) calc. for **$C_{13}H_9BrN_2S$** [M+] 305.9649, found 305.9648; ***R_f*** = 0.35 (hex/EtOAc 1/2).

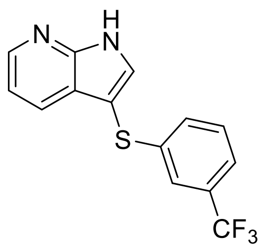

3-((3-(trifluoromethyl)phenyl)thio)-1H-pyrrolo[2,3-b]pyridine, **9g**[11]: white solid; **^{1}H NMR (600 MHz, $CDCl_3$)** δ 13.06 (s, 1H), 8.47 (d, *J* = 4.3 Hz, 1H), 7.97 (d, *J* = 7.9 Hz, 1H), 7.79 (s, 1H), 7.41 (s, 1H), 7.33 (d, *J* = 7.6 Hz, 1H), 7.27 (dd, *J* = 7.6, 7.1 Hz, 1H), 7.22 (d, *J* = 7.1 Hz, 1H), 7.20 (dd, *J* = 7.9, 4.3 Hz, 1H)**; ^{13}C NMR (151 MHz, $CDCl_3$)** δ = 149.62, 143.41, 140.89, 132.87, 131.21 (q, $^2J_{C–F}$ = 32.3 Hz), 129.33, 129.05, 128.65, 123.82 (q, $^1J_{C–F}$ = 272.9 Hz), 122.39 (q, $^3J_{C–F}$ = 3.5 Hz), 122.31, 121.74 (q, $^3J_{C–F}$ = 3.7 Hz), 117.15, 100.00; **^{19}F NMR (564 MHz, $CDCl_3$)** δ = –62.8026; **IR (neat):** ν_{max} = 3079, 2920, 2869, 1412, 1321, 1125, 771, 696 cm^{-1}; ***R_f*** = 0.47 (hex/EtOAc 1/2).

References

[1] S.-L. Yi, M.-C. Li, X.-Q. Hu, W.-M. Mo, Z.-L. Shen, *Chin. Chem. Lett.* **2016**, 27, 1505.

[2] C.-R. Liu, L.-H. Ding, *Org. Biomol. Chem.* **2015**, 13, 2251.

[3] A. K. Pandey, S. Chand, R. Singh, S. Kumar, K. N. Singh, *ACS omega* **2020**, 5, 7627.

[4] R. Ohkado, T. Ishikawa, H. Iida, *Green Chem.* **2018**, 20, 984.

[5] Y. Yang, S. Zhang, L. Tang, Y. Hu, Z. Zha, Z. Wang, *Green Chem.* **2016**, 18, 2609.

[6] C. D. Prasad, S. Kumar, M. Sattar, A. Adhikary, S. Kumar, *Org. Biomol. Chem.* **2013**, 11, 8036.

[7] S. Song, Y. Zhang, A. Yeerlan, B. Zhu, J. Liu, N. Jiao, *Angew. Chem. Int. Ed.* **2017**, 56, 2487.

[8] P. Sang, Z. Chen, J. Zou, Y. Zhang, *Green Chem.* **2013**, 15, 2096.

[9] X. Yang, Y. Bao, Z. Dai, Q. Zhou, F. Yang, *Green Chem.* **2018**, 20, 3727.

[10] S. Liu, H. Yang, L.-Y. Jiao, J.-H. Zhang, C. Zhao, Y. Ma, X. Yang, *Org. Biomol. Chem.* **2019**, 17, 10073.

[11] R. C. Bernotas, S. Lenicek, S. Antane, D. C. Cole, B. L. Harrison, A. J. Robichaud, G. M. Zhang, D. Smith, B. Platt, Q. Lin, P. Li, J. Coupet, S. R.-Lipson, C. E. Beyer, L. E. Schechter, *Bioorg. Med. Chem.* **2009**, 17, 5153.

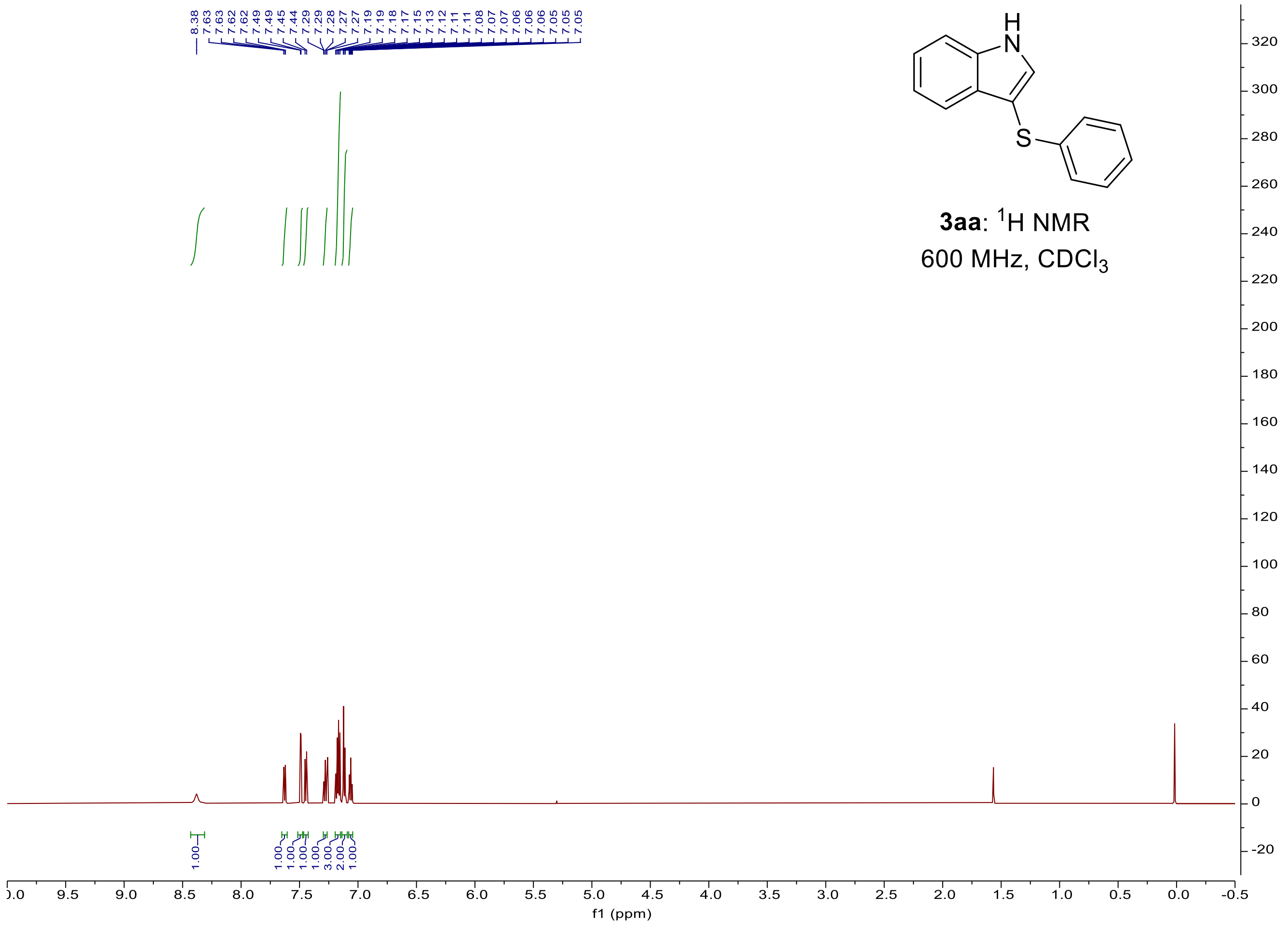

3aa: 1H NMR
600 MHz, CDCl3
8.38
7.63
7.63
7.62
7.62
7.49
7.49
7.45
7.44
7.29
7.29
7.28
7.27
7.27
7.19
7.19
7.18
7.17
7.15
7.13
7.12
7.11
7.11
7.08
7.07
7.07
7.06
7.06
7.05
7.05
7.05
1.00
1.00
1.00
1.00
1.00
3.00
2.00
1.00
f1 (ppm)

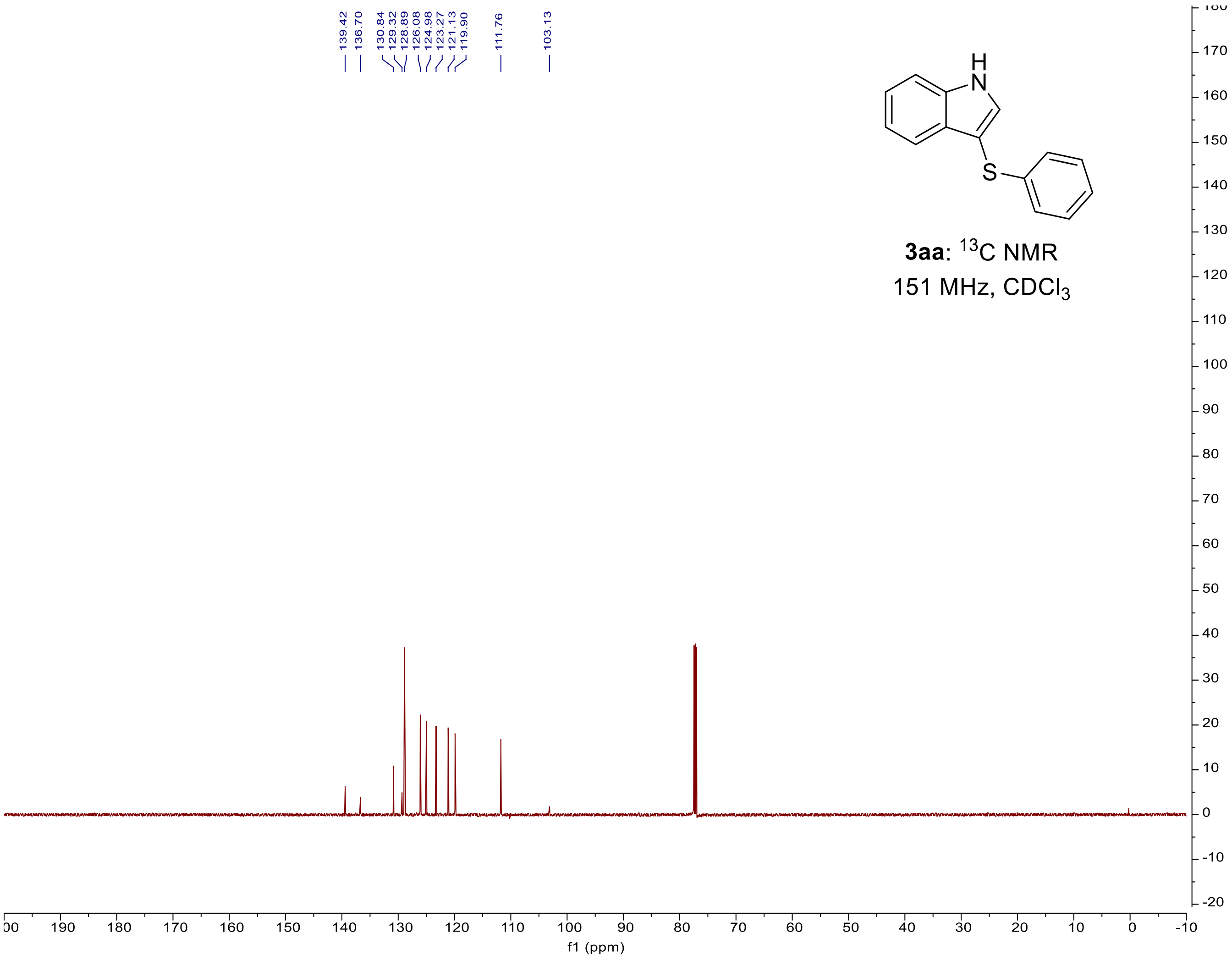
139.42
136.70
130.84
129.32
128.89
126.08
124.98
123.27
121.13
119.90
111.76
103.13
H
N
S
3aa: 13C NMR
151 MHz, CDCl3
f1 (ppm)

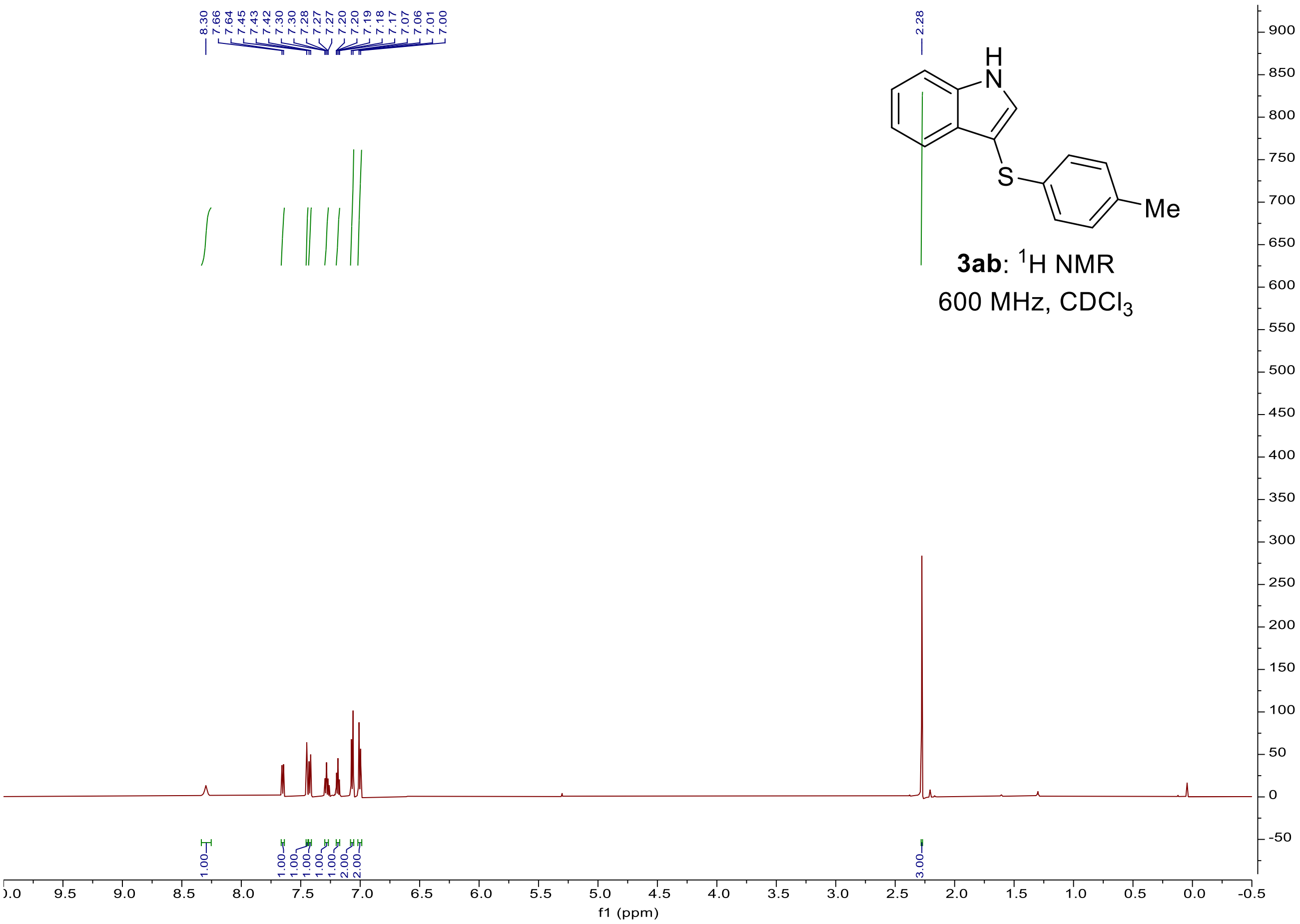
3ab: ^{1}H NMR
600 MHz, $CDCl_3$
Me
S
H
N
f1 (ppm)

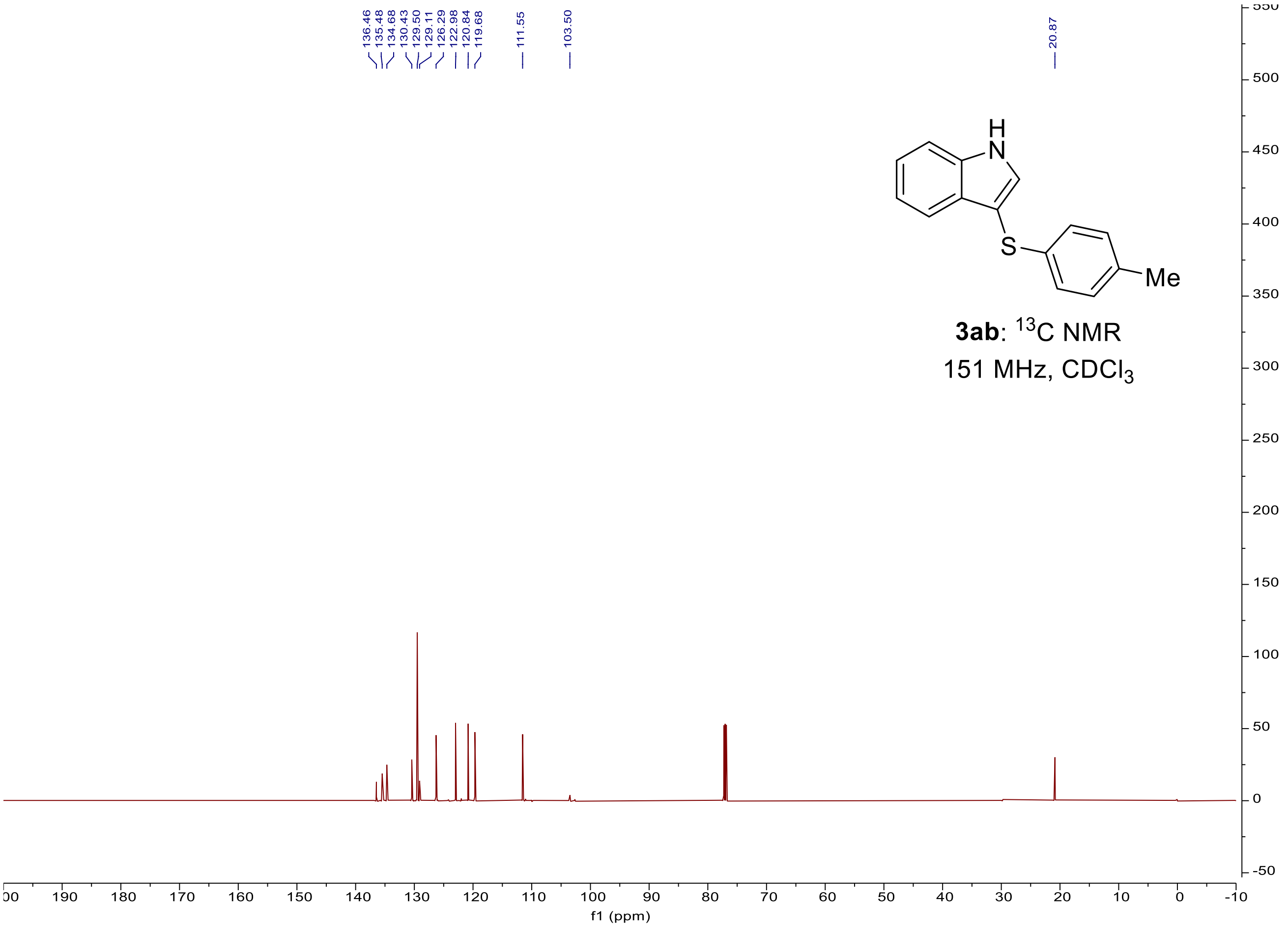
136.46
135.48
134.68
130.43
129.50
129.11
126.29
122.98
120.84
119.68
111.55
103.50
20.87
H
N
S
Me
3ab: ^{13}C NMR
151 MHz, $CDCl_3$
f1 (ppm)

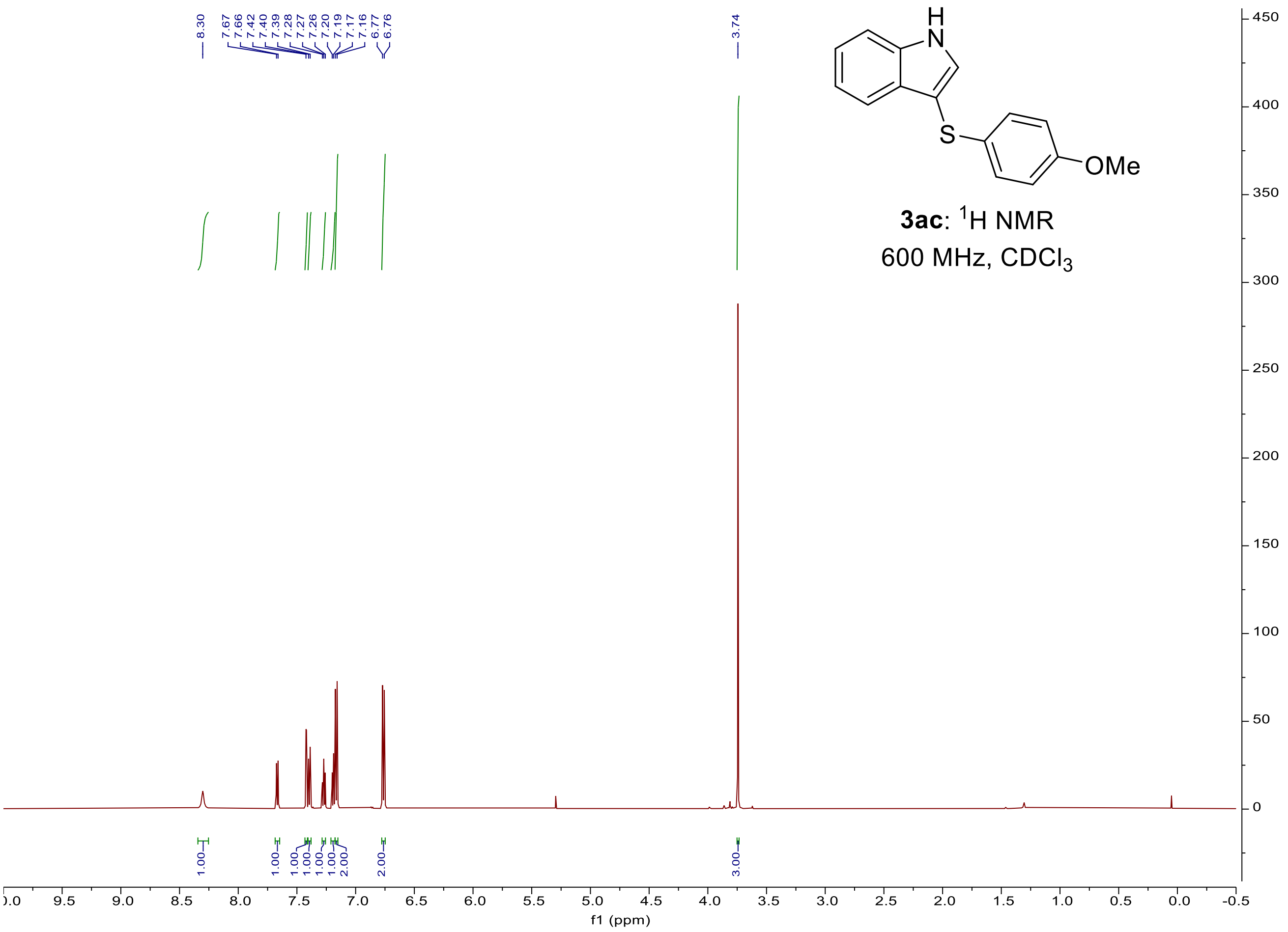
8.30
7.67
7.66
7.42
7.40
7.39
7.28
7.27
7.26
7.20
7.19
7.17
7.16
6.77
6.76
3.74
H
N
S
OMe
3ac: 1H NMR
600 MHz, CDCl3
1.00
1.00
1.00
1.00
1.00
1.00
2.00
2.00
3.00
f1 (ppm)

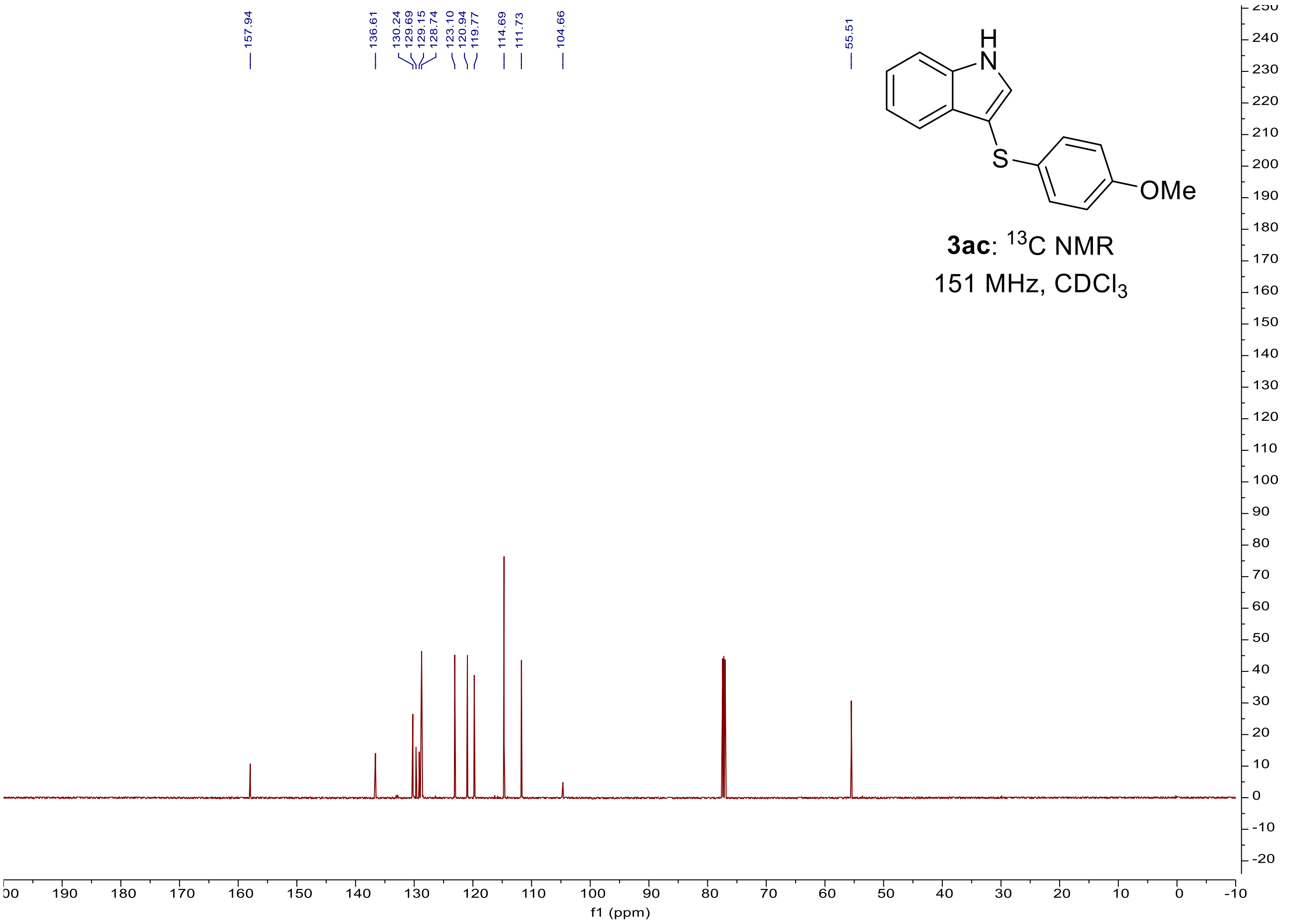
157.94
136.61
130.24
129.69
129.15
128.74
123.10
120.94
119.77
114.69
111.73
104.66
55.51
H
N
S
OMe
3ac: 13C NMR
151 MHz, CDCl3
f1 (ppm)

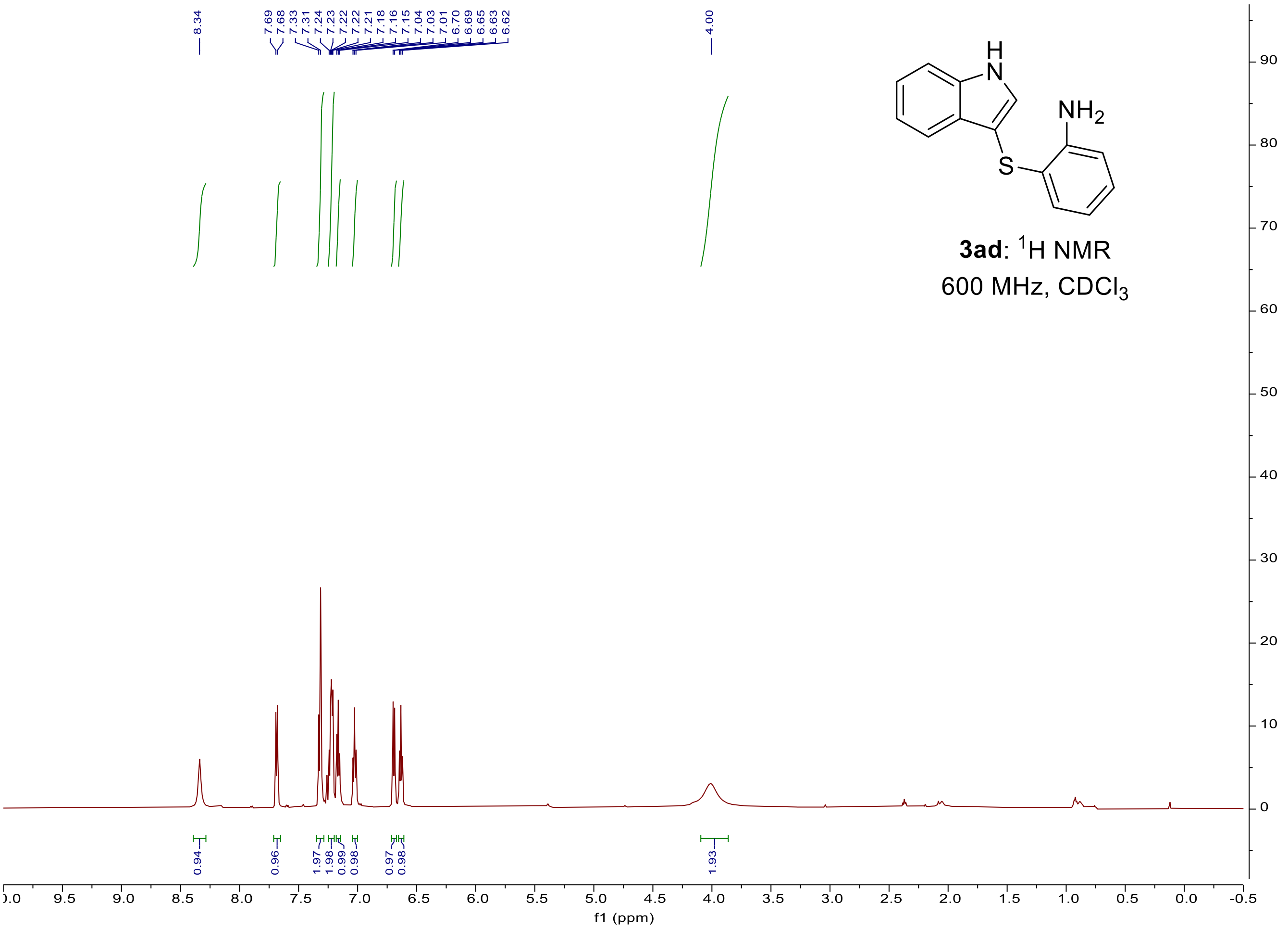
8.34
7.69
7.68
7.33
7.31
7.24
7.23
7.22
7.22
7.21
7.18
7.16
7.15
7.04
7.03
7.01
6.70
6.69
6.65
6.63
6.62
4.00
H
N
NH2
S
3ad: 1H NMR
600 MHz, CDCl3
0.94
0.96
1.97
1.98
0.99
0.98
0.97
0.98
1.93
f1 (ppm)

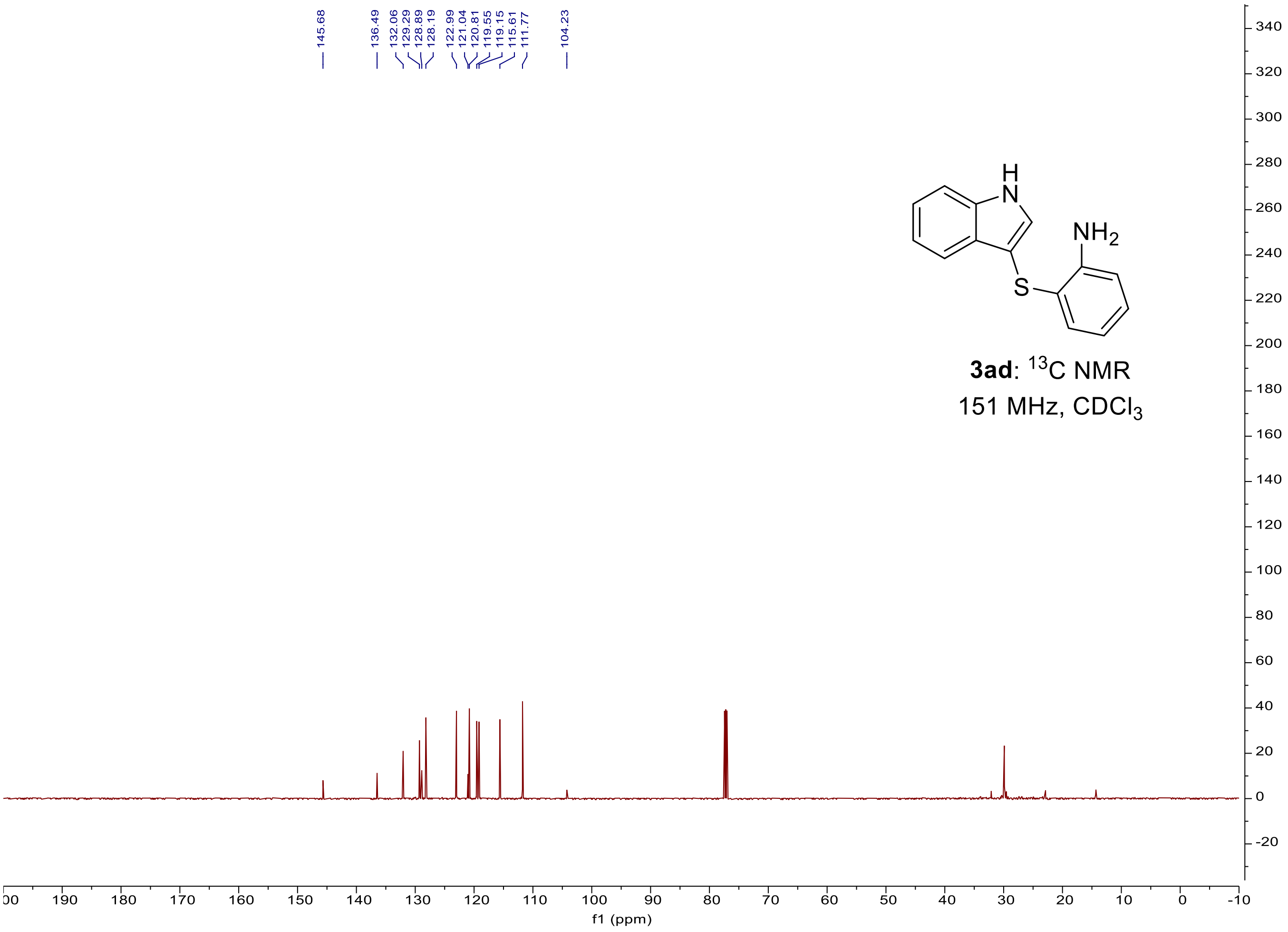
145.68
136.49
132.06
129.29
128.89
128.19
122.99
121.04
120.81
119.55
119.15
115.61
111.77
104.23
H
N
NH2
S
3ad: 13C NMR
151 MHz, CDCl3
f1 (ppm)

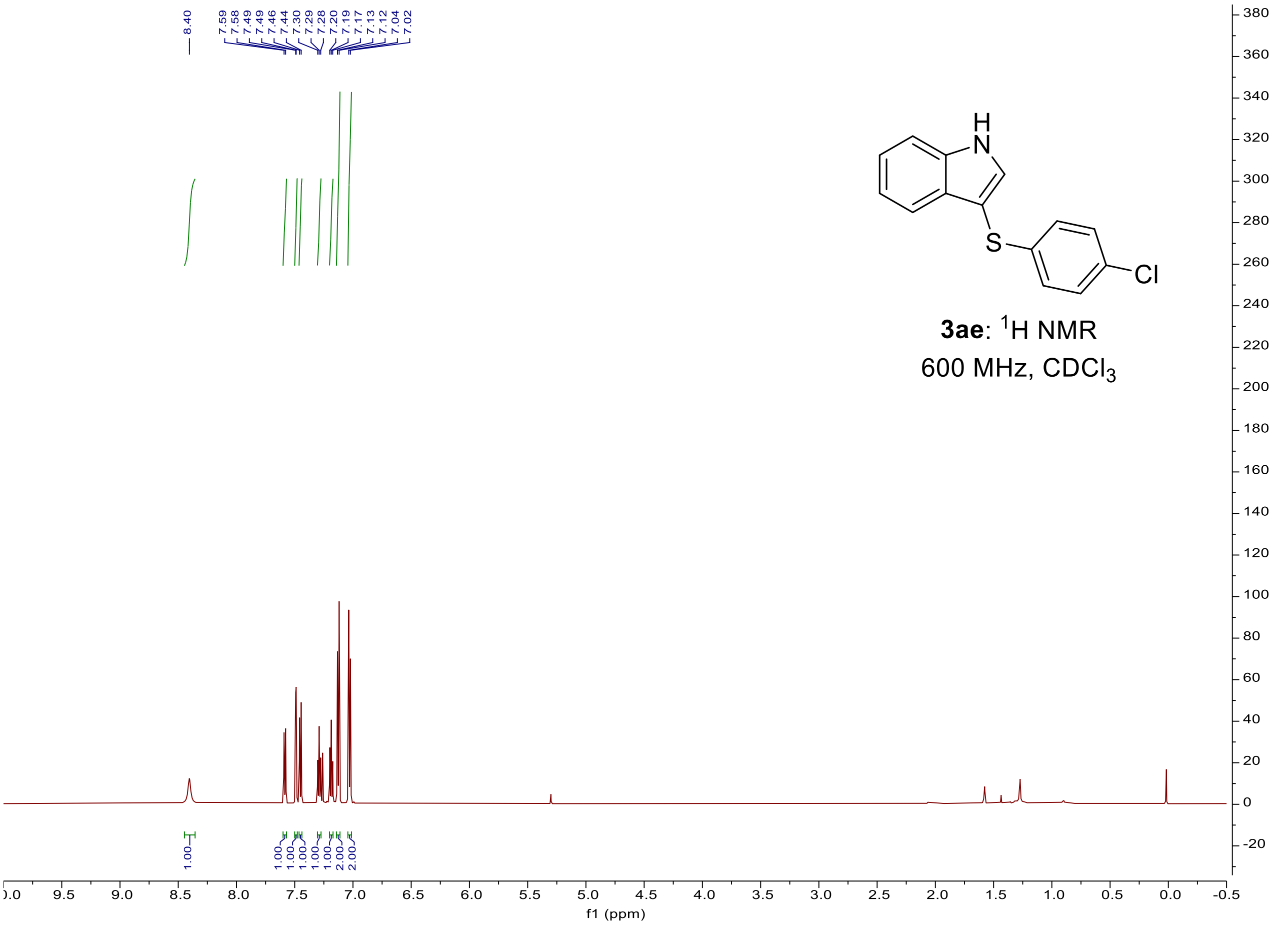
8.40
7.59
7.58
7.49
7.46
7.44
7.30
7.29
7.28
7.20
7.19
7.17
7.13
7.12
7.04
7.02
3ae: 1H NMR
600 MHz, CDCl3
1.00
1.00
1.00
1.00
1.00
1.00
2.00
2.00
f1 (ppm)

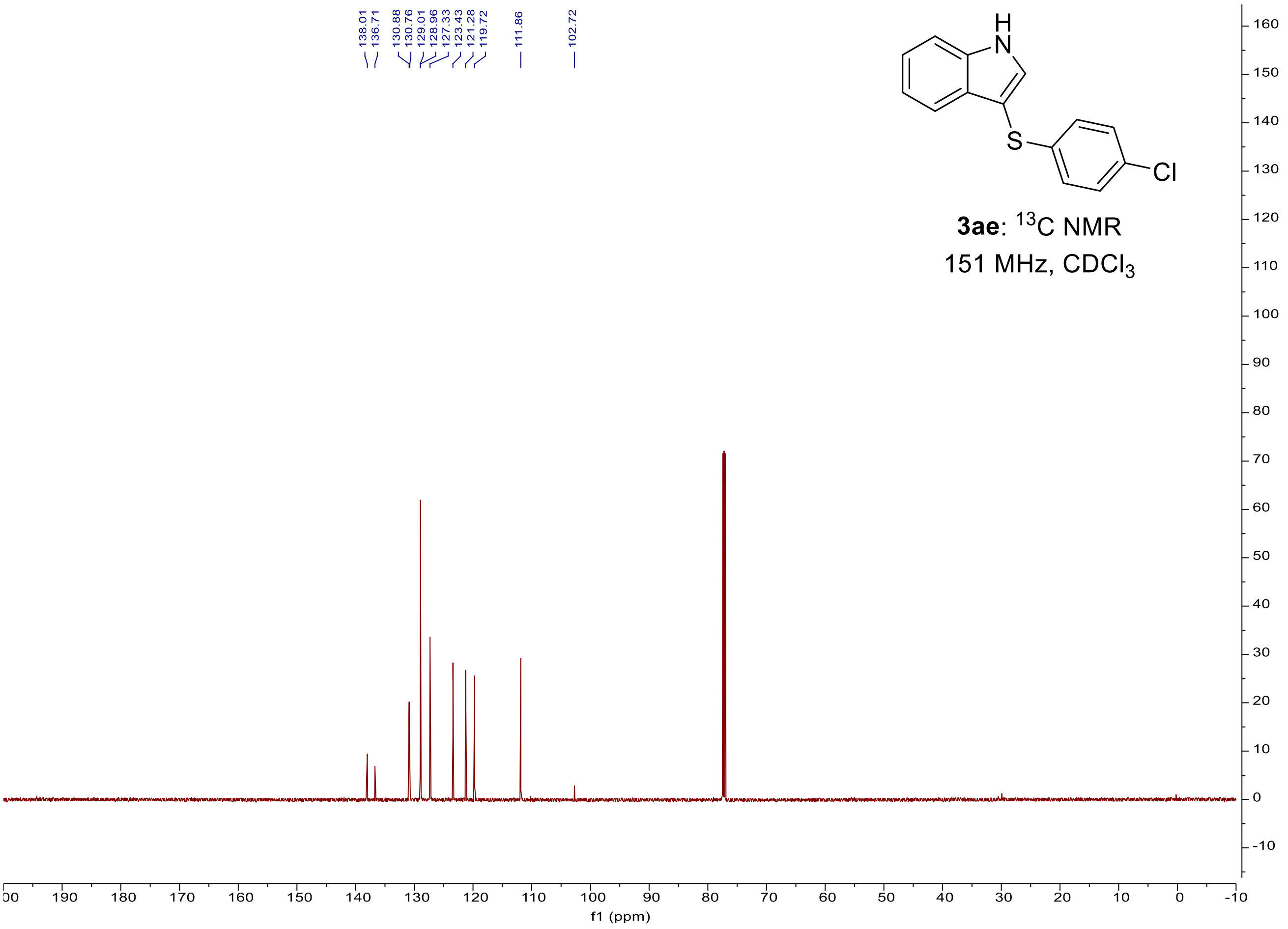
138.01
136.71
130.88
130.76
129.01
128.96
127.33
123.43
121.28
119.72
111.86
102.72
3ae: 13C NMR
151 MHz, CDCl3
f1 (ppm)

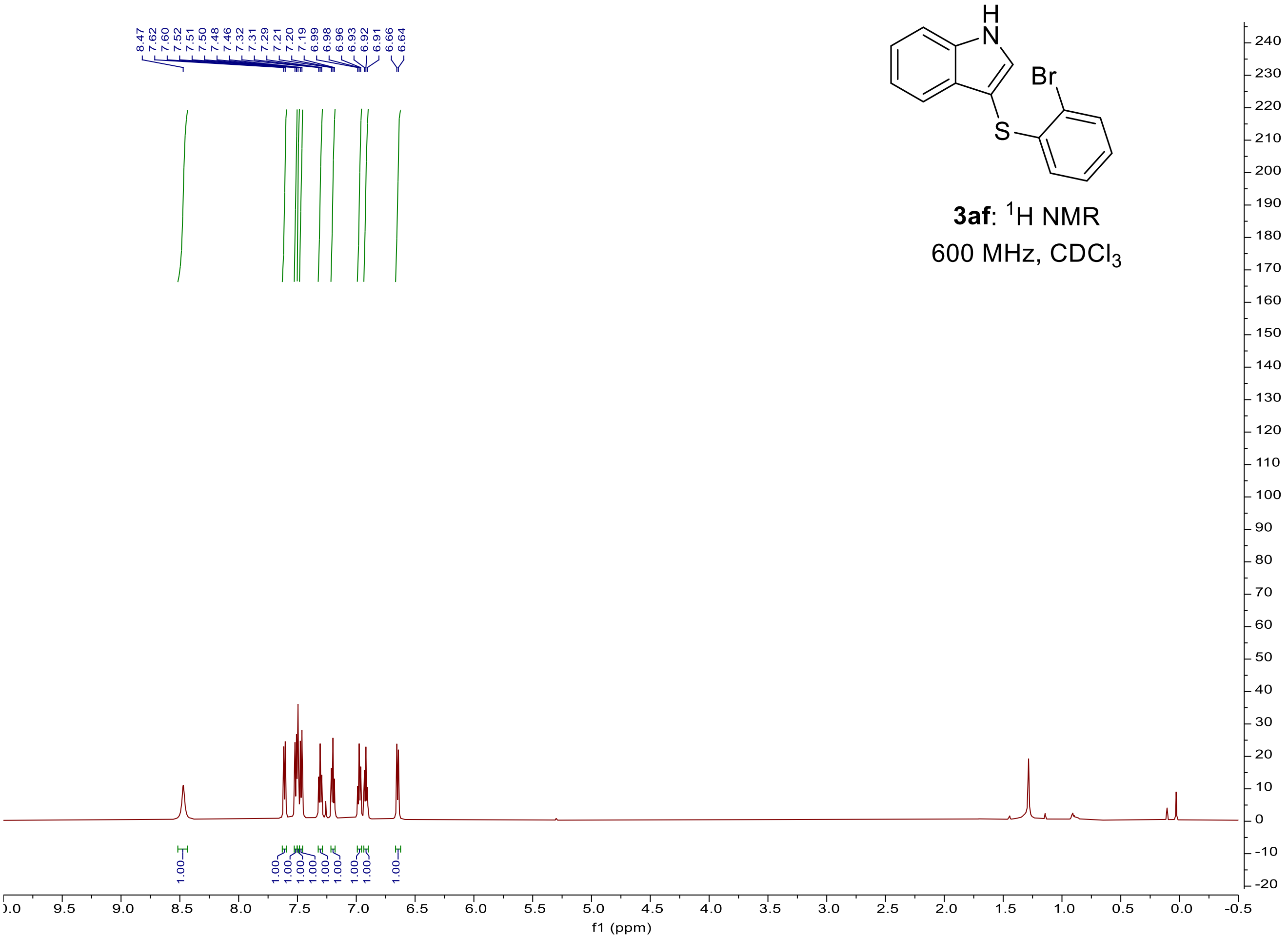

3af: ^{1}H NMR
600 MHz, $CDCl_3$

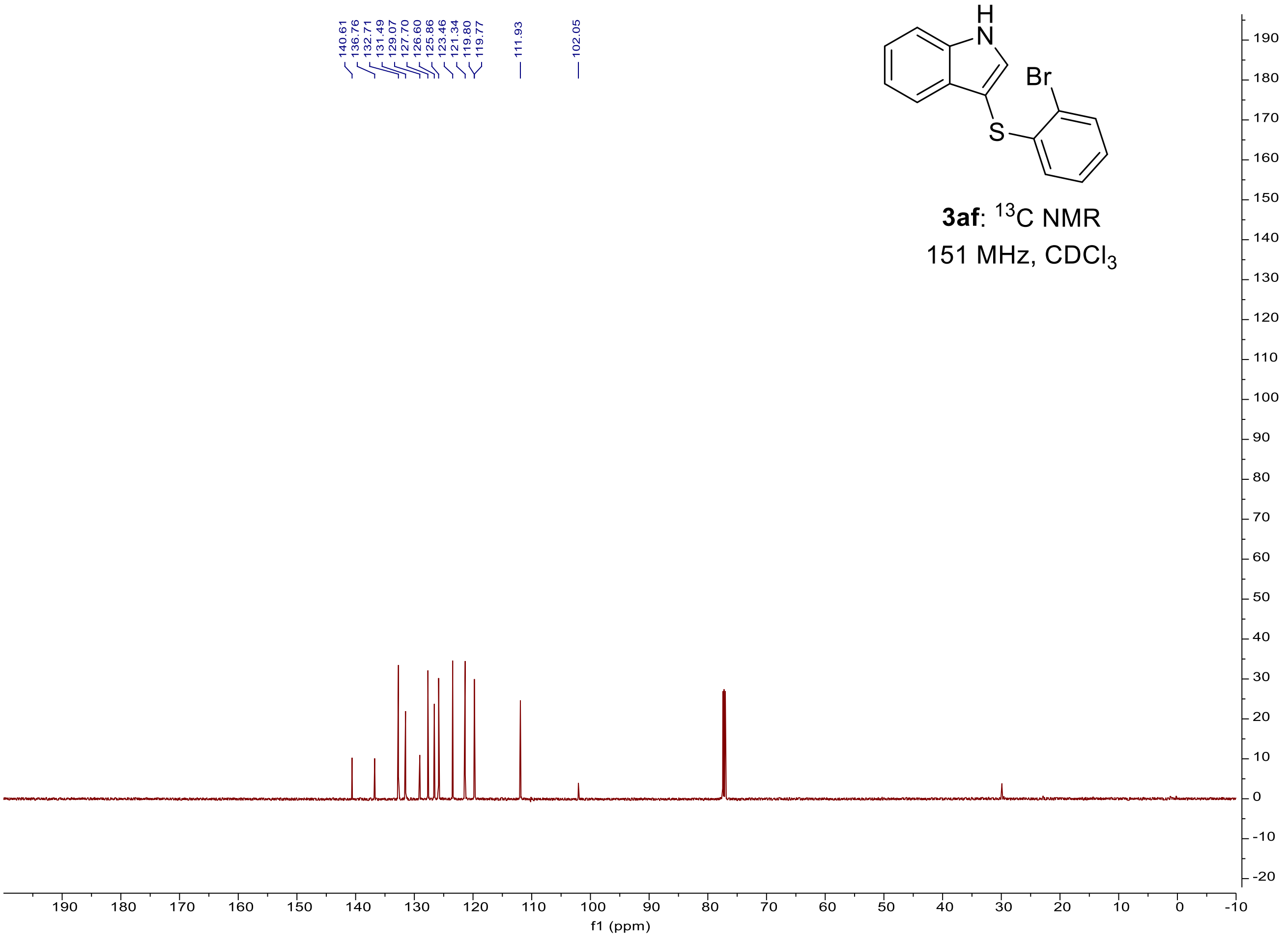
140.61
136.76
132.71
131.49
129.07
127.70
126.60
125.86
123.46
121.34
119.80
119.77
111.93
102.05
Br
S
H
N
3af: 13C NMR
151 MHz, CDCl3
f1 (ppm)

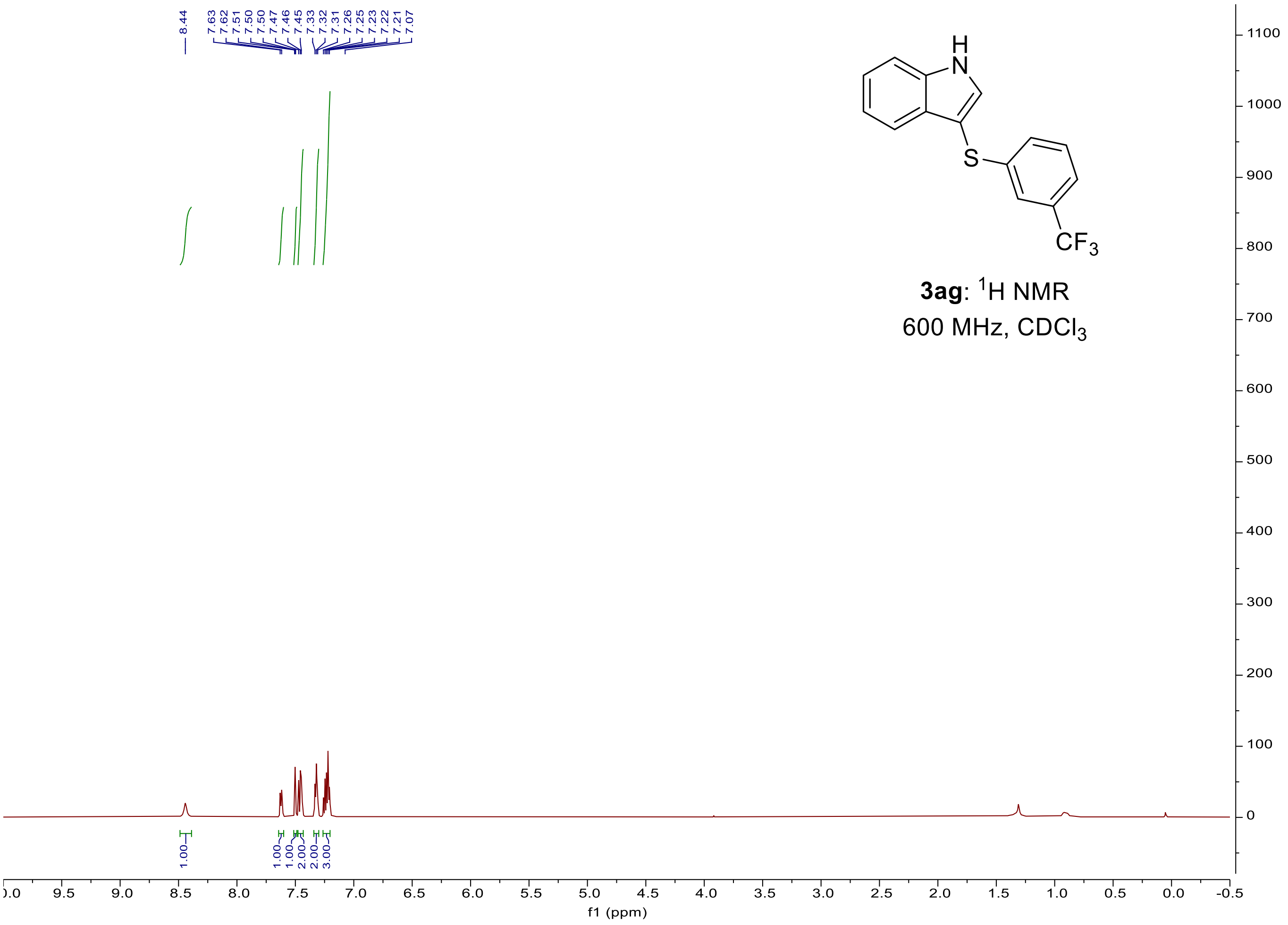
8.44
7.63
7.62
7.51
7.50
7.50
7.47
7.46
7.45
7.33
7.32
7.31
7.26
7.25
7.23
7.22
7.21
7.07
H
N
S
CF3
3ag: 1H NMR
600 MHz, CDCl3
1.00
1.00
1.00
2.00
2.00
3.00
f1 (ppm)

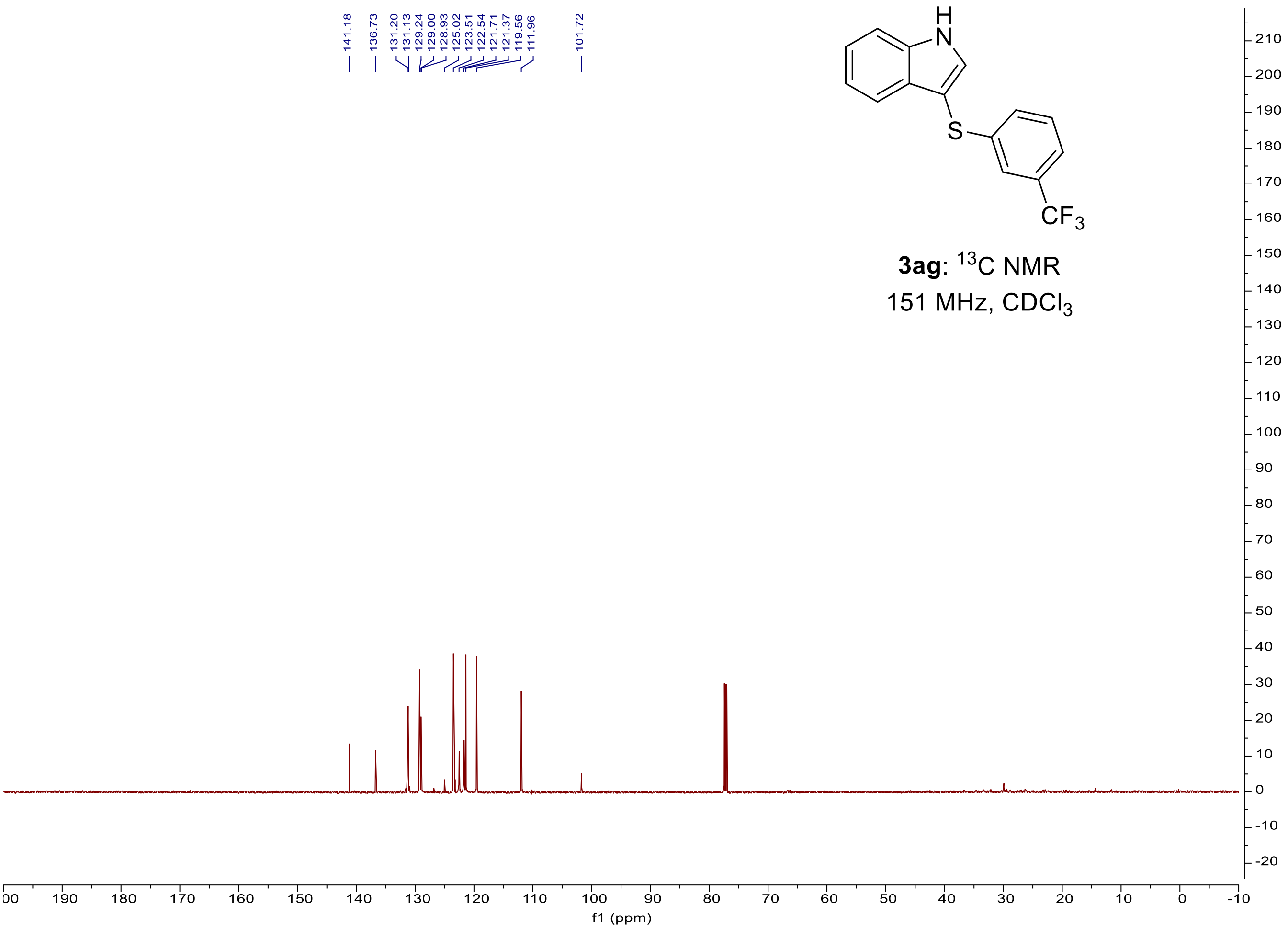
141.18
136.73
131.20
131.13
129.24
129.00
128.93
125.02
123.51
122.54
121.71
121.37
119.56
111.96
101.72
H
N
S
CF3
3ag: 13C NMR
151 MHz, CDCl3
f1 (ppm)

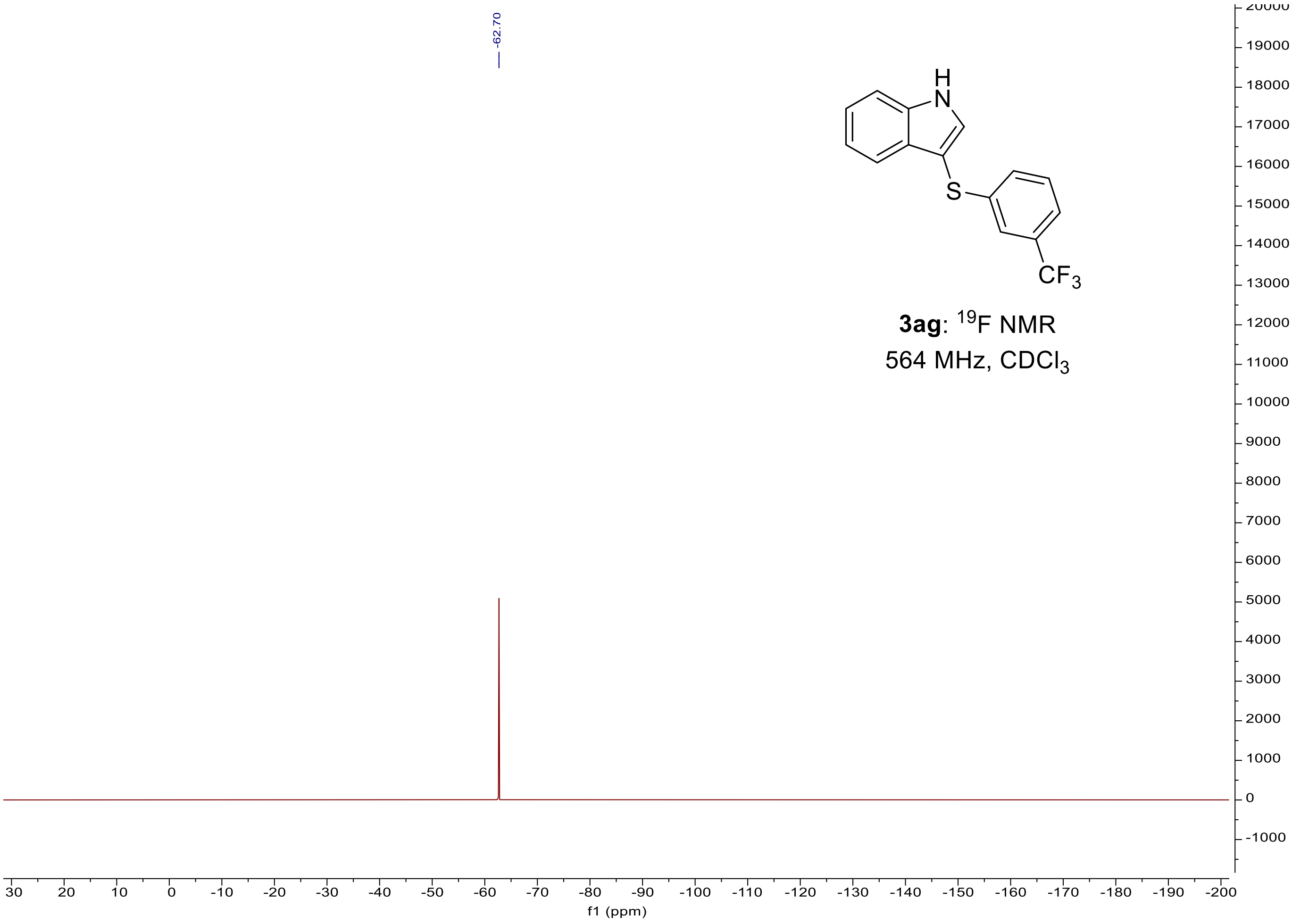

-62.70
H
N
S
CF3
3ag: 19F NMR
564 MHz, CDCl3
f1 (ppm)

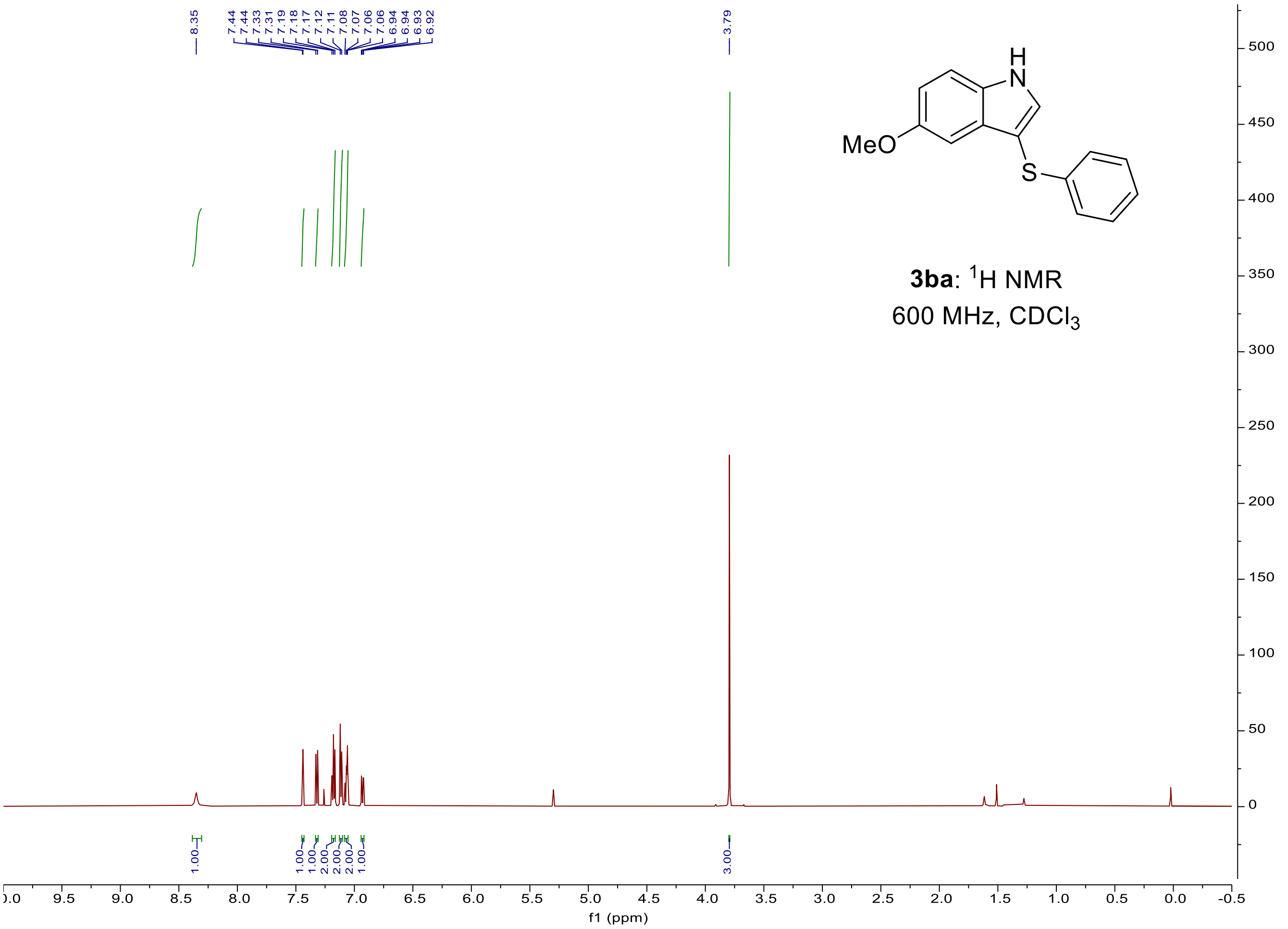
8.35
7.44
7.44
7.33
7.31
7.19
7.18
7.17
7.12
7.11
7.08
7.07
7.06
7.06
6.94
6.94
6.93
6.92
3.79
MeO
H
N
S
3ba: ^{1}H NMR
600 MHz, $CDCl_3$
1.00
1.00
1.00
2.00
2.00
2.00
1.00
3.00
f1 (ppm)

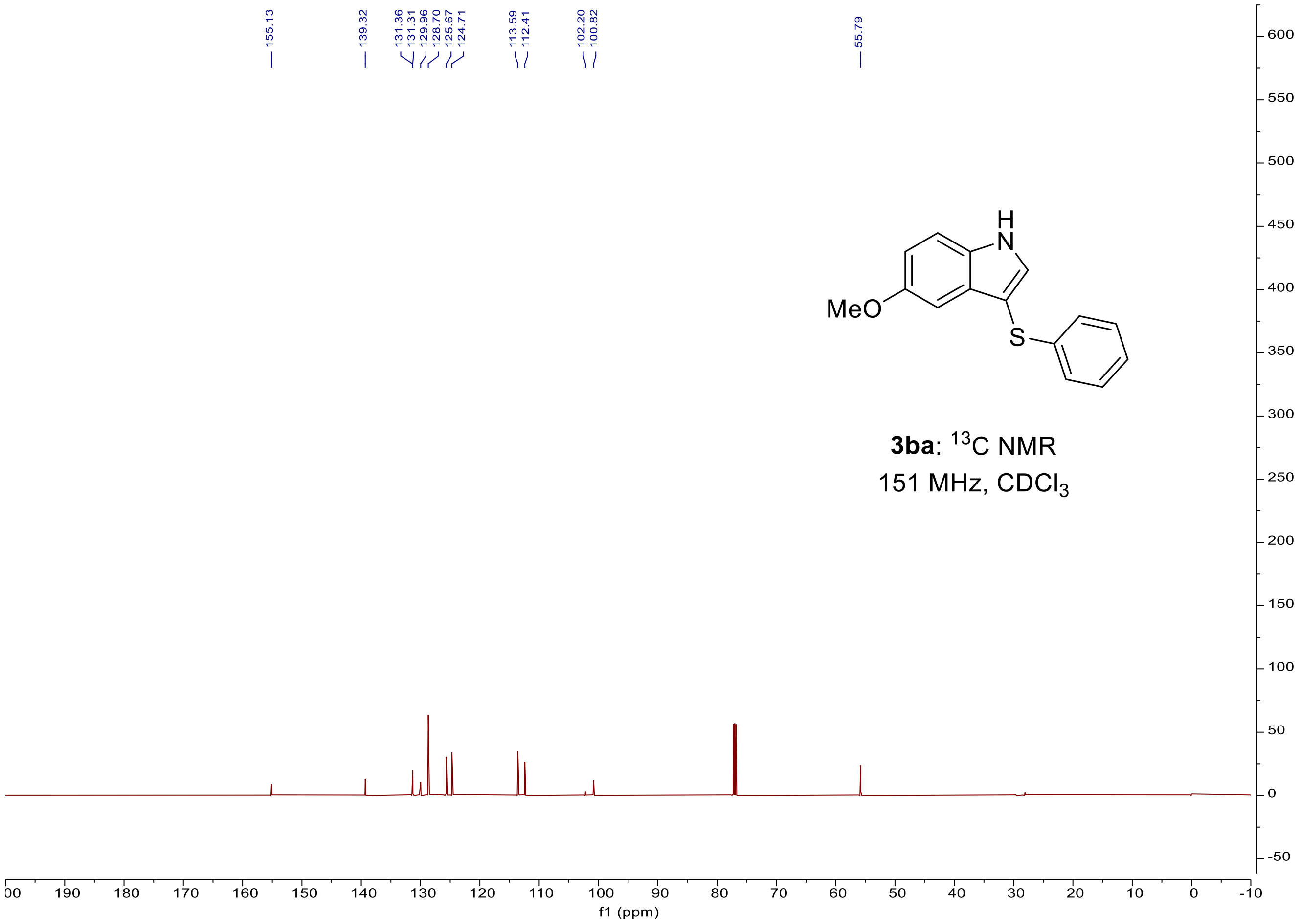
155.13
139.32
131.36
131.31
129.96
128.70
125.67
124.71
113.59
112.41
102.20
100.82
55.79
MeO
H
N
S
3ba: 13C NMR
151 MHz, CDCl3
f1 (ppm)

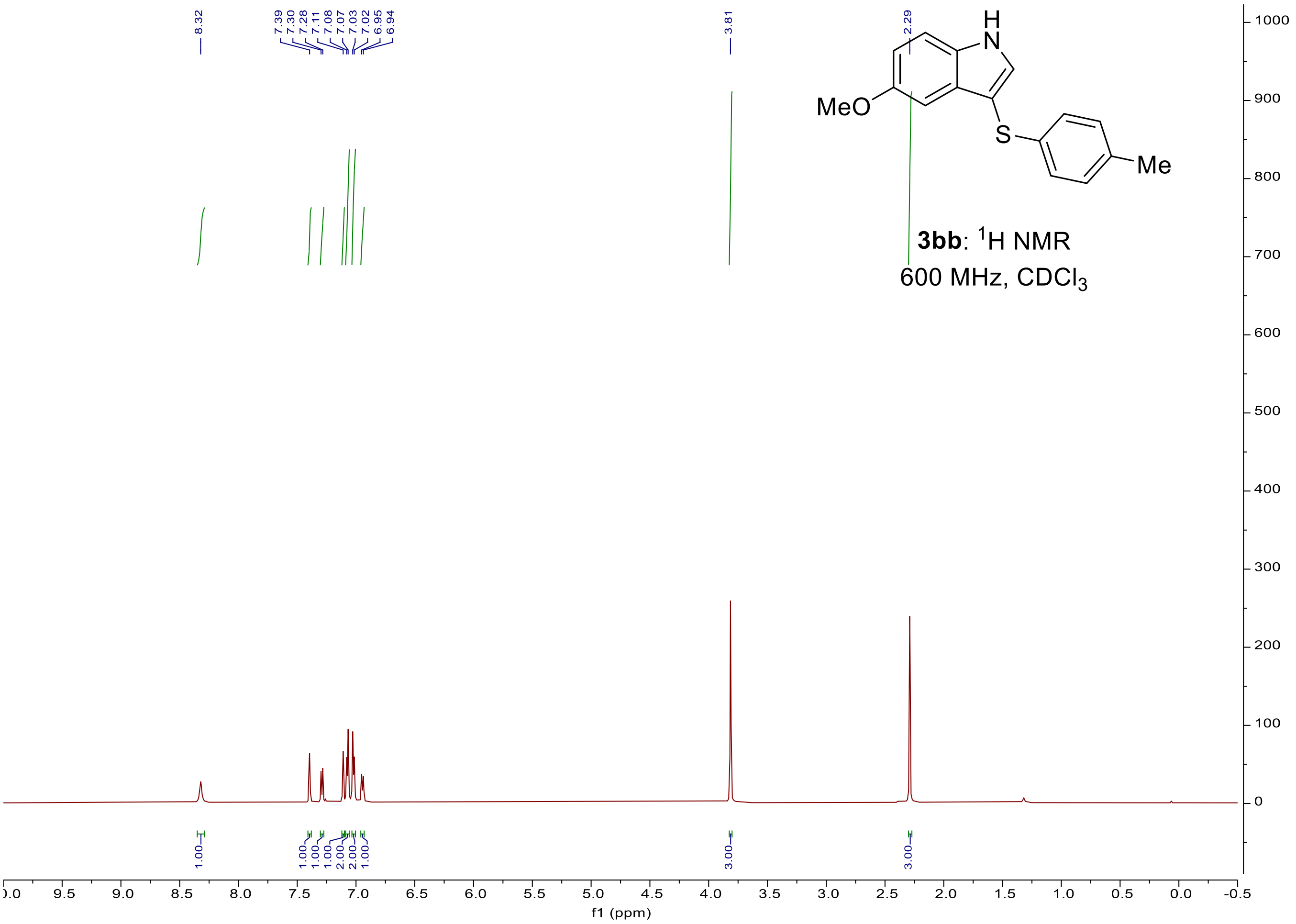
3bb: 1H NMR
600 MHz, CDCl3
MeO
S
Me
N
H
f1 (ppm)

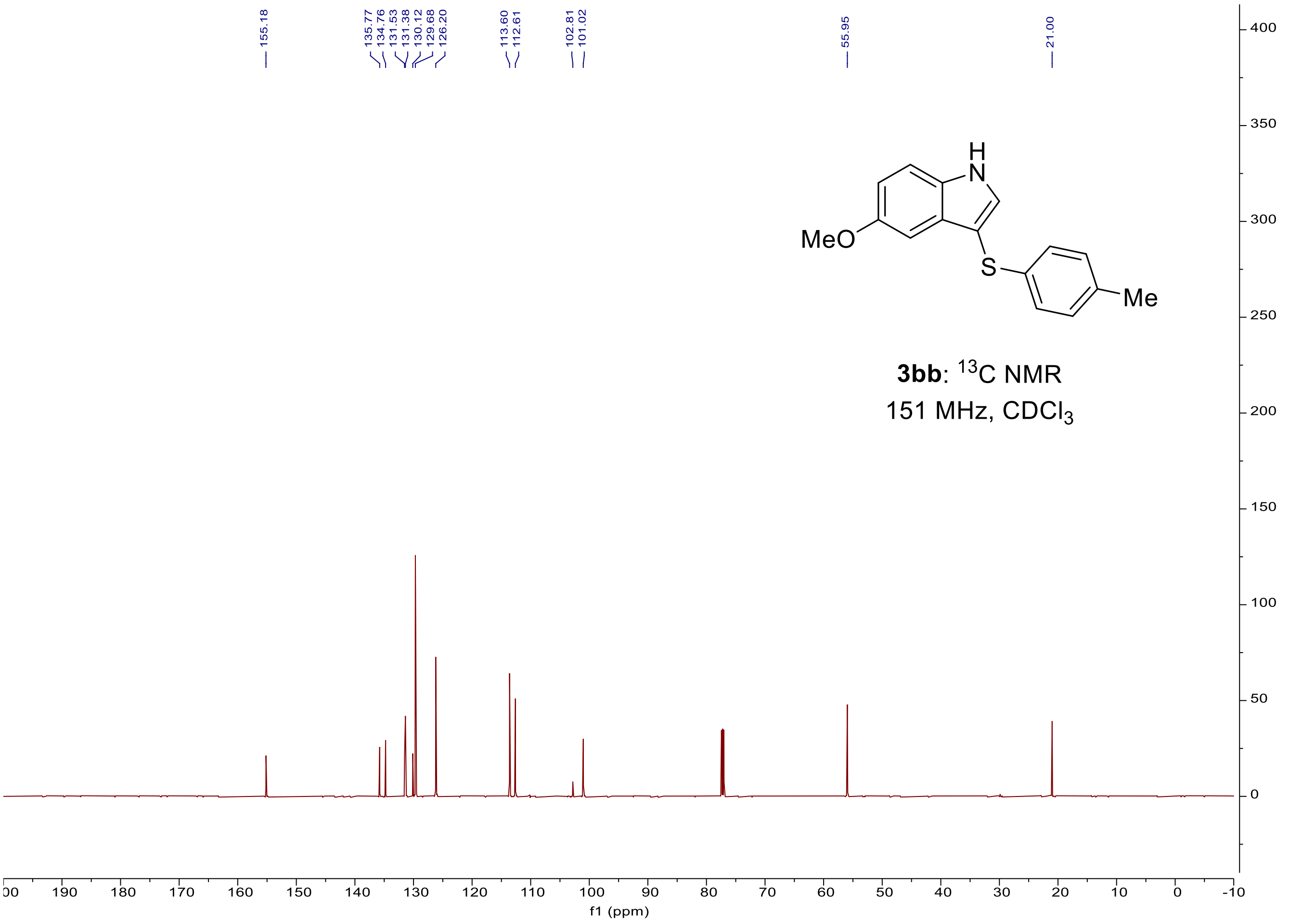
155.18
135.77
134.76
131.53
131.38
130.12
129.68
126.20
113.60
112.61
102.81
101.02
55.95
21.00
MeO
H
N
S
Me
3bb: ^{13}C NMR
151 MHz, $CDCl_3$
f1 (ppm)

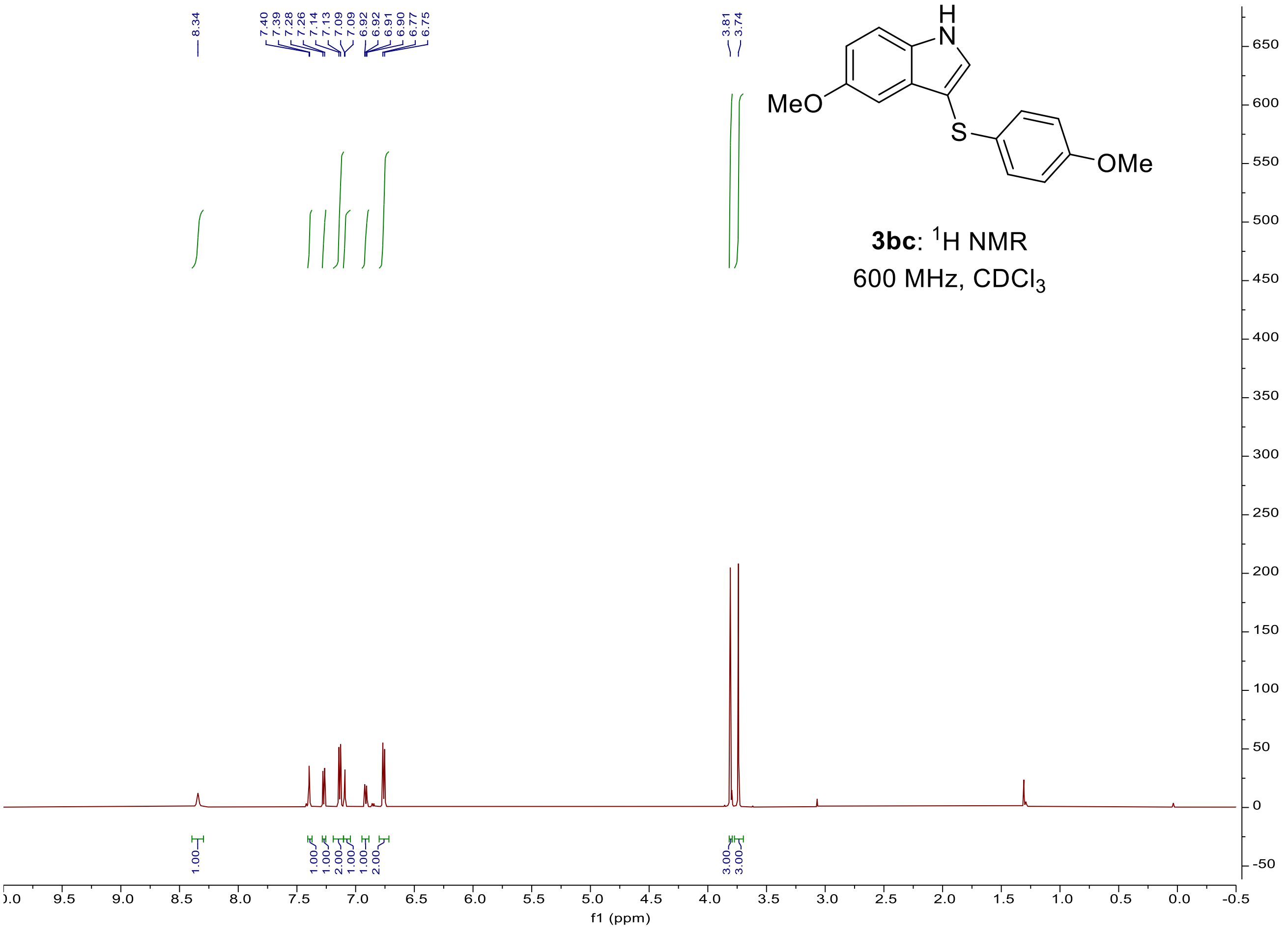
8.34
7.40
7.39
7.28
7.26
7.14
7.13
7.09
7.09
6.92
6.92
6.91
6.90
6.77
6.75
3.81
3.74
MeO
OMe
S
H
N
3bc: 1H NMR
600 MHz, CDCl3
1.00
1.00
1.00
2.00
1.00
1.00
2.00
3.00
3.00
f1 (ppm)

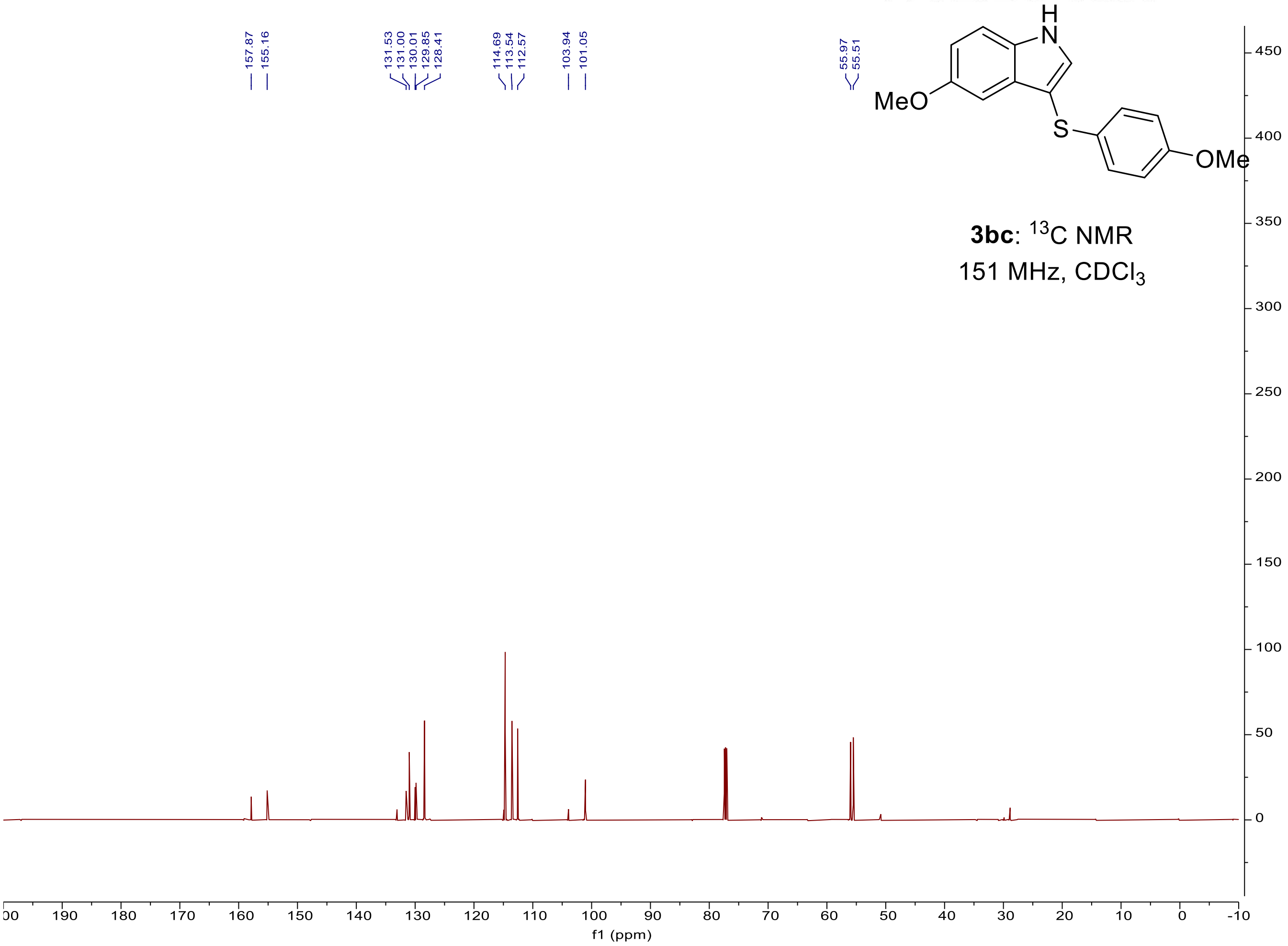
157.87
155.16
131.53
131.00
130.01
129.85
128.41
114.69
113.54
112.57
103.94
101.05
55.97
55.51
MeO
H
N
S
OMe
3bc: 13C NMR
151 MHz, CDCl3
f1 (ppm)

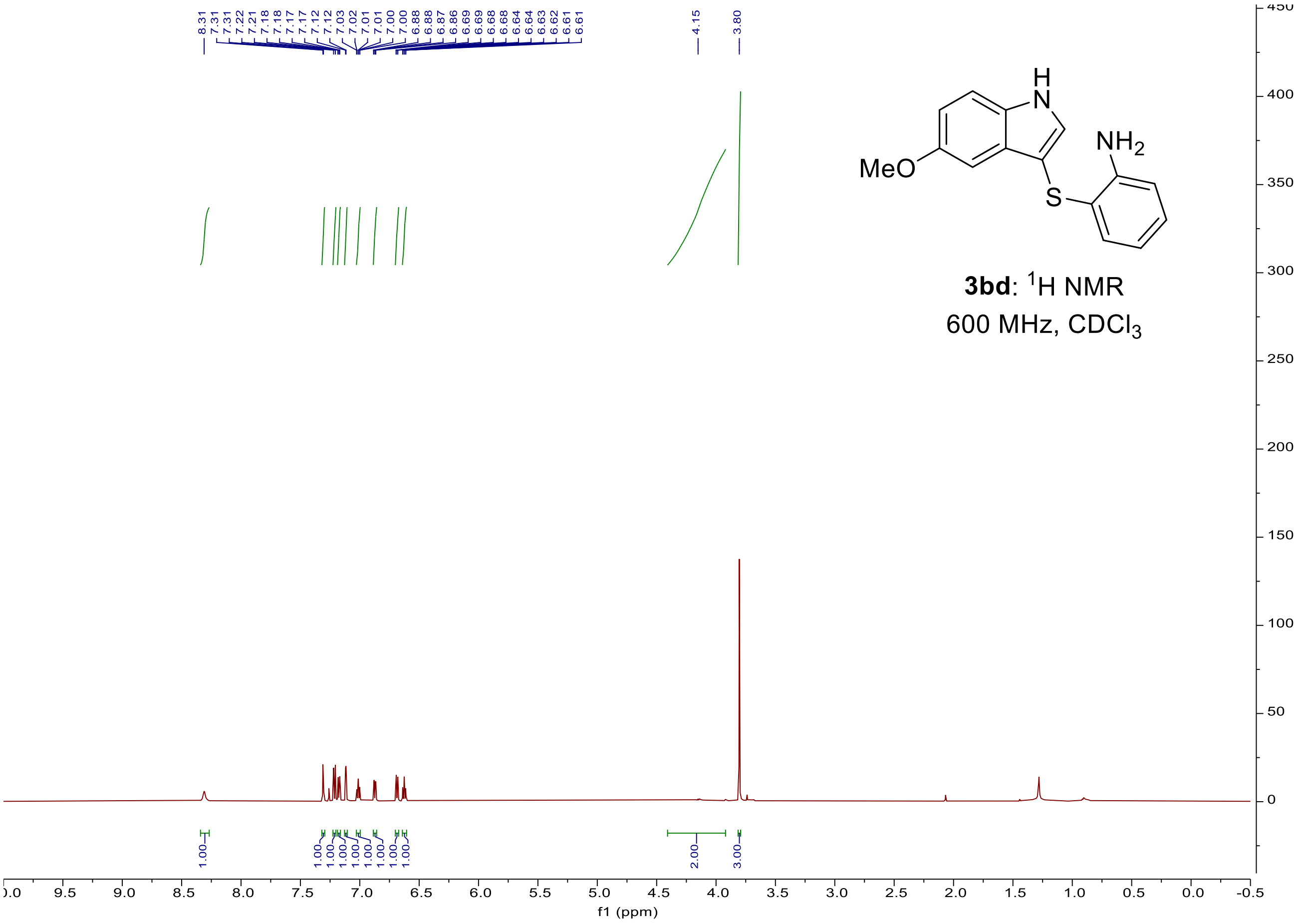

3bd: ¹H NMR
600 MHz, CDCl3
MeO
NH2
S
H
N
f1 (ppm)

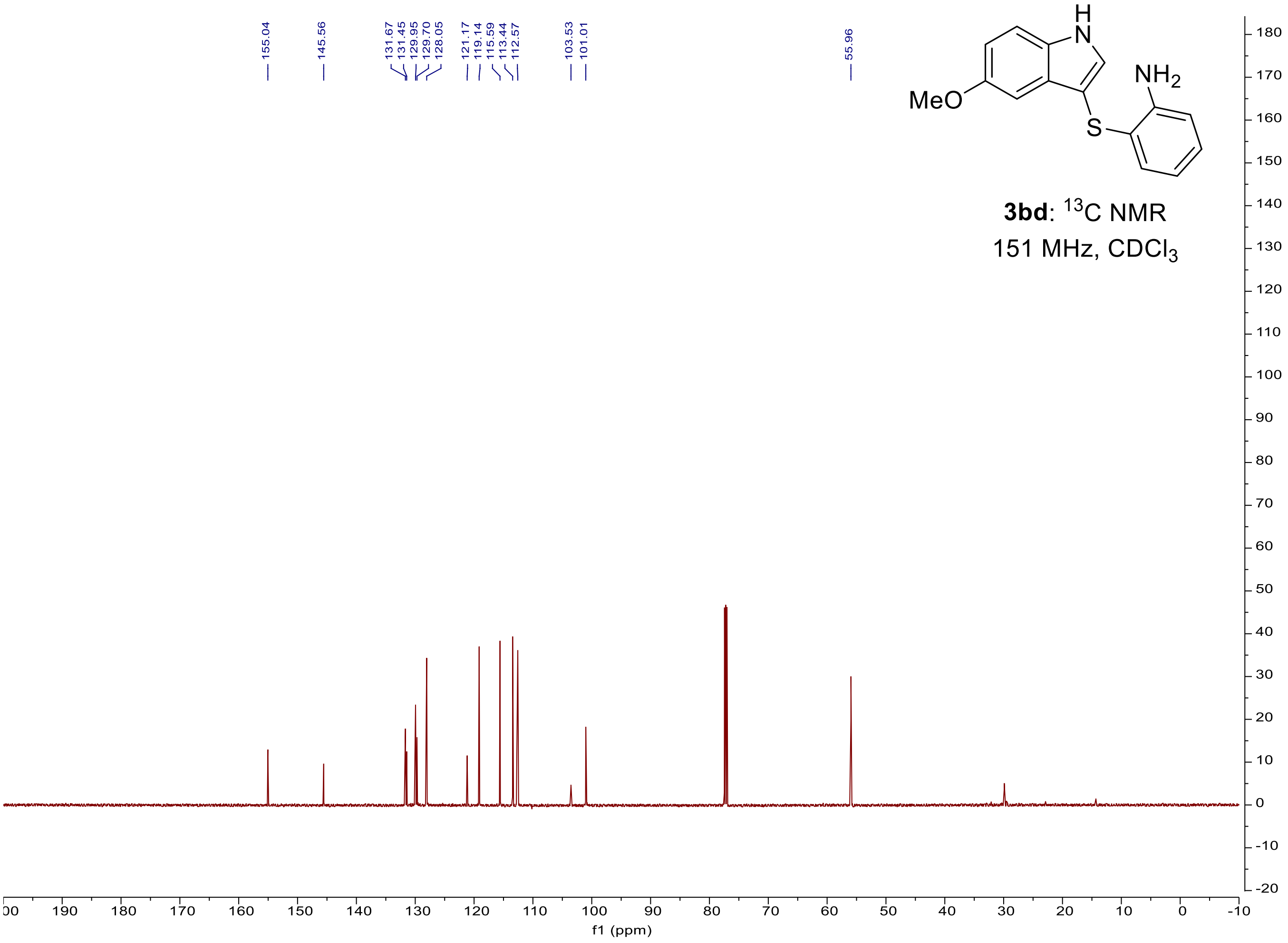

3bd: ^{13}C NMR
151 MHz, $CDCl_3$

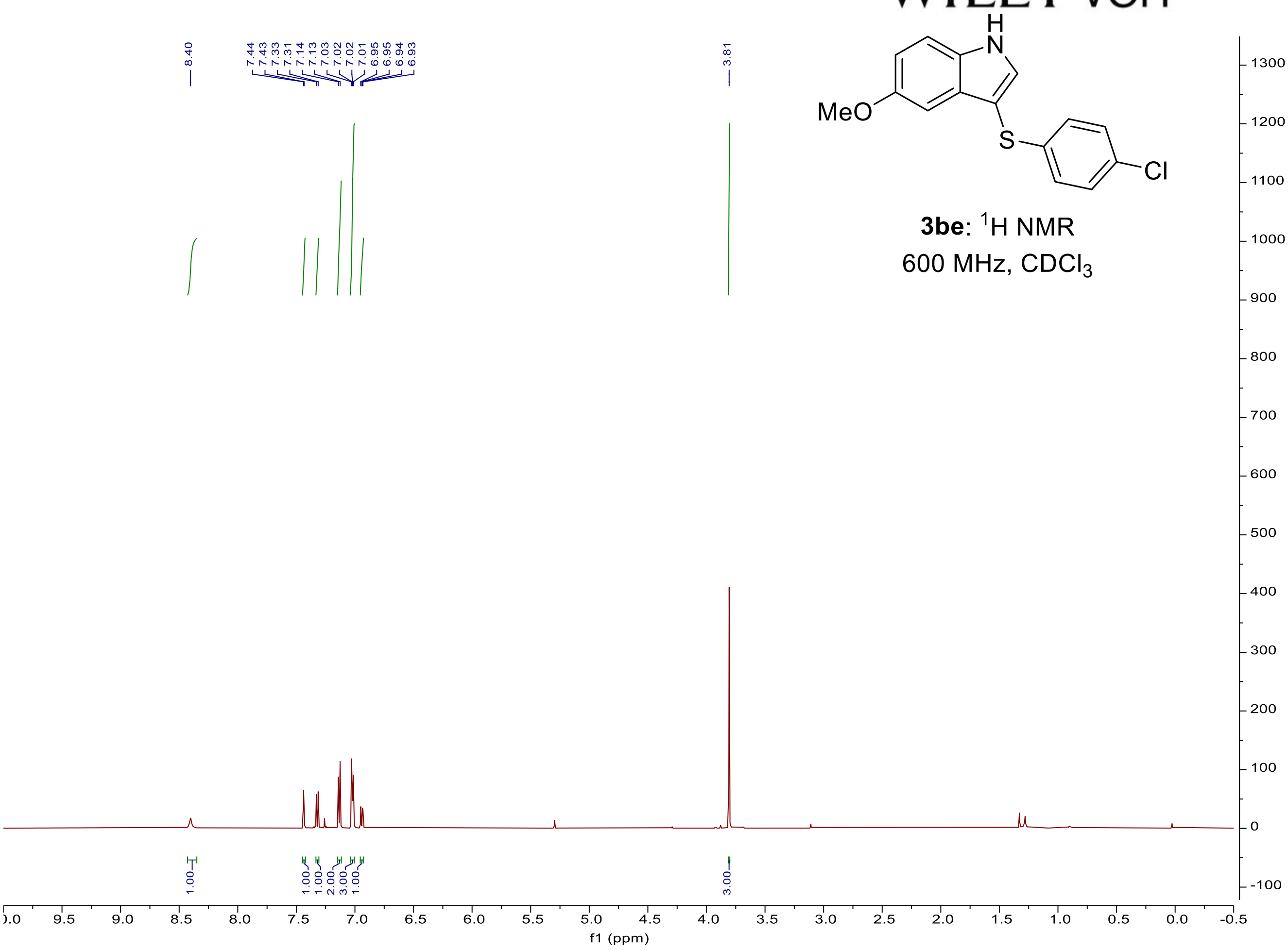

3be: ^1H NMR
600 MHz, $CDCl_3$

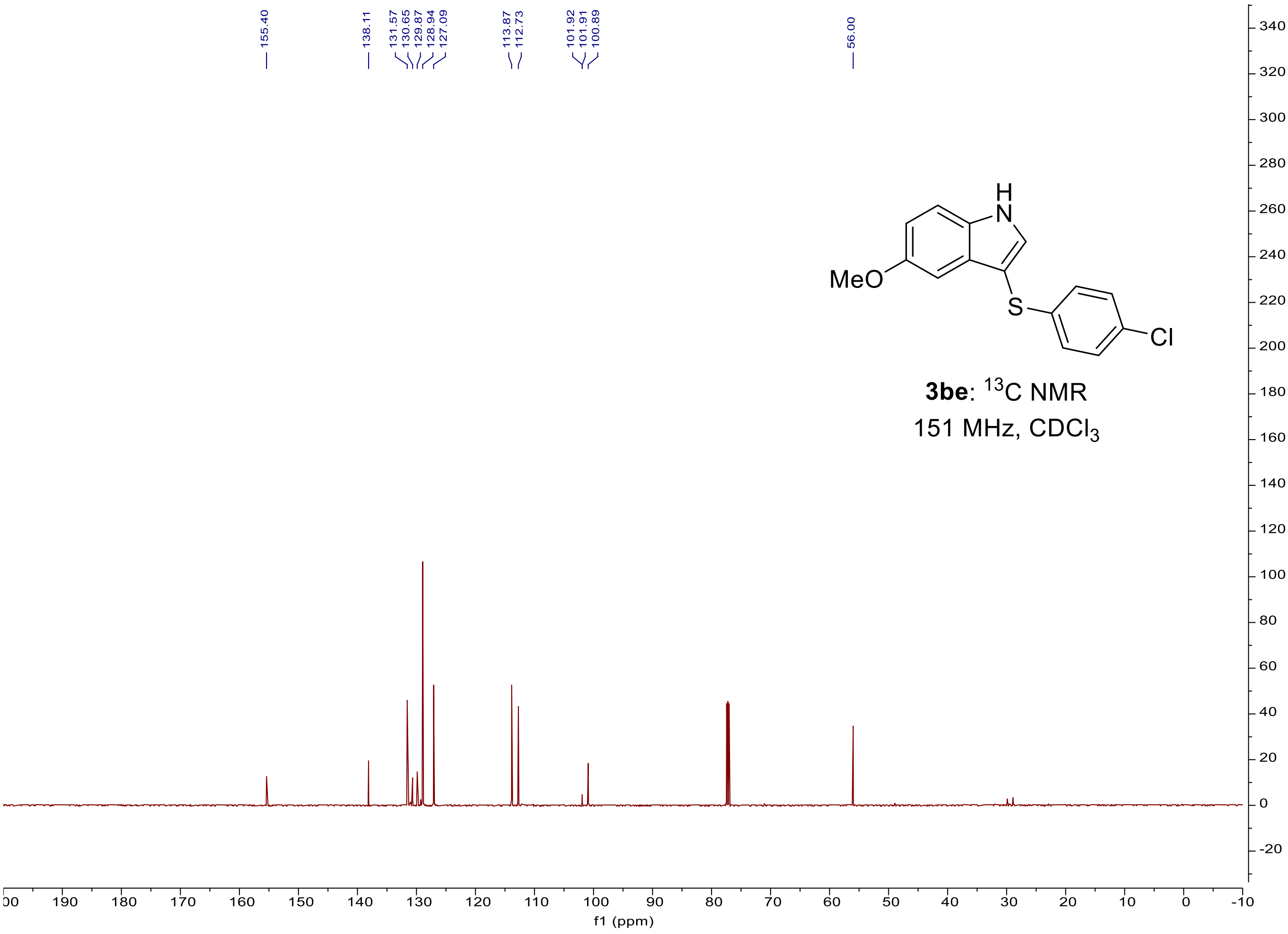
155.40
138.11
131.57
130.65
129.87
128.94
127.09
113.87
112.73
101.92
101.91
100.89
56.00
MeO
H
N
S
Cl
3be: 13C NMR
151 MHz, CDCl3
f1 (ppm)

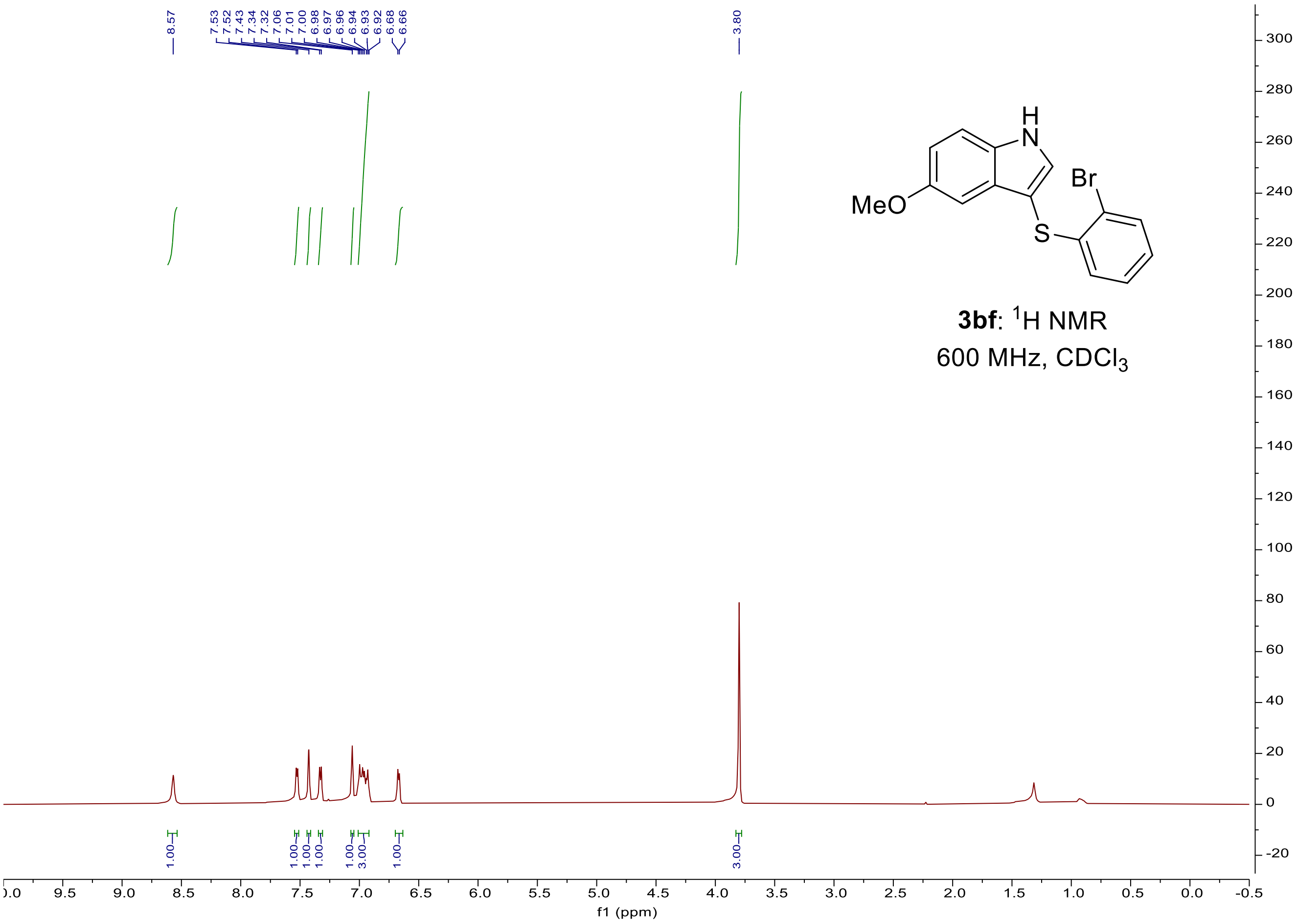
8.57
7.53
7.52
7.43
7.34
7.32
7.06
7.01
7.00
6.98
6.97
6.96
6.94
6.93
6.92
6.68
6.66
3.80
H
N
Br
MeO
S
3bf: 1H NMR
600 MHz, CDCl3
1.00
1.00
1.00
1.00
1.00
3.00
1.00
3.00
f1 (ppm)

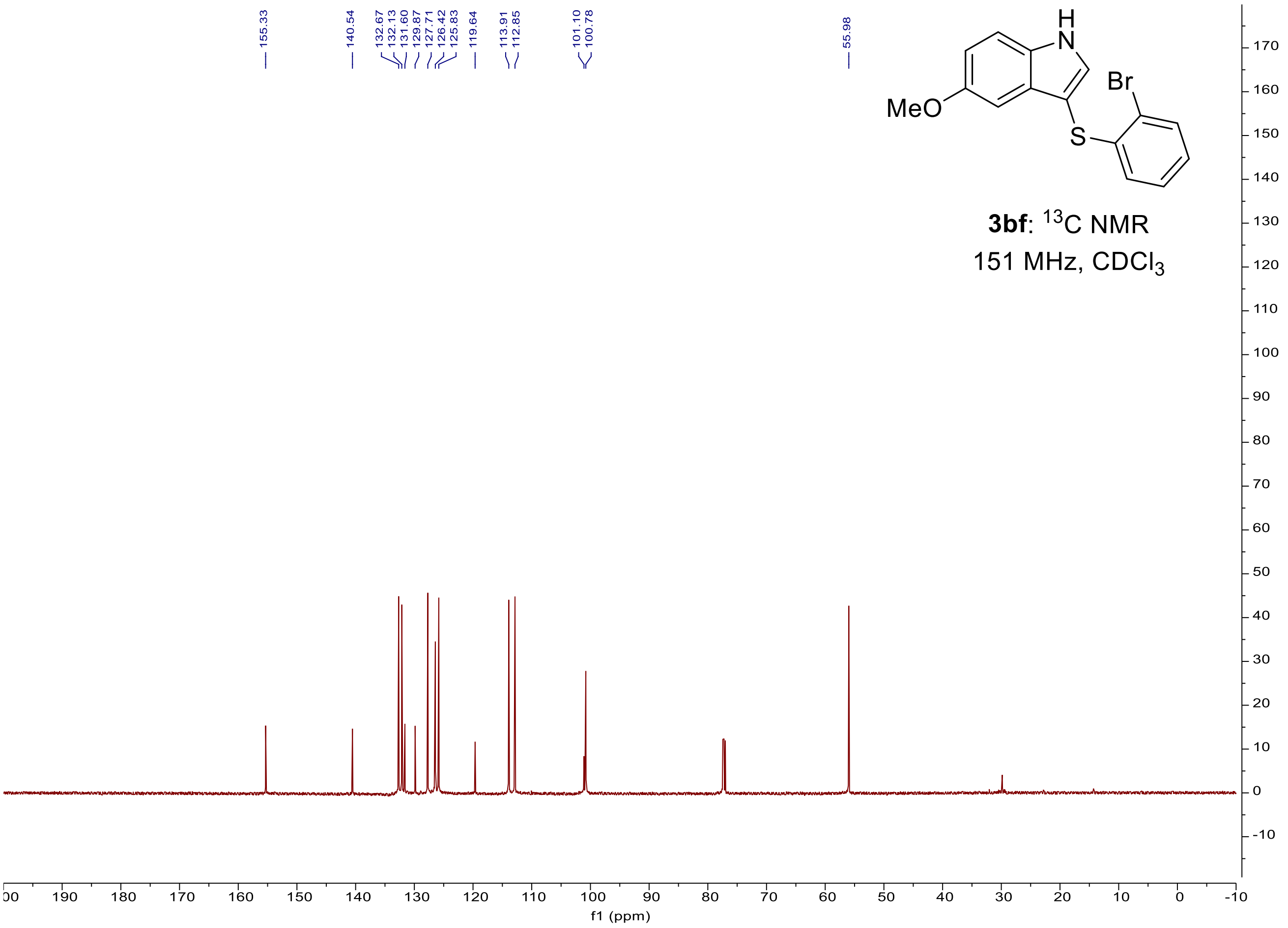
155.33
140.54
132.67
132.13
131.60
129.87
127.71
126.42
125.83
119.64
113.91
112.85
101.10
100.78
55.98
MeO
H
N
Br
S
3bf: ^{13}C NMR
151 MHz, $CDCl_3$
f1 (ppm)

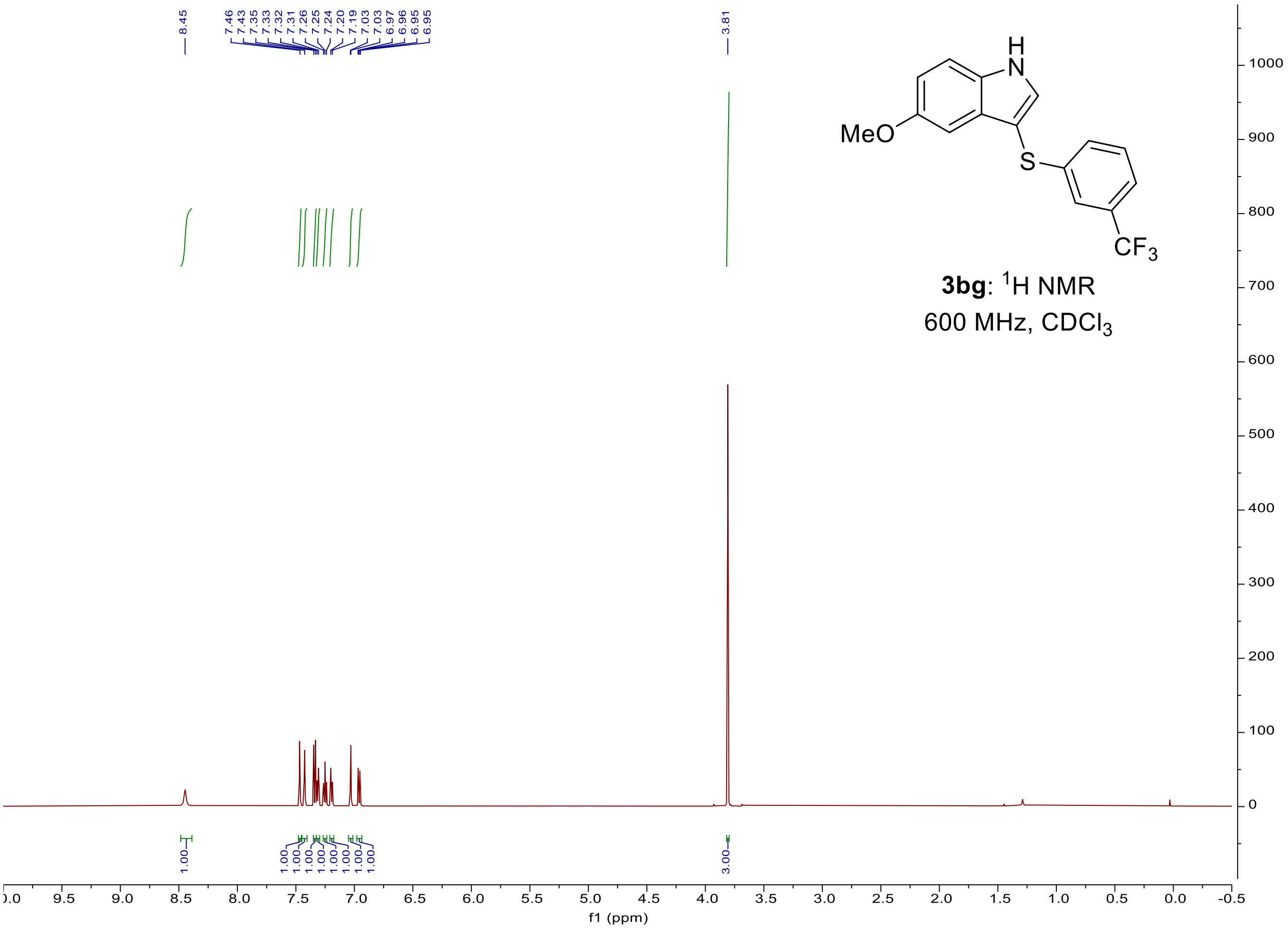
3bg: ^{1}H NMR
600 MHz, $CDCl_3$
MeO
H
N
S
CF_3
f1 (ppm)

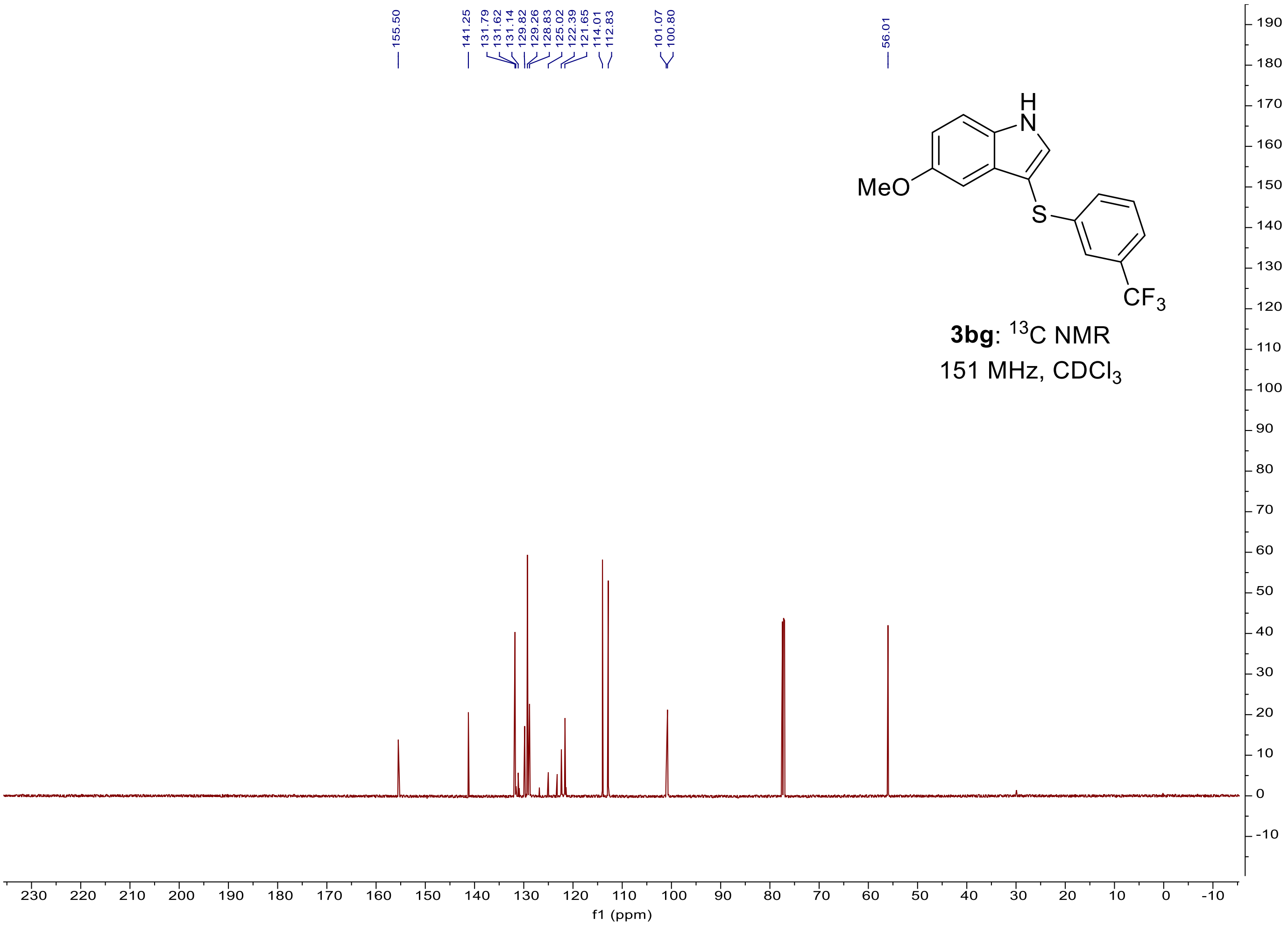
155.50
141.25
131.79
131.62
131.14
129.82
129.26
128.83
125.02
122.39
121.65
114.01
112.83
101.07
100.80
56.01
MeO
H
N
S
CF3
3bg: 13C NMR
151 MHz, CDCl3
f1 (ppm)

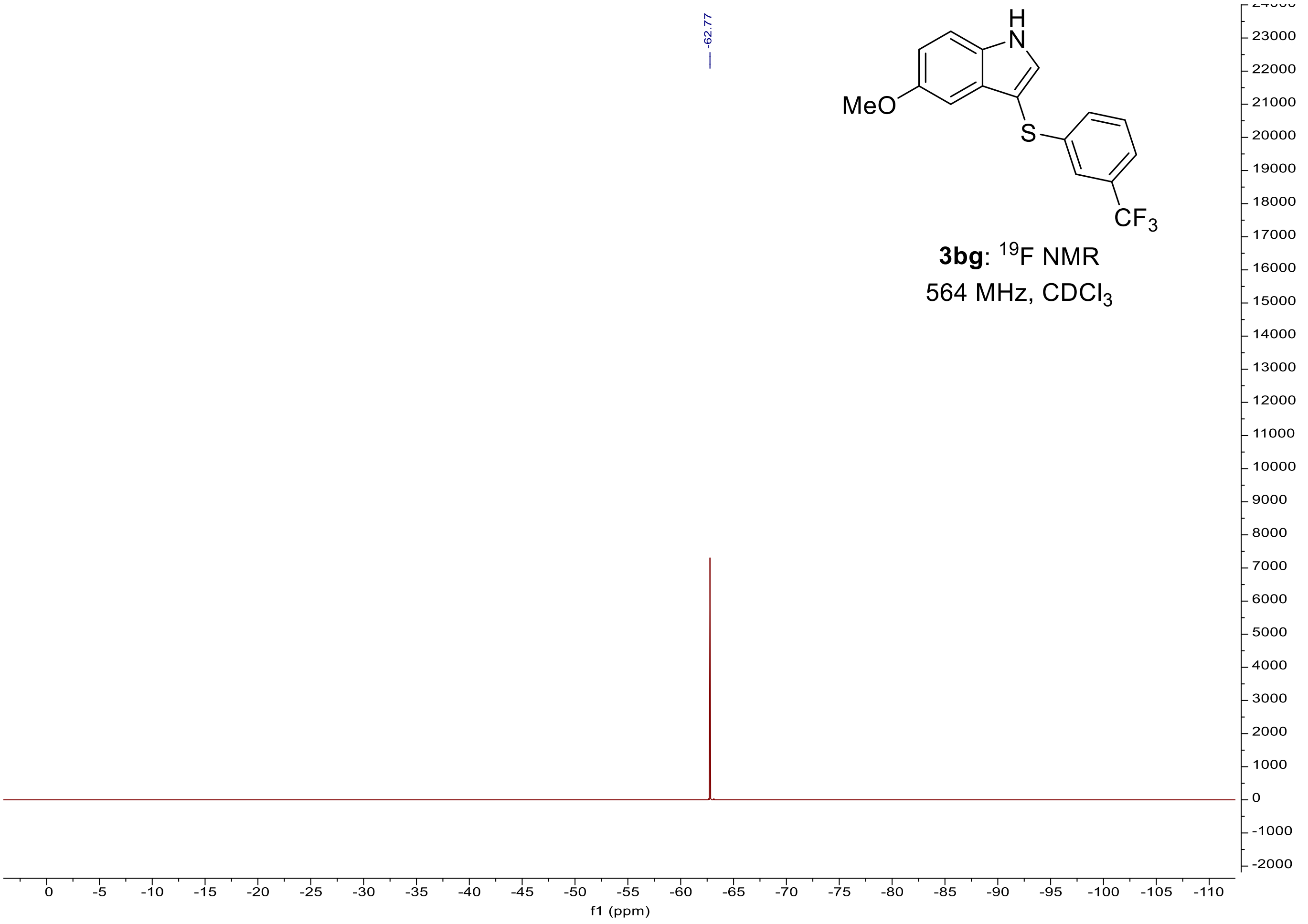

-62.77
H
N
MeO
S
CF3
3bg: 19F NMR
564 MHz, CDCl3
23000
22000
21000
20000
19000
18000
17000
16000
15000
14000
13000
12000
11000
10000
9000
8000
7000
6000
5000
4000
3000
2000
1000
0
-1000
-2000
0 -5 -10 -15 -20 -25 -30 -35 -40 -45 -50 -55 -60 -65 -70 -75 -80 -85 -90 -95 -100 -105 -110
f1 (ppm)

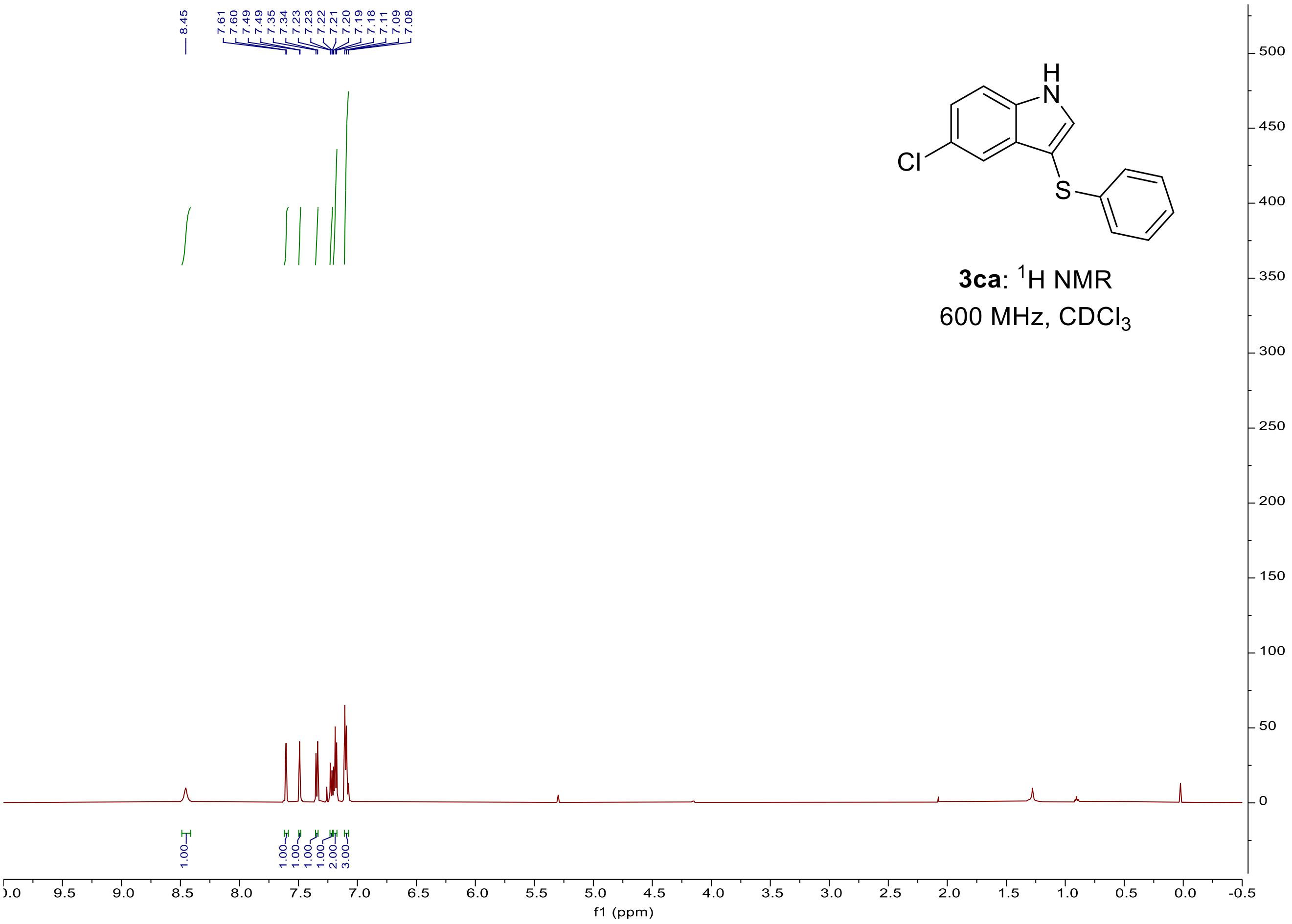

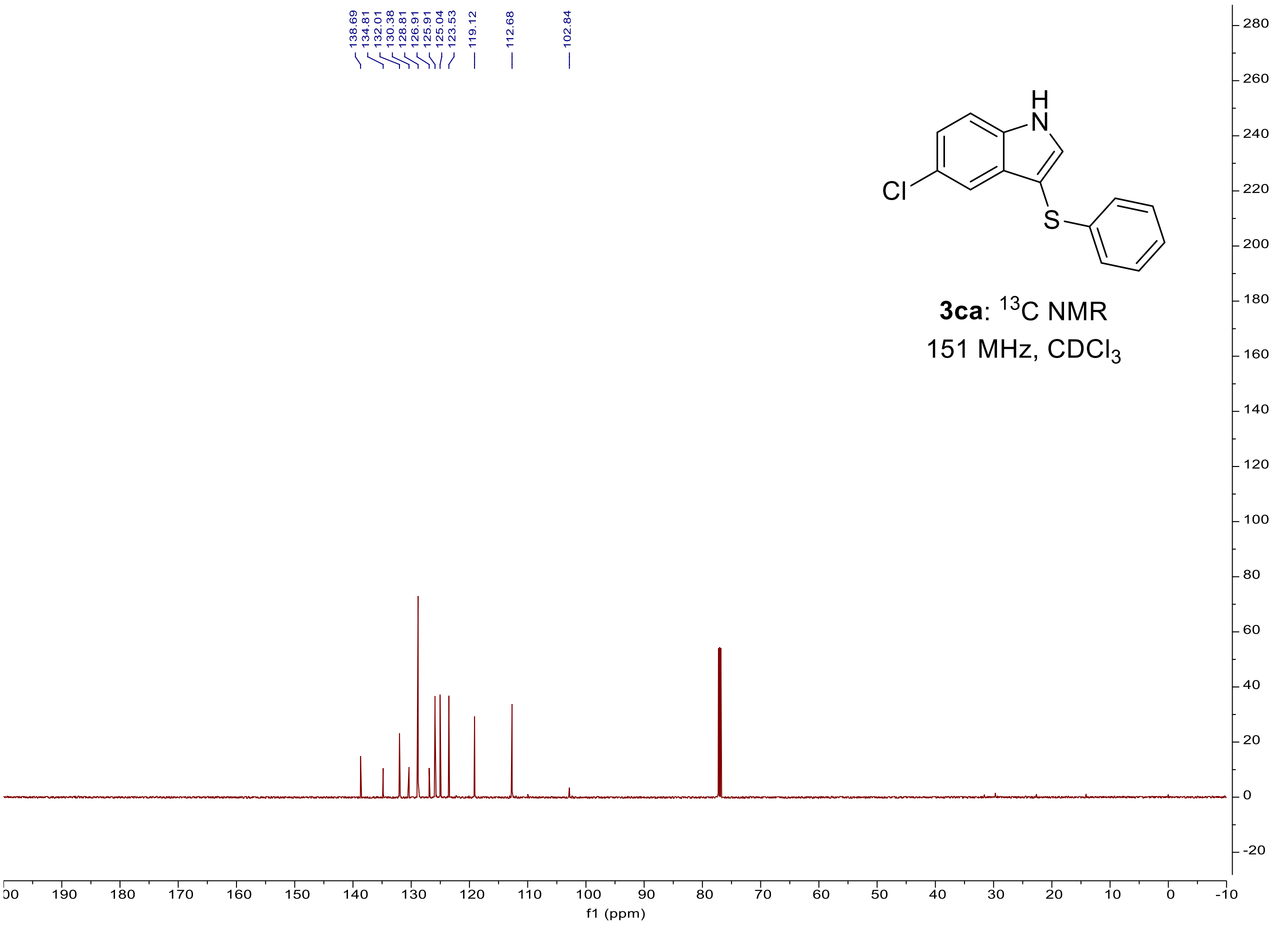

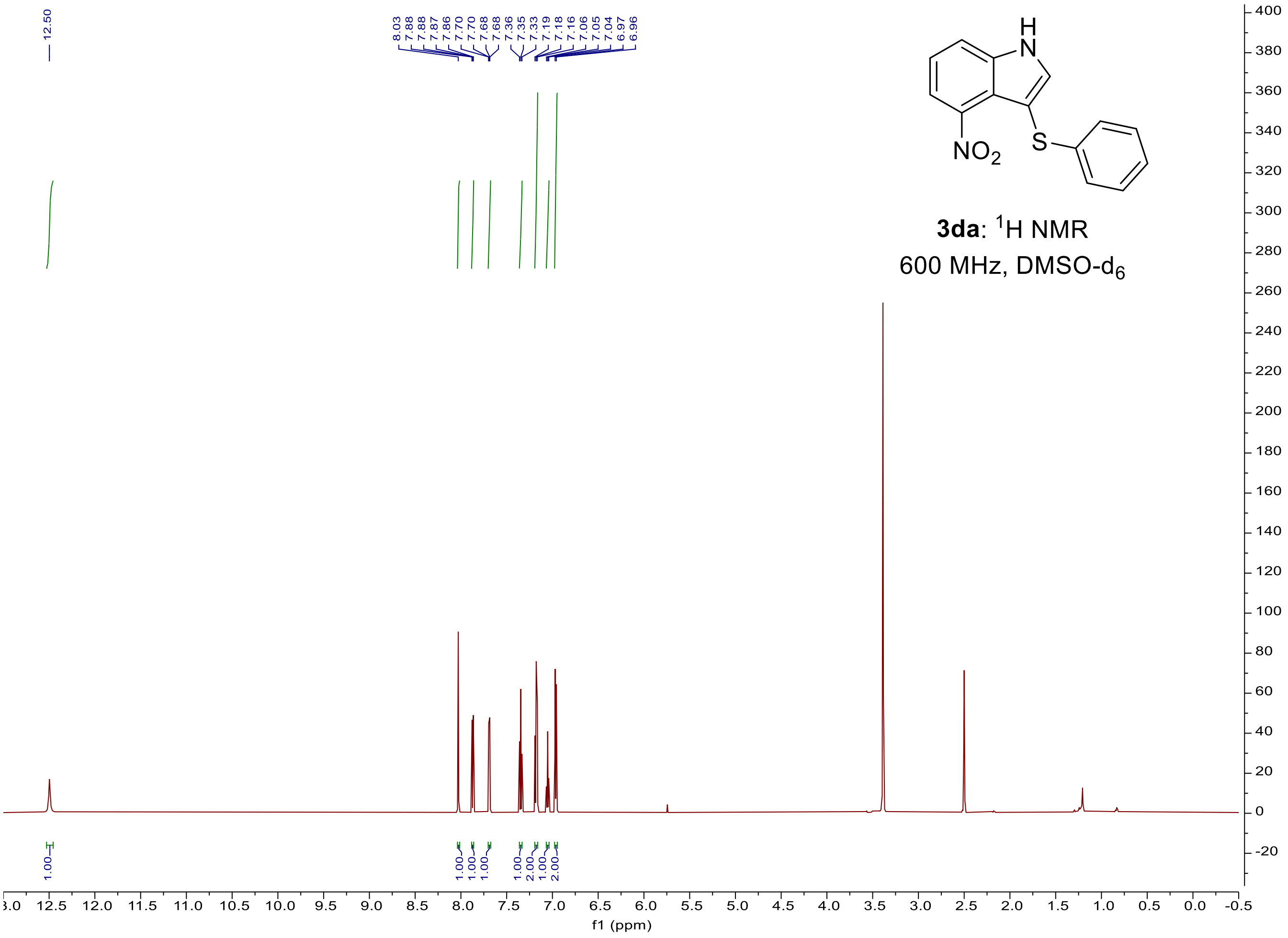
3da: 1H NMR
600 MHz, DMSO-d6
NO2
S
H
N
f1 (ppm)

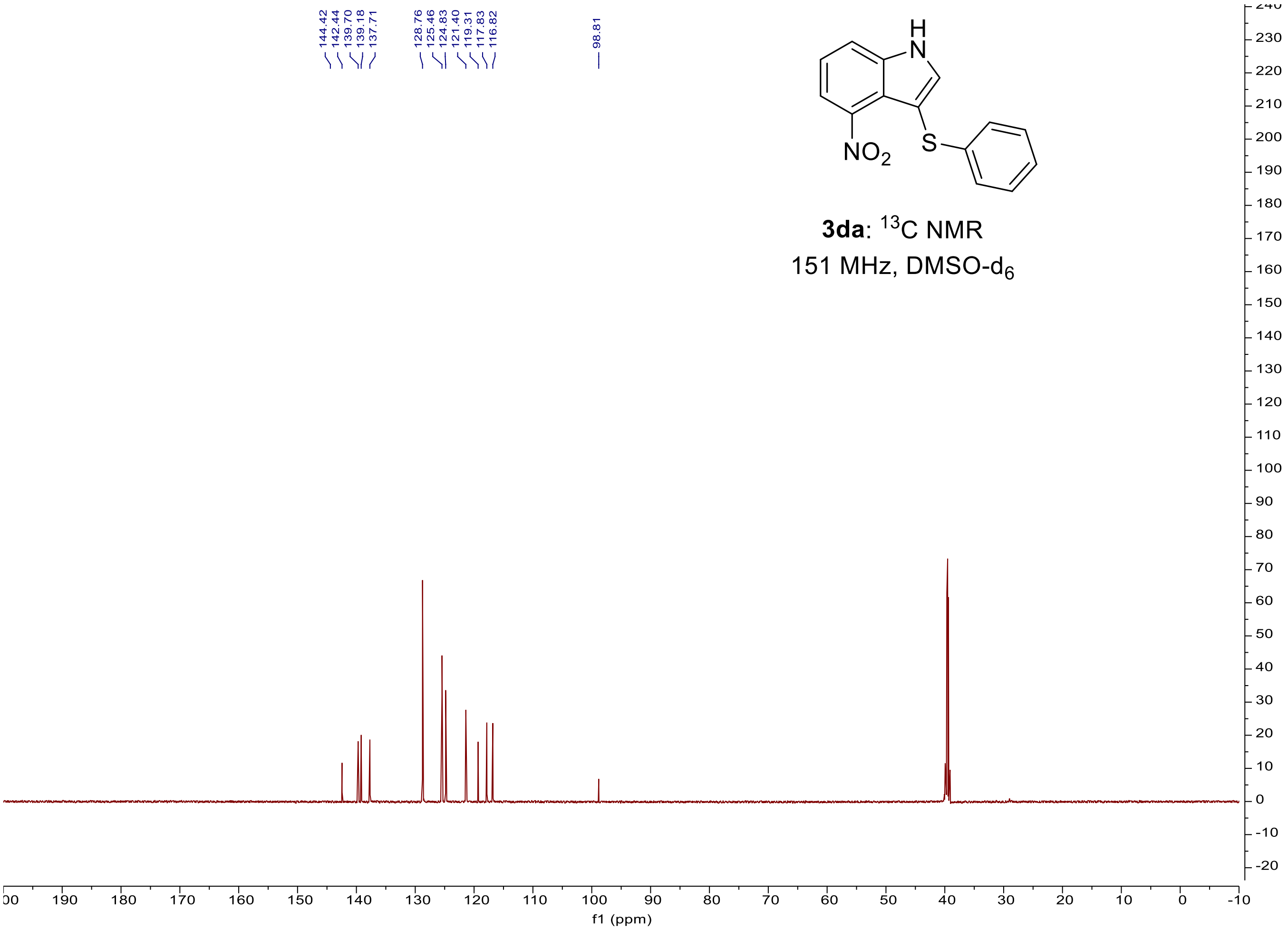
144.42
142.44
139.70
139.18
137.71
128.76
125.46
124.83
121.40
119.31
117.83
116.82
98.81
H
N
NO2
S
3da: 13C NMR
151 MHz, DMSO-d6
f1 (ppm)

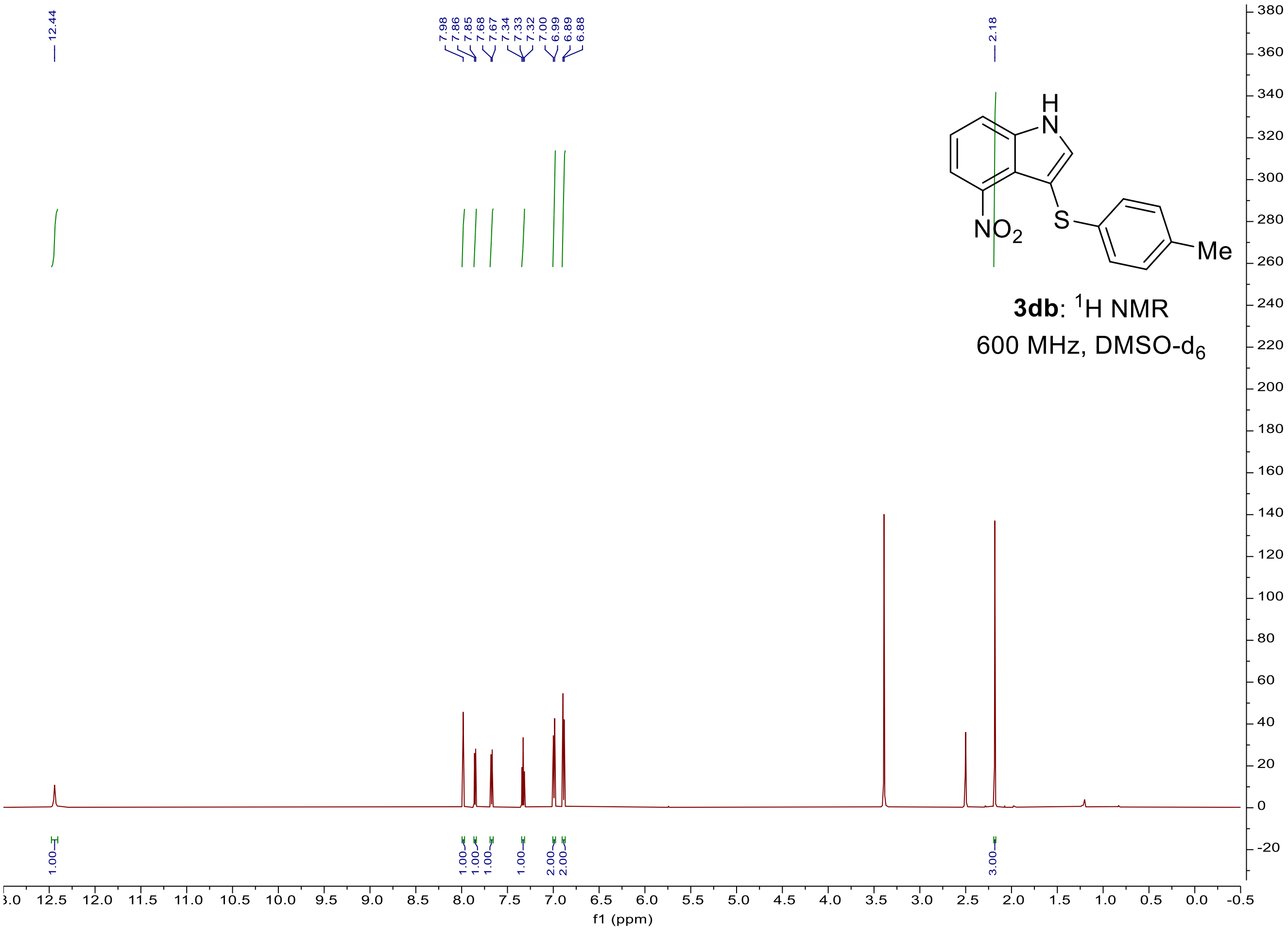
12.44
7.98
7.86
7.85
7.68
7.67
7.34
7.33
7.32
7.00
6.99
6.89
6.88
2.18
H
N
NO2
S
Me
3db: 1H NMR
600 MHz, DMSO-d6
1.00
1.00
1.00
1.00
1.00
2.00
2.00
3.00
f1 (ppm)

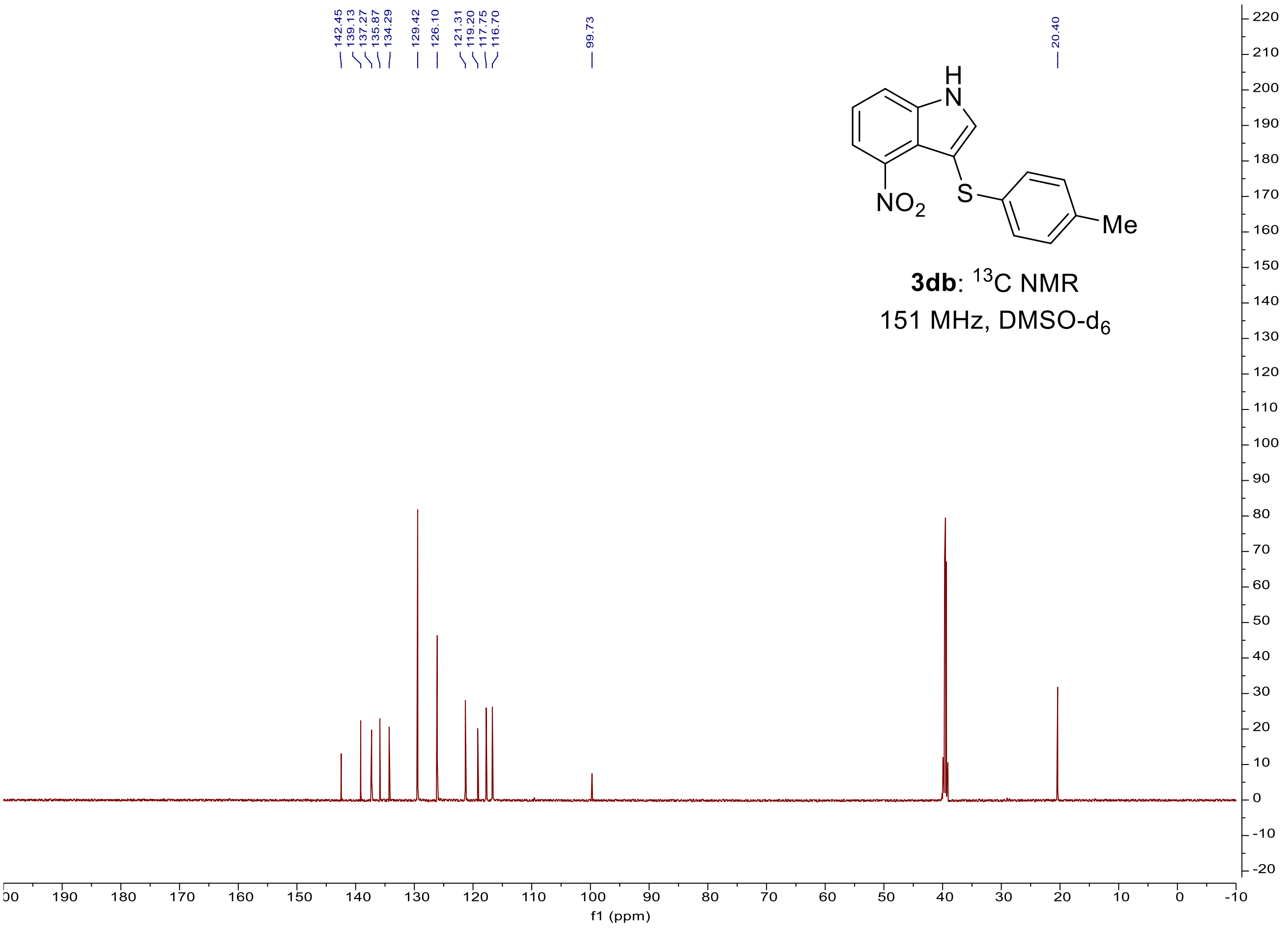
142.45
139.13
137.27
135.87
134.29
129.42
126.10
121.31
119.20
117.75
116.70
99.73
20.40
H
N
NO2
S
Me
3db: 13C NMR
151 MHz, DMSO-d6
f1 (ppm)

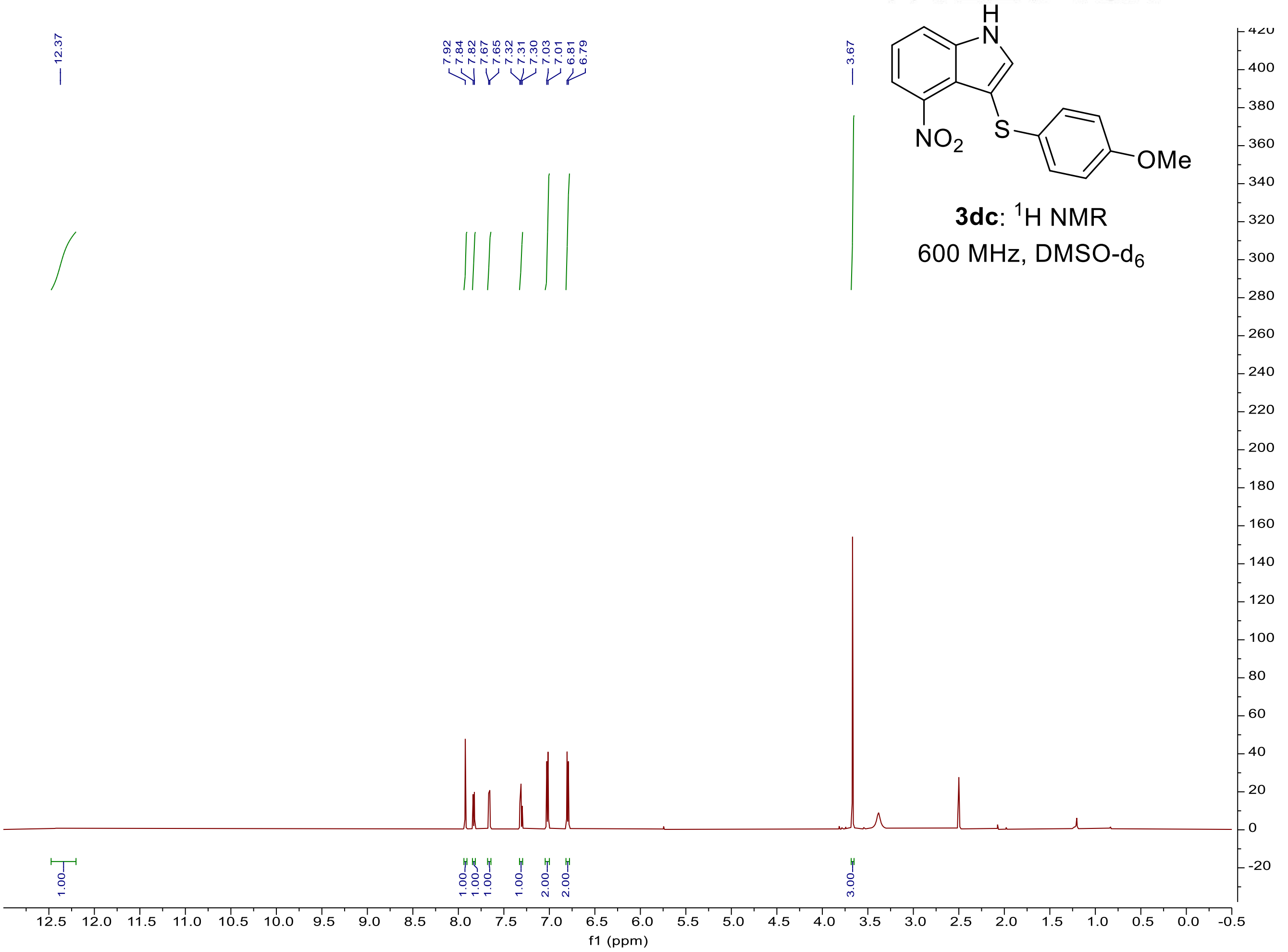
12.37
7.92
7.84
7.82
7.67
7.65
7.32
7.31
7.30
7.03
7.01
6.81
6.79
3.67
H
N
NO2
S
OMe
3dc: 1H NMR
600 MHz, DMSO-d6
1.00
1.00
1.00
1.00
1.00
2.00
2.00
3.00
f1 (ppm)

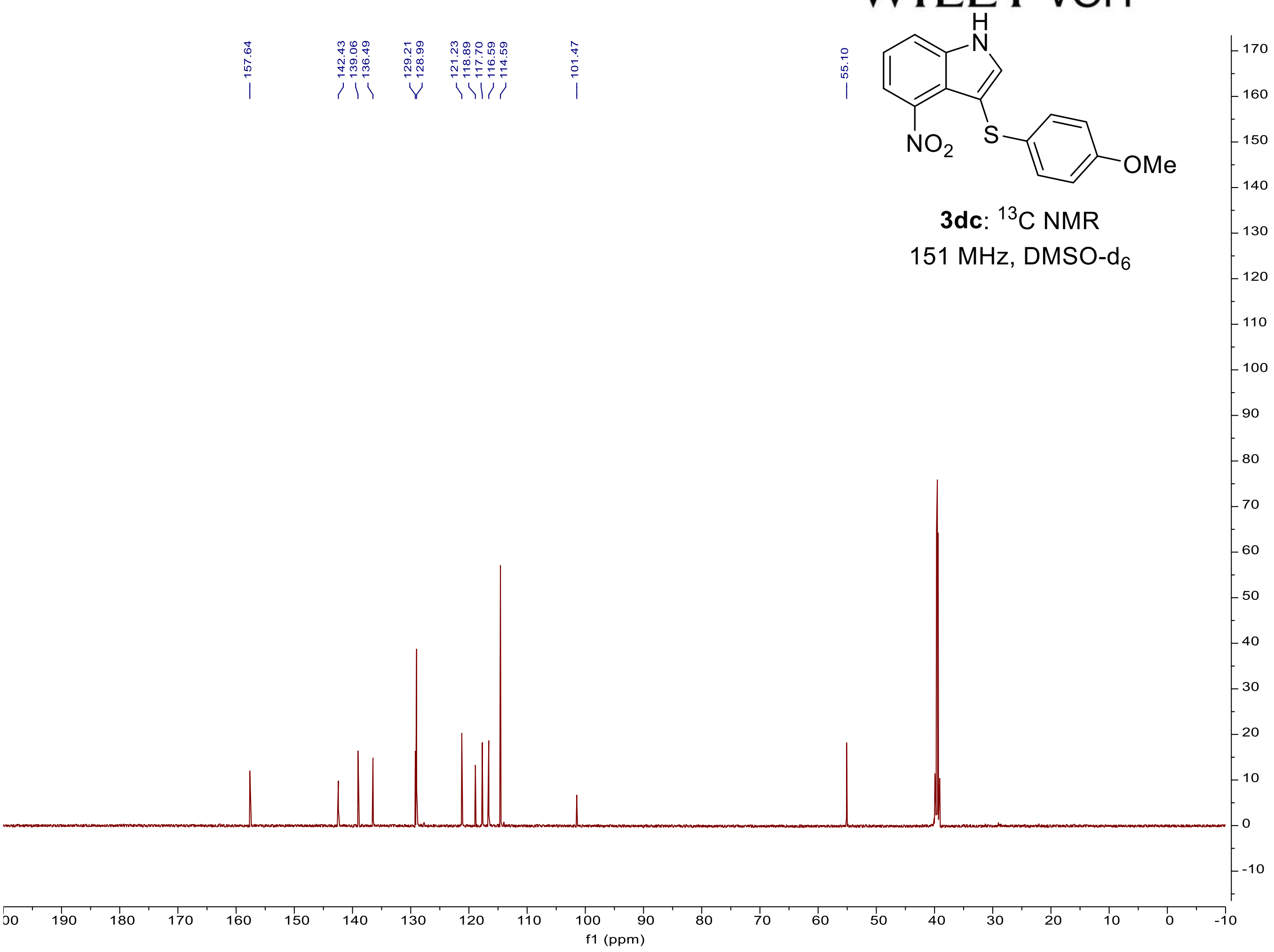
157.64
142.43
139.06
136.49
129.21
128.99
121.23
118.89
117.70
116.59
114.59
101.47
55.10
NO2
S
OMe
H
N
3dc: 13C NMR
151 MHz, DMSO-d6
f1 (ppm)

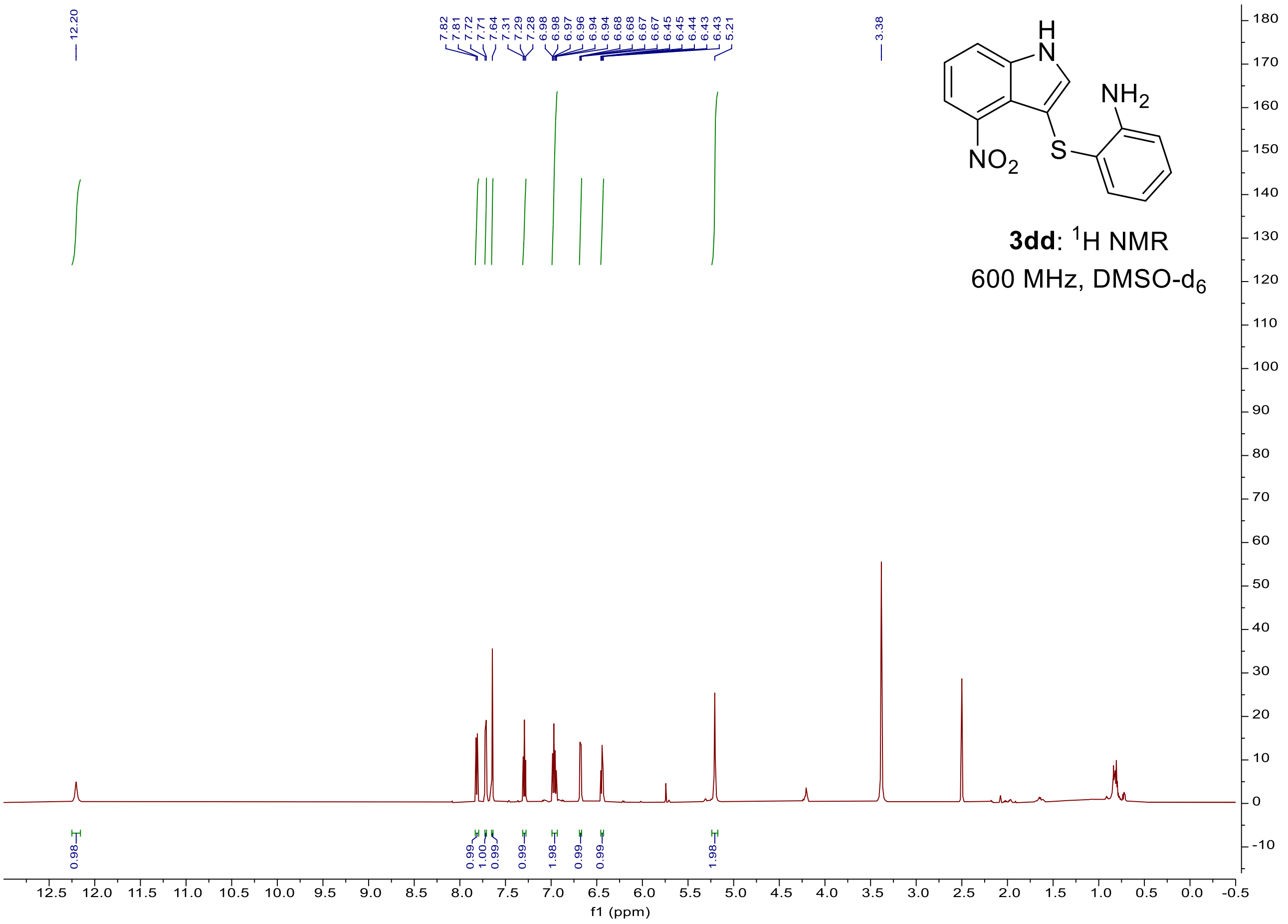
3dd: ¹H NMR
600 MHz, DMSO-d6
NH2
NO2
S
H
N
f1 (ppm)

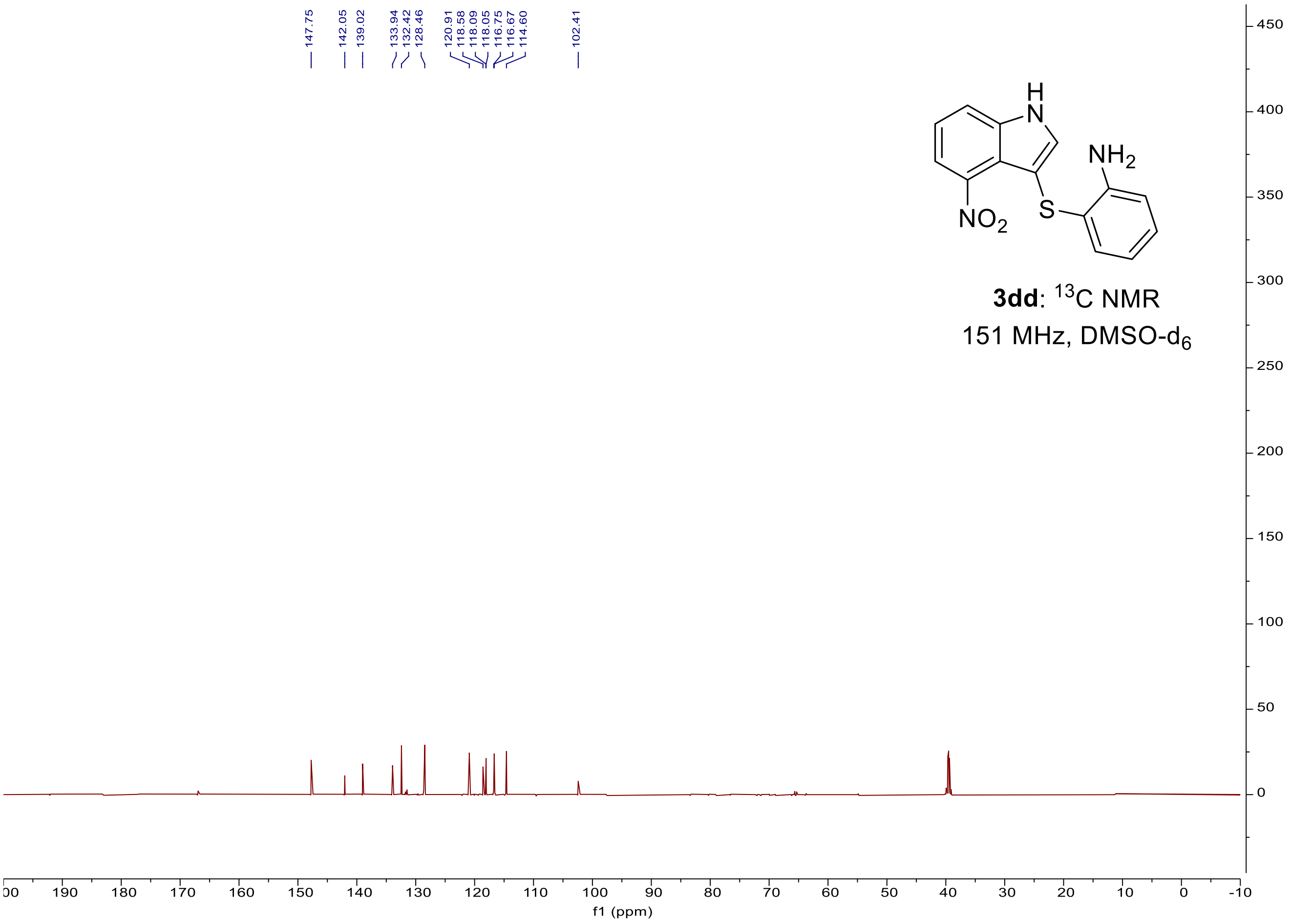
3dd: ¹³C NMR
151 MHz, DMSO-d6
NH2
NO2
S
H
N
f1 (ppm)

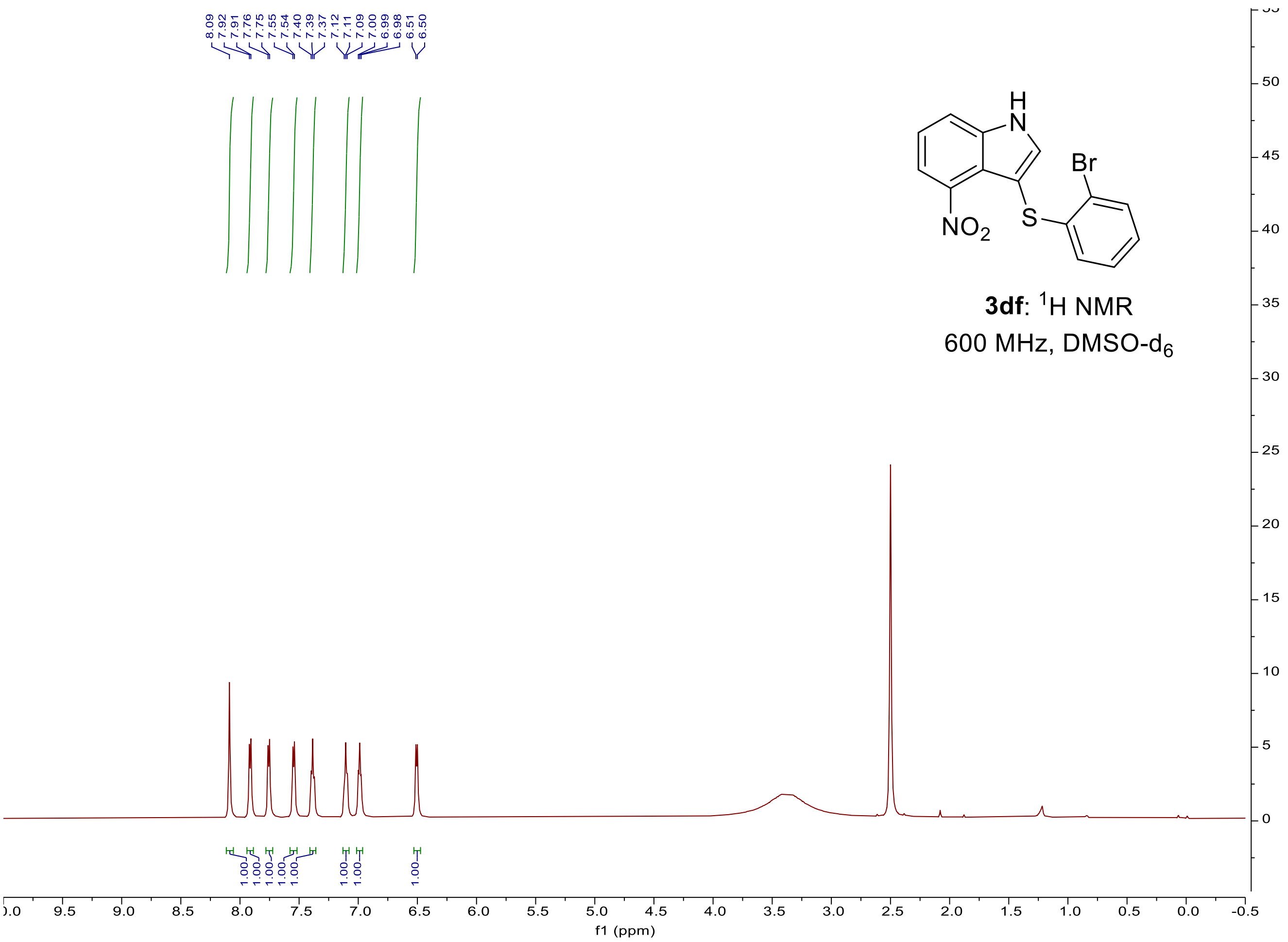

8.09 7.92 7.91 7.76 7.75 7.55 7.54 7.40 7.39 7.37 7.12 7.11 7.09 7.00 6.99 6.98 6.51 6.50
NO2
S
Br
H
N
3df: 1H NMR
600 MHz, DMSO-d6
1.00 1.00 1.00 1.00 1.00 1.00 1.00 1.00
f1 (ppm)

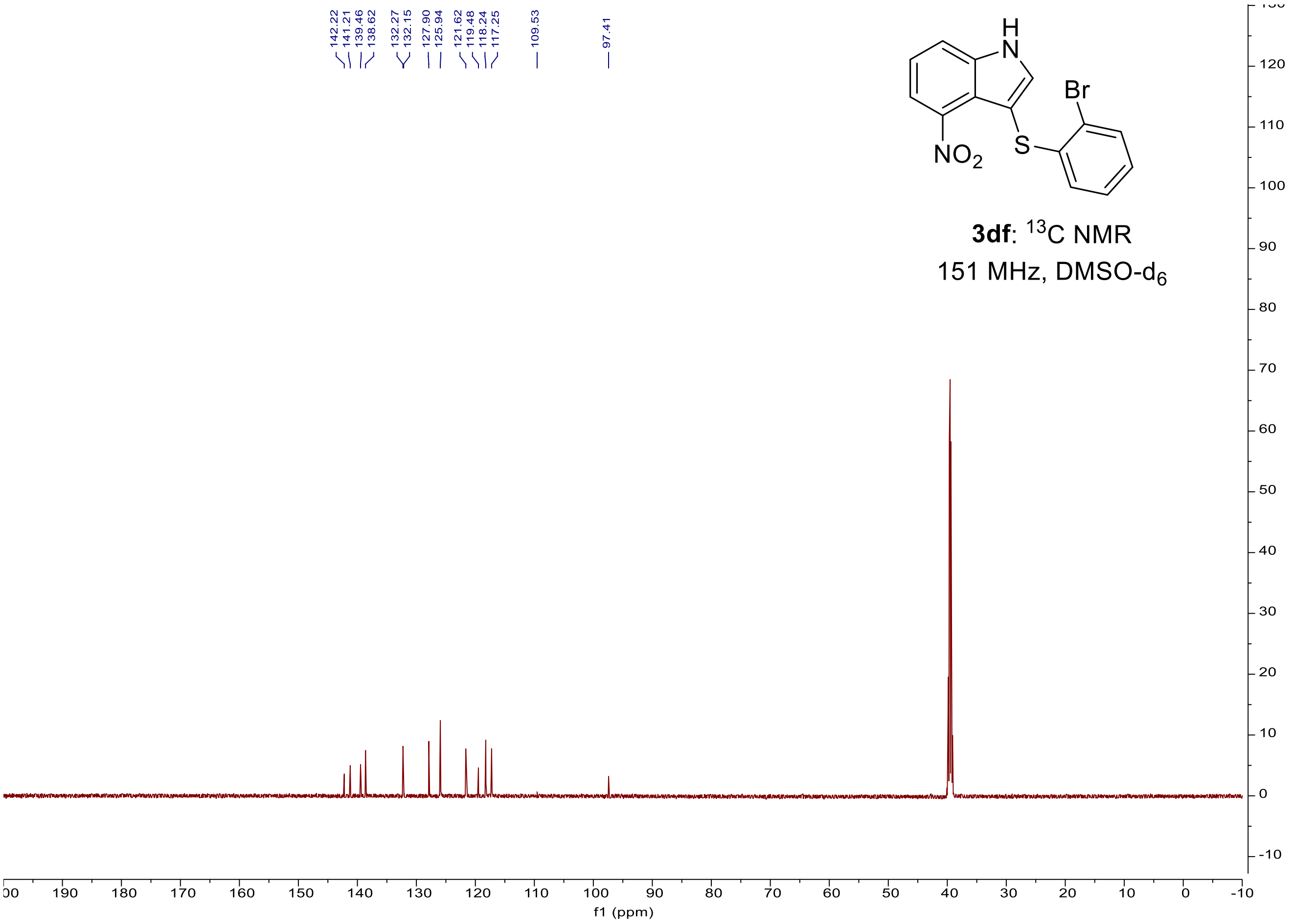

3df: ^{13}C NMR
151 MHz, DMSO-d_6

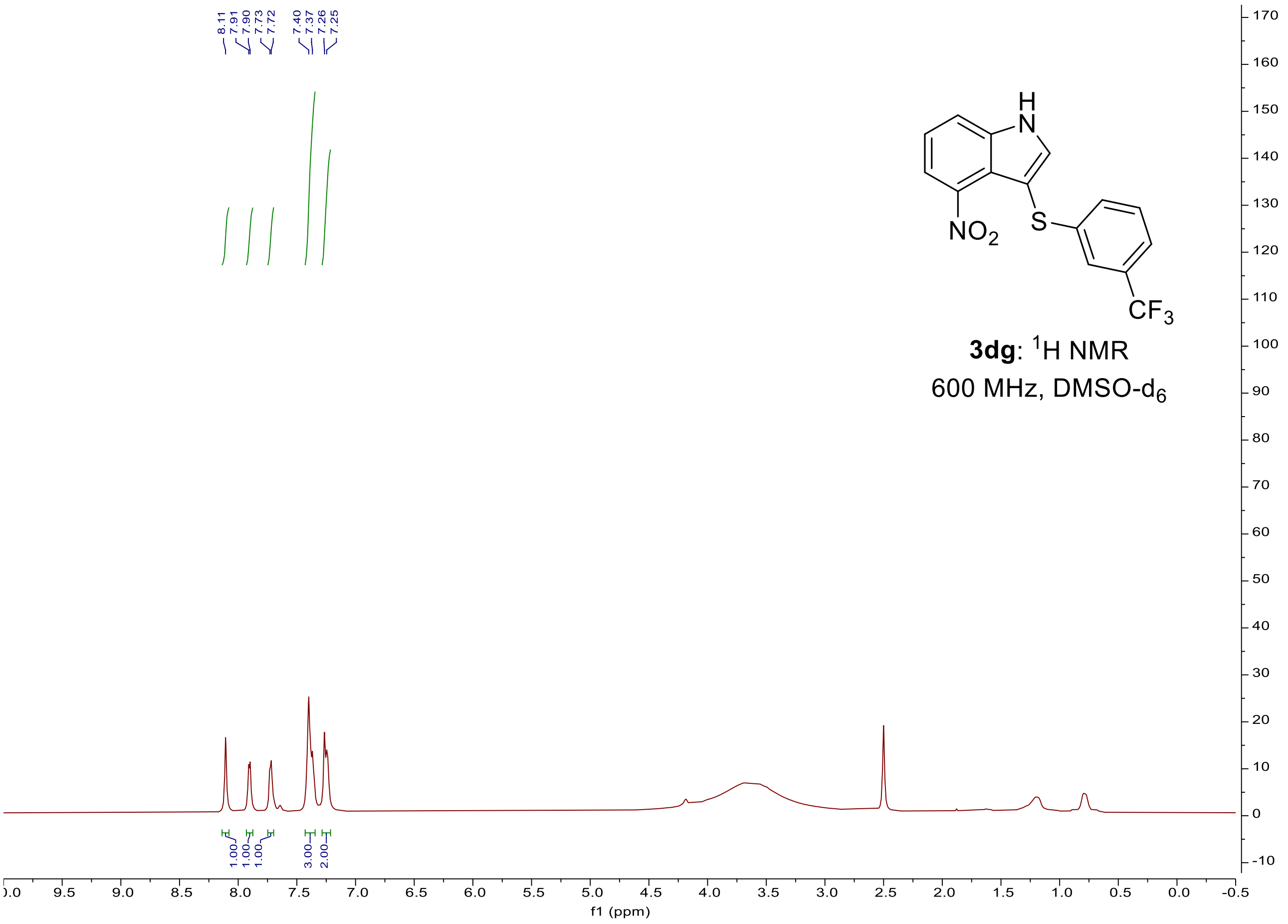

3dg: ^{1}H NMR
600 MHz, DMSO-d_6

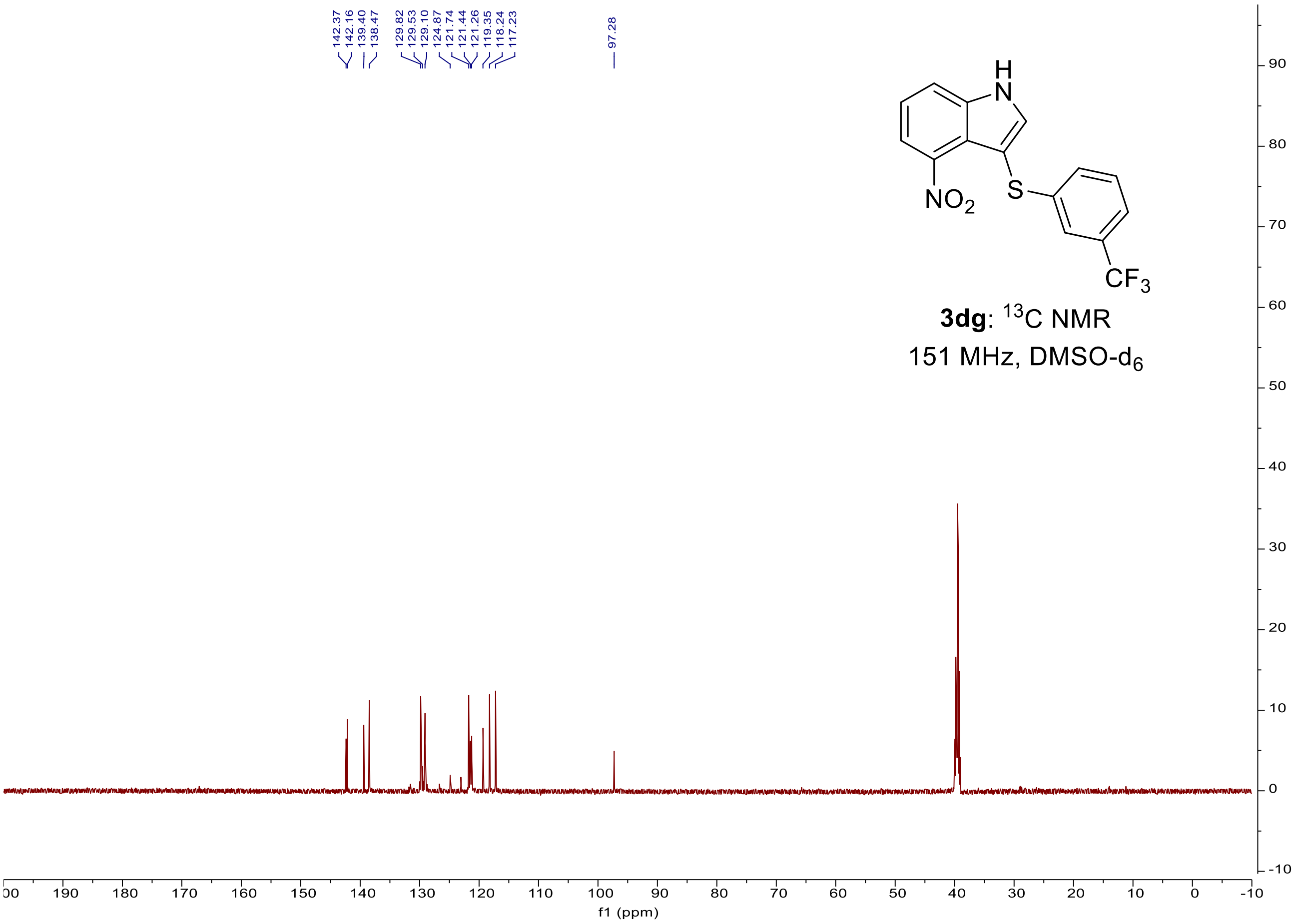
142.37
142.16
139.40
138.47
129.82
129.53
129.10
124.87
121.74
121.44
121.26
119.35
118.24
117.23
97.28
H
N
NO_2
S
CF_3
3dg: ^{13}C NMR
151 MHz, DMSO-d_6
f1 (ppm)

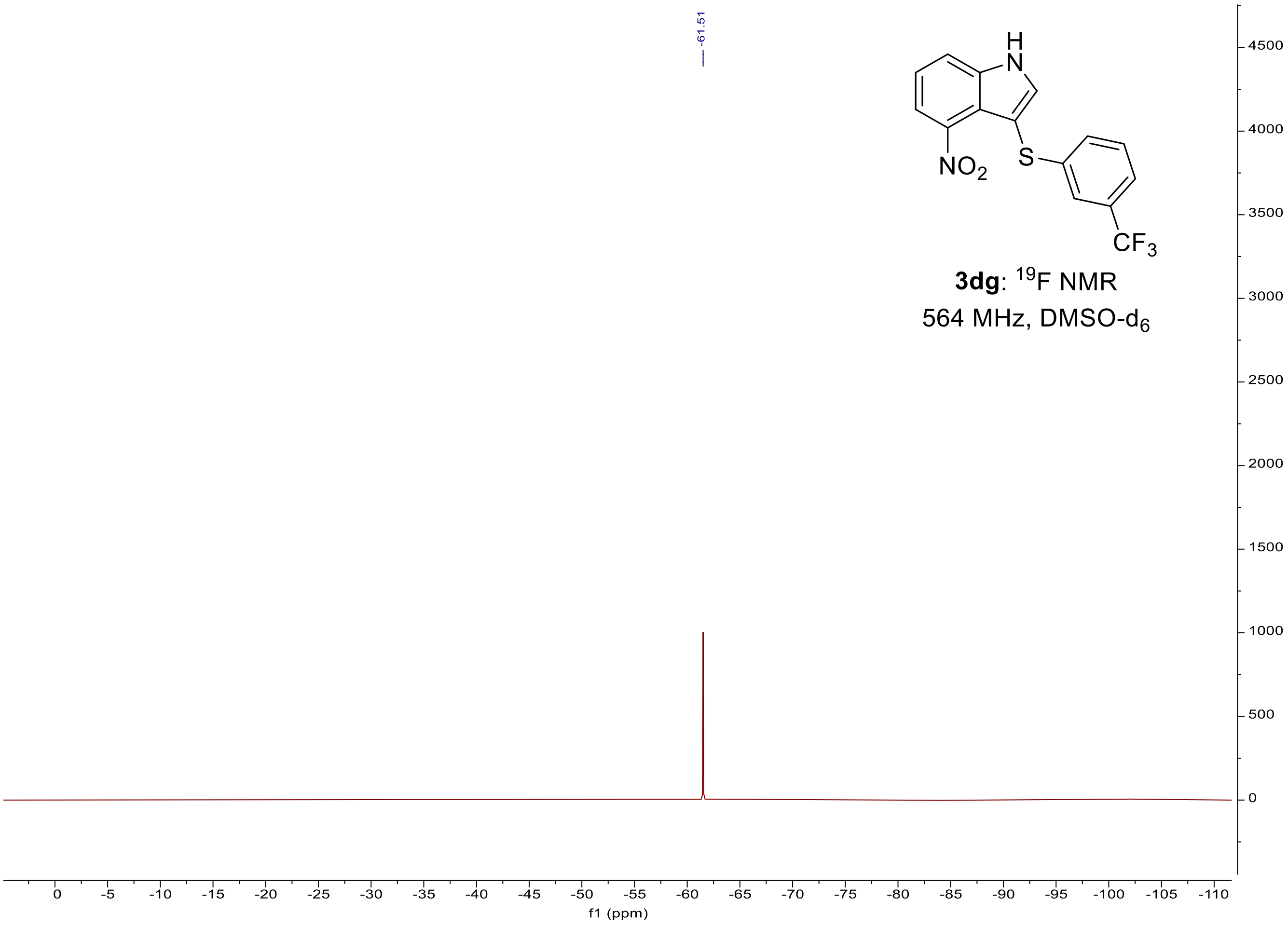
-61.51
H
N
NO2
S
CF3
3dg: 19F NMR
564 MHz, DMSO-d6
4500
4000
3500
3000
2500
2000
1500
1000
500
0
0 -5 -10 -15 -20 -25 -30 -35 -40 -45 -50 -55 -60 -65 -70 -75 -80 -85 -90 -95 -100 -105 -110
f1 (ppm)

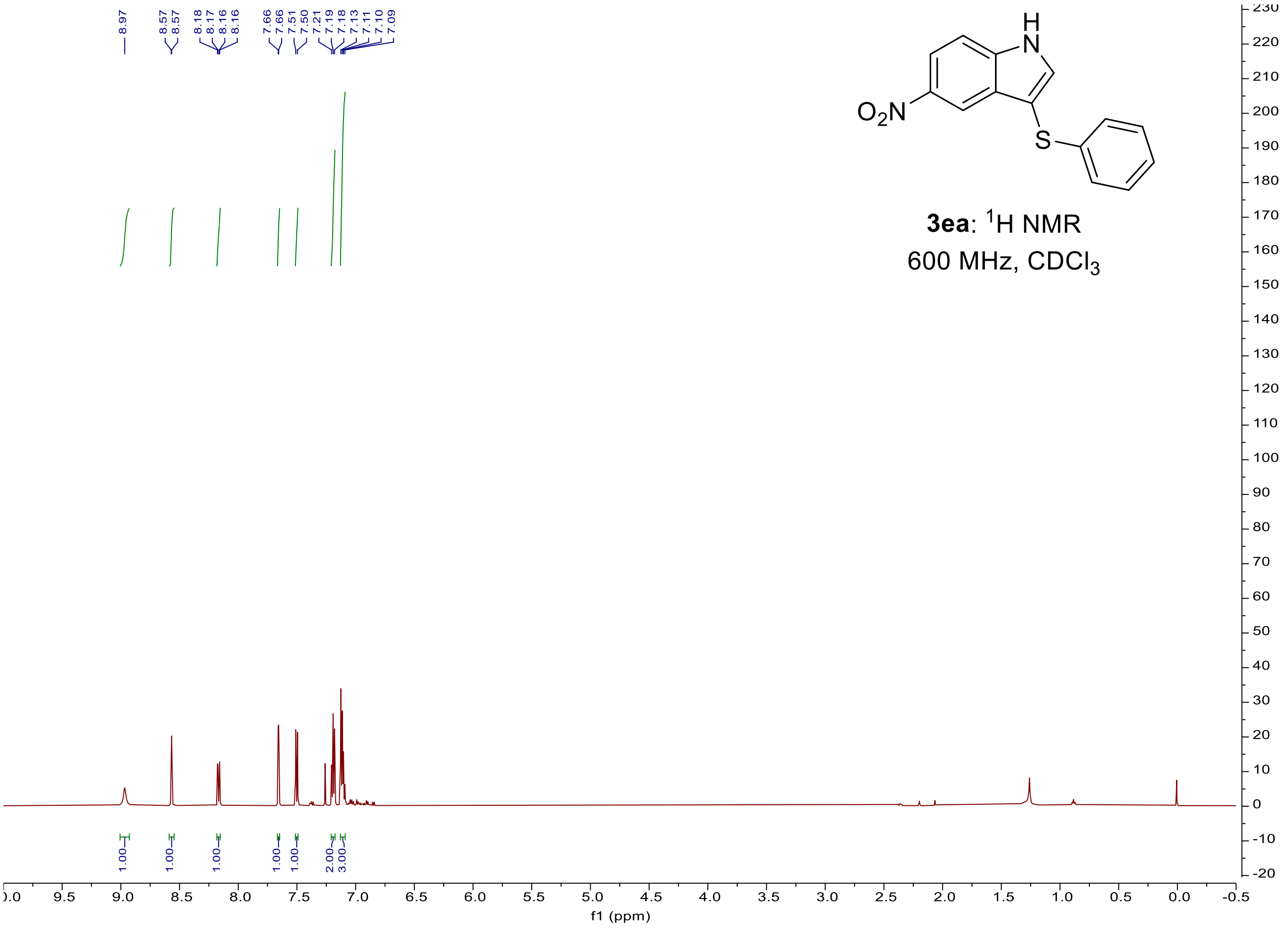
3ea: 1H NMR
600 MHz, CDCl3
O2N
S
H
N
f1 (ppm)

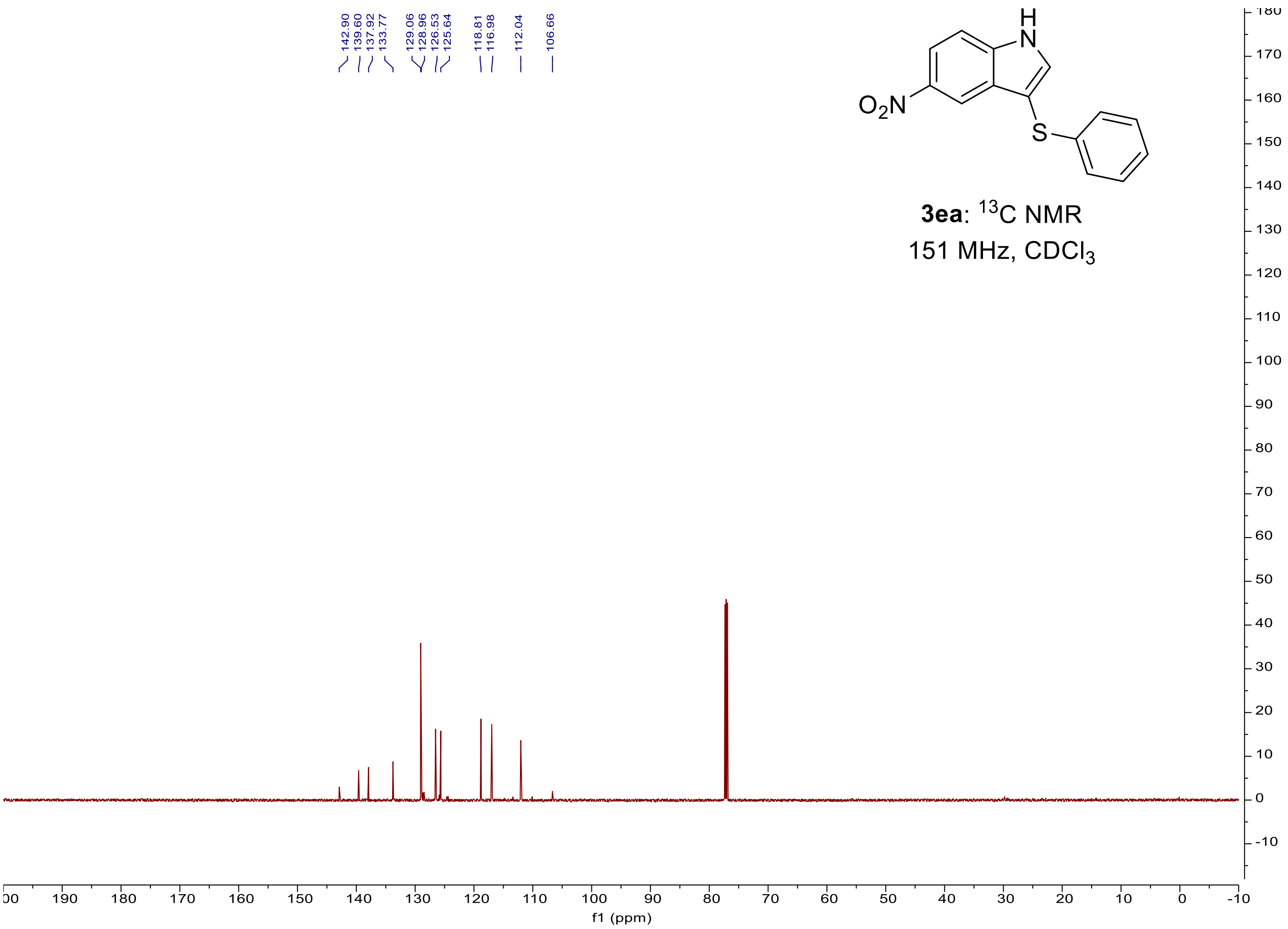
142.90
139.60
137.92
133.77
129.06
128.96
126.53
125.64
118.81
116.98
112.04
106.66
O2N
H
N
S
3ea: 13C NMR
151 MHz, CDCl3
f1 (ppm)

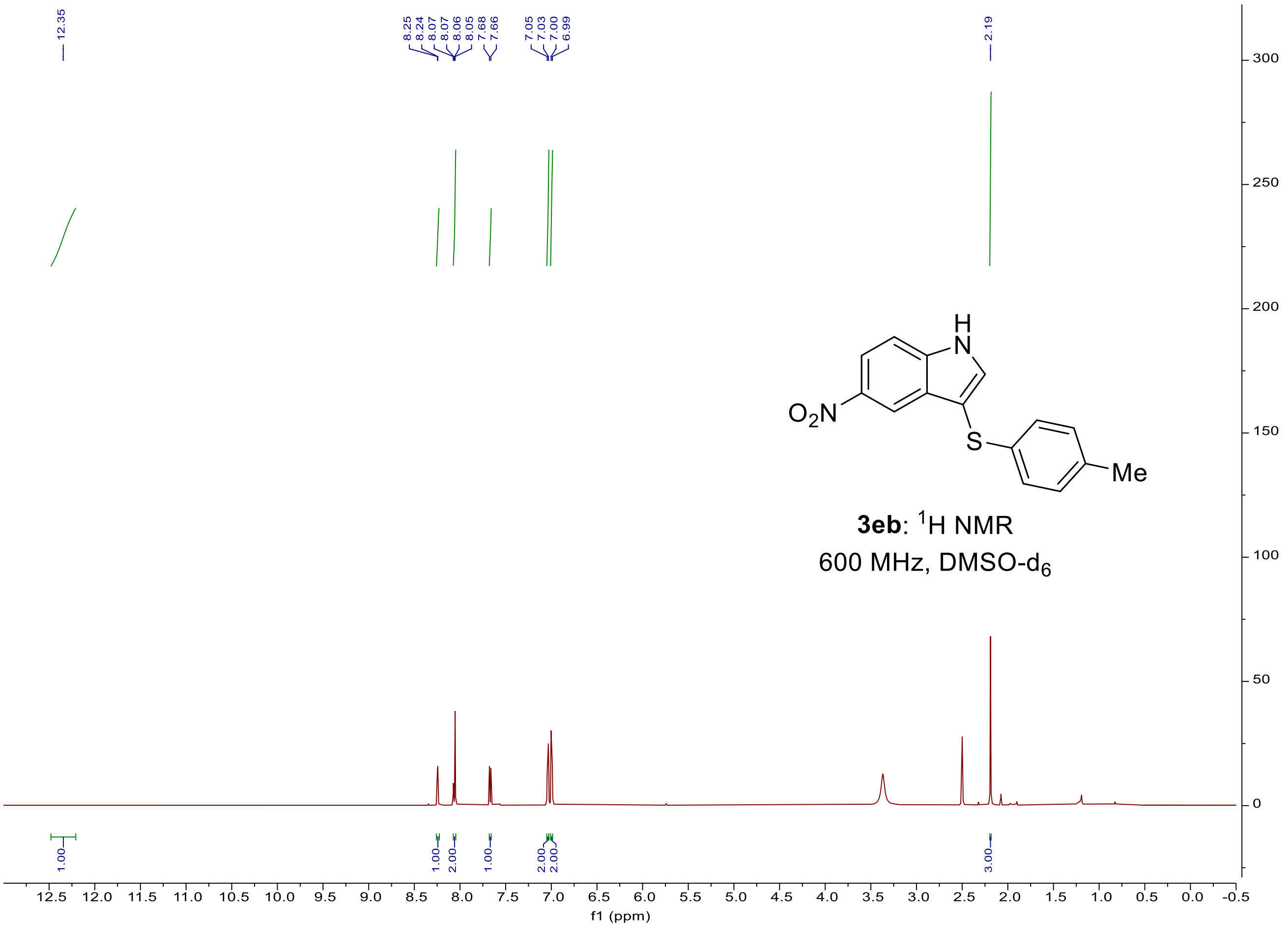
12.35
8.25
8.24
8.07
8.07
8.06
8.05
7.68
7.66
7.05
7.03
7.00
6.99
2.19
O2N
H
N
S
Me
3eb: 1H NMR
600 MHz, DMSO-d6
1.00
1.00
2.00
1.00
2.00
2.00
3.00
f1 (ppm)

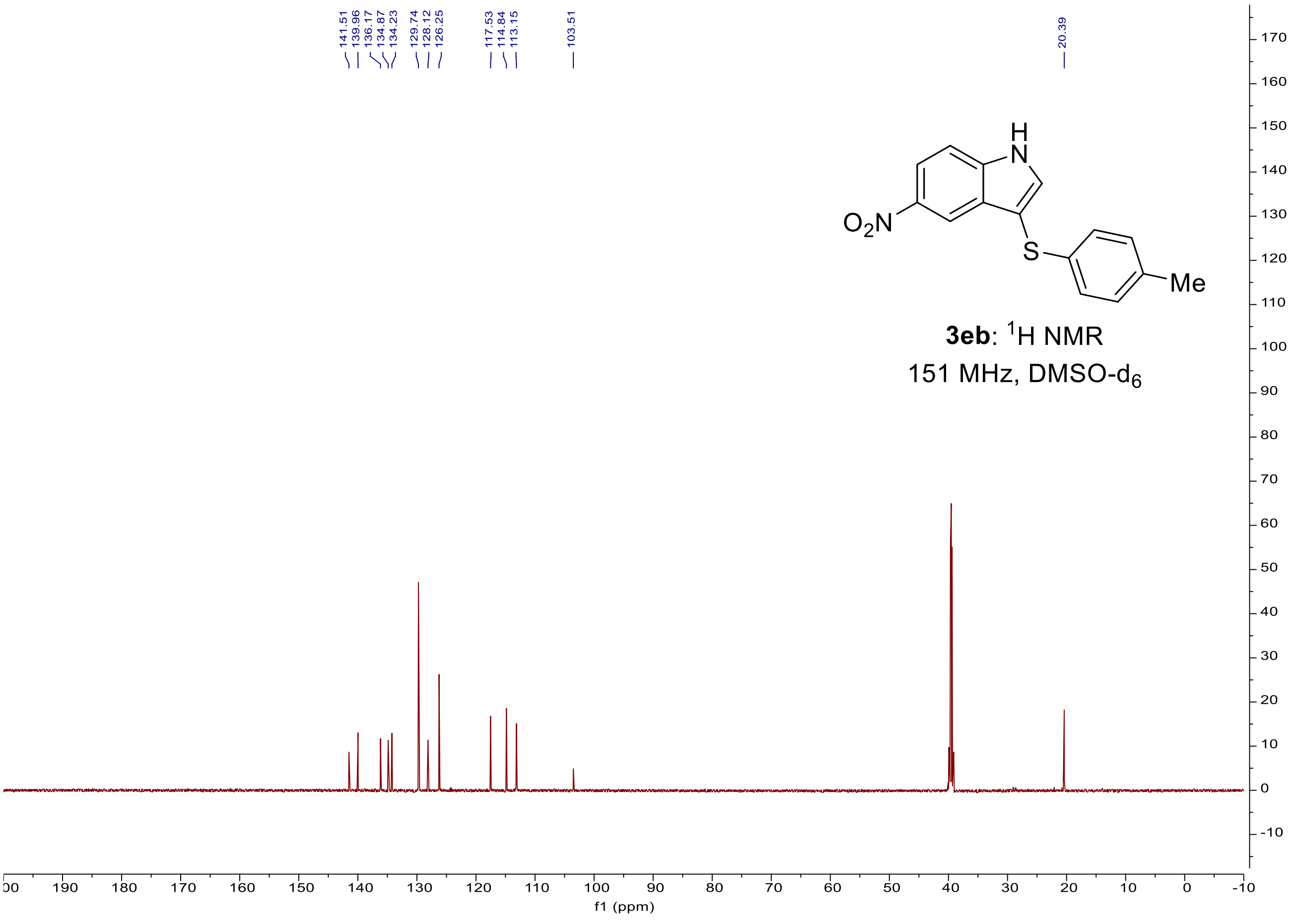
141.51
139.96
136.17
134.87
134.23
129.74
128.12
126.25
117.53
114.84
113.15
103.51
20.39
O2N
H
N
S
Me
3eb: 1H NMR
151 MHz, DMSO-d6
f1 (ppm)

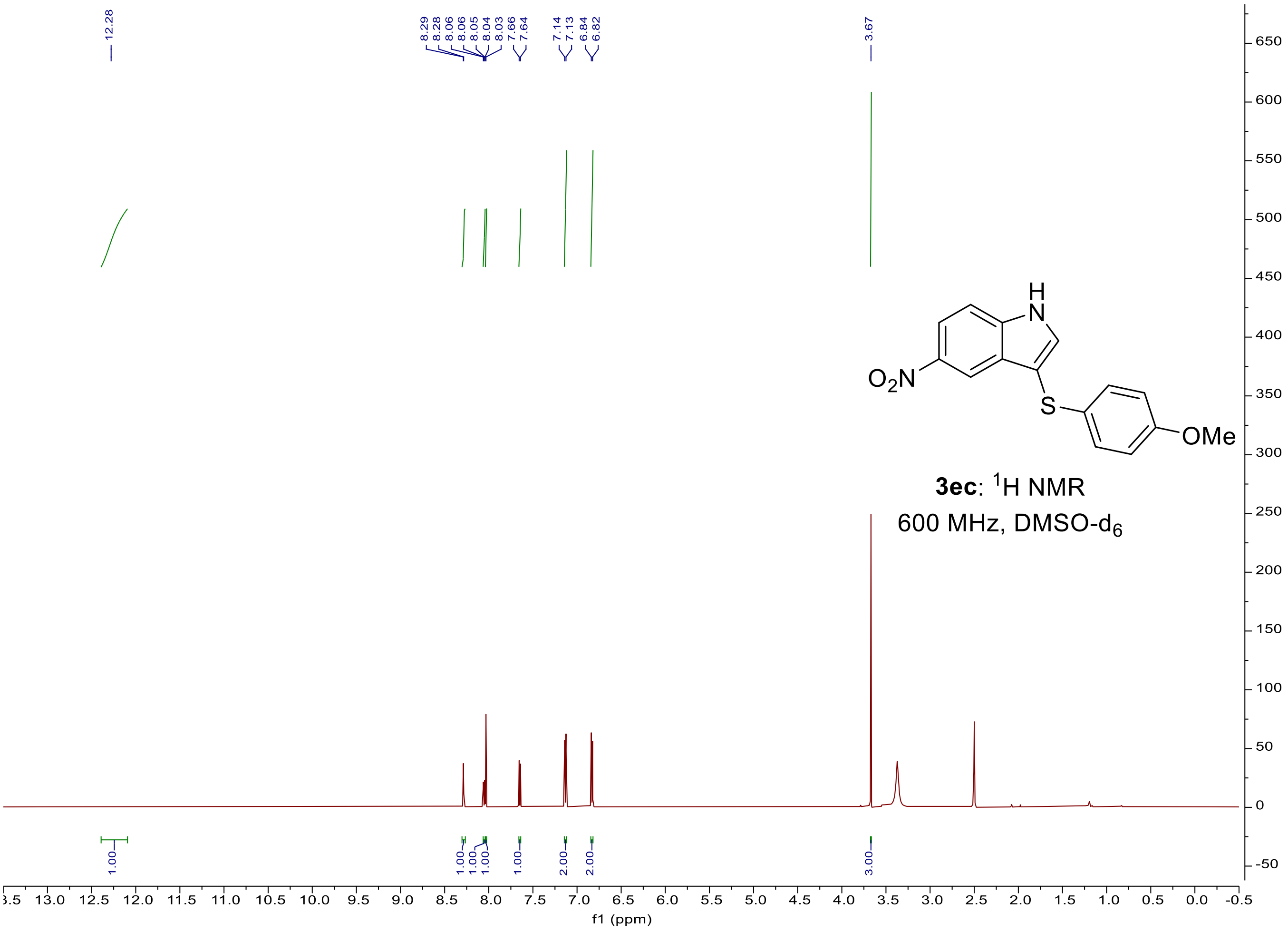
3ec: ¹H NMR
600 MHz, DMSO-d6
O2N
H
N
S
OMe
f1 (ppm)

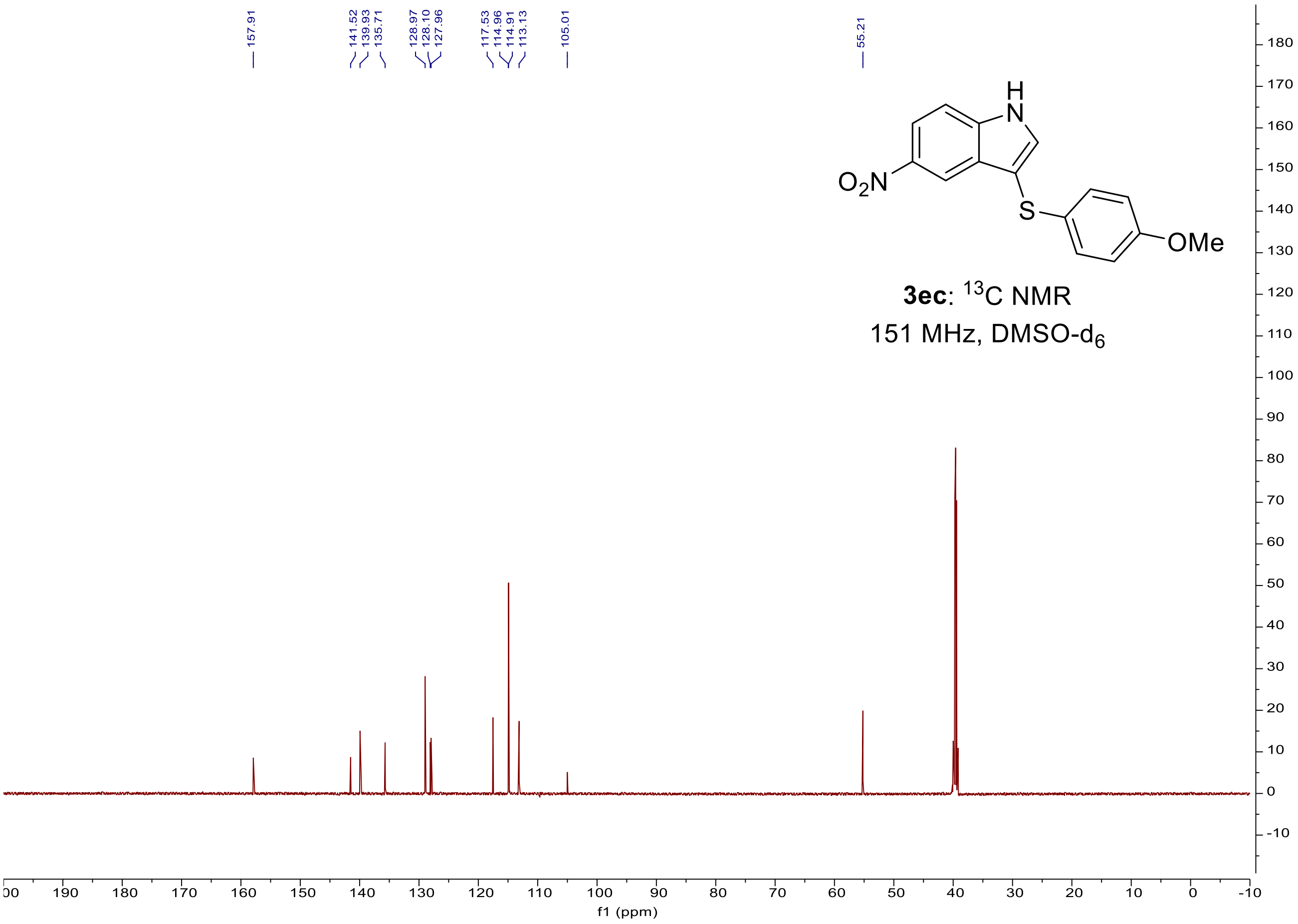
157.91
141.52
139.93
135.71
128.97
128.10
127.96
117.53
114.96
114.91
113.13
105.01
55.21
H
N
O2N
S
OMe
3ec: 13C NMR
151 MHz, DMSO-d6
f1 (ppm)

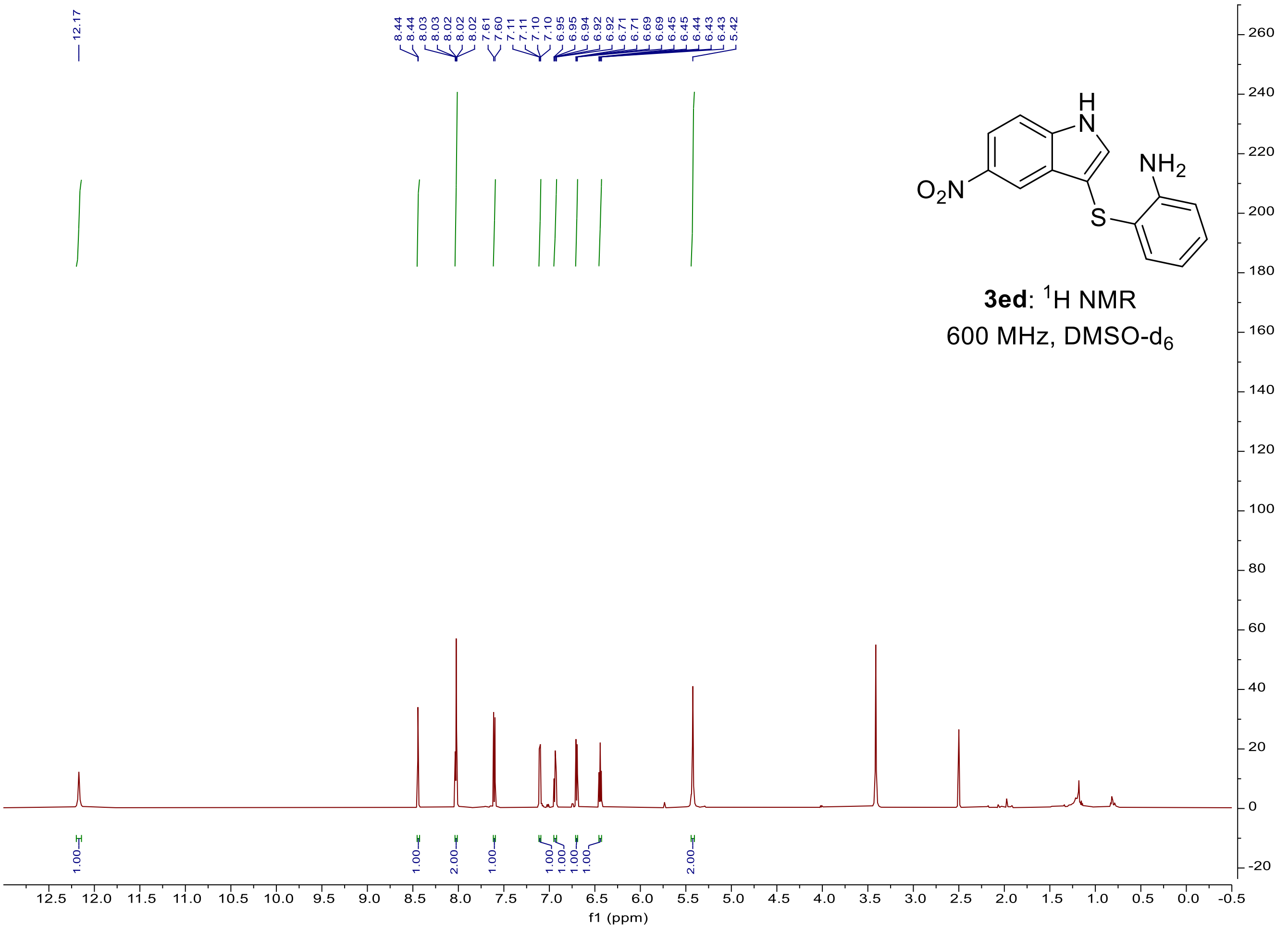
3ed: 1H NMR
600 MHz, DMSO-d6
f1 (ppm)

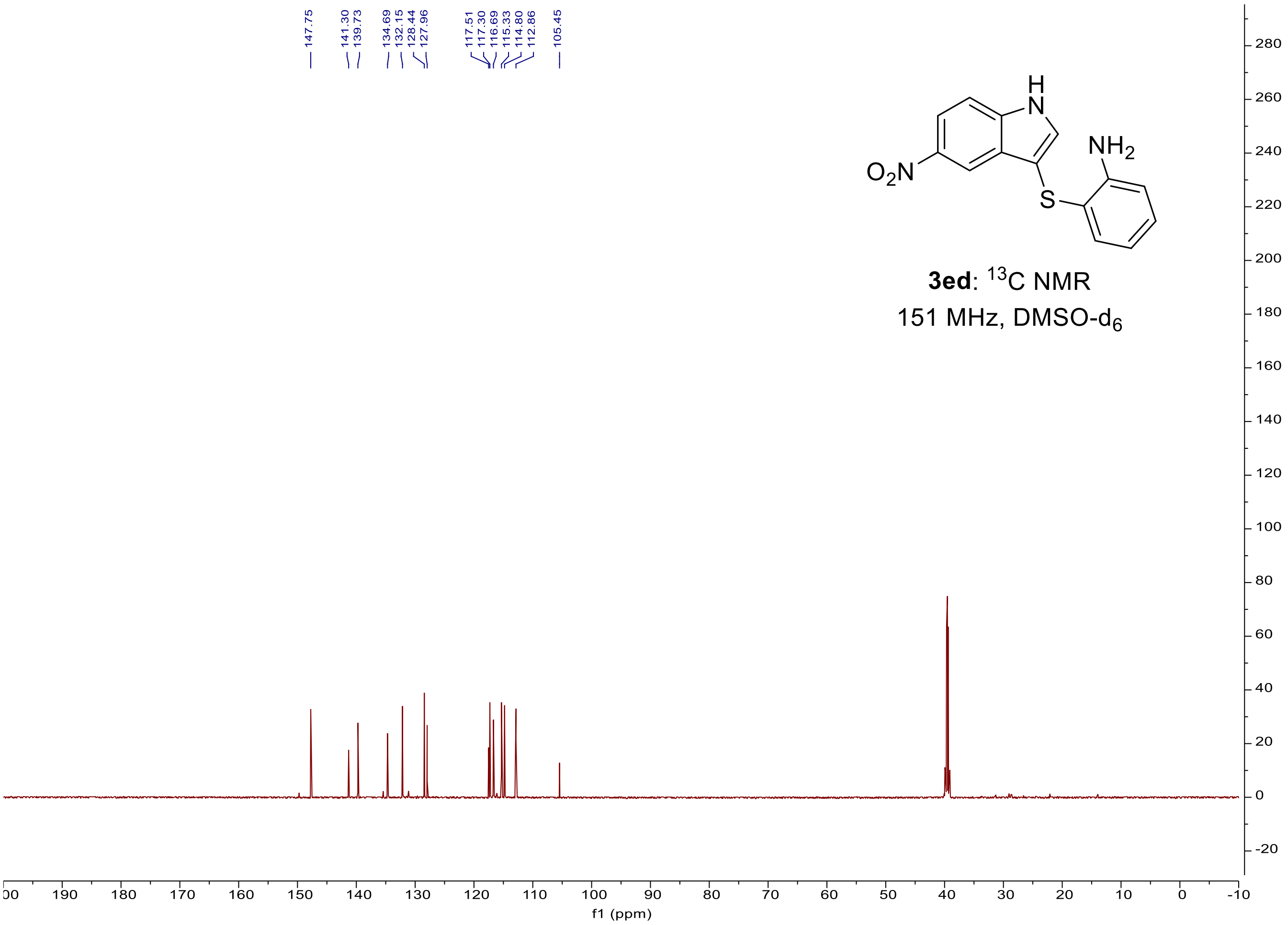
147.75
141.30
139.73
134.69
132.15
128.44
127.96
117.51
117.30
116.69
115.33
114.80
112.86
105.45
3ed: ¹³C NMR
151 MHz, DMSO-d6
f1 (ppm)

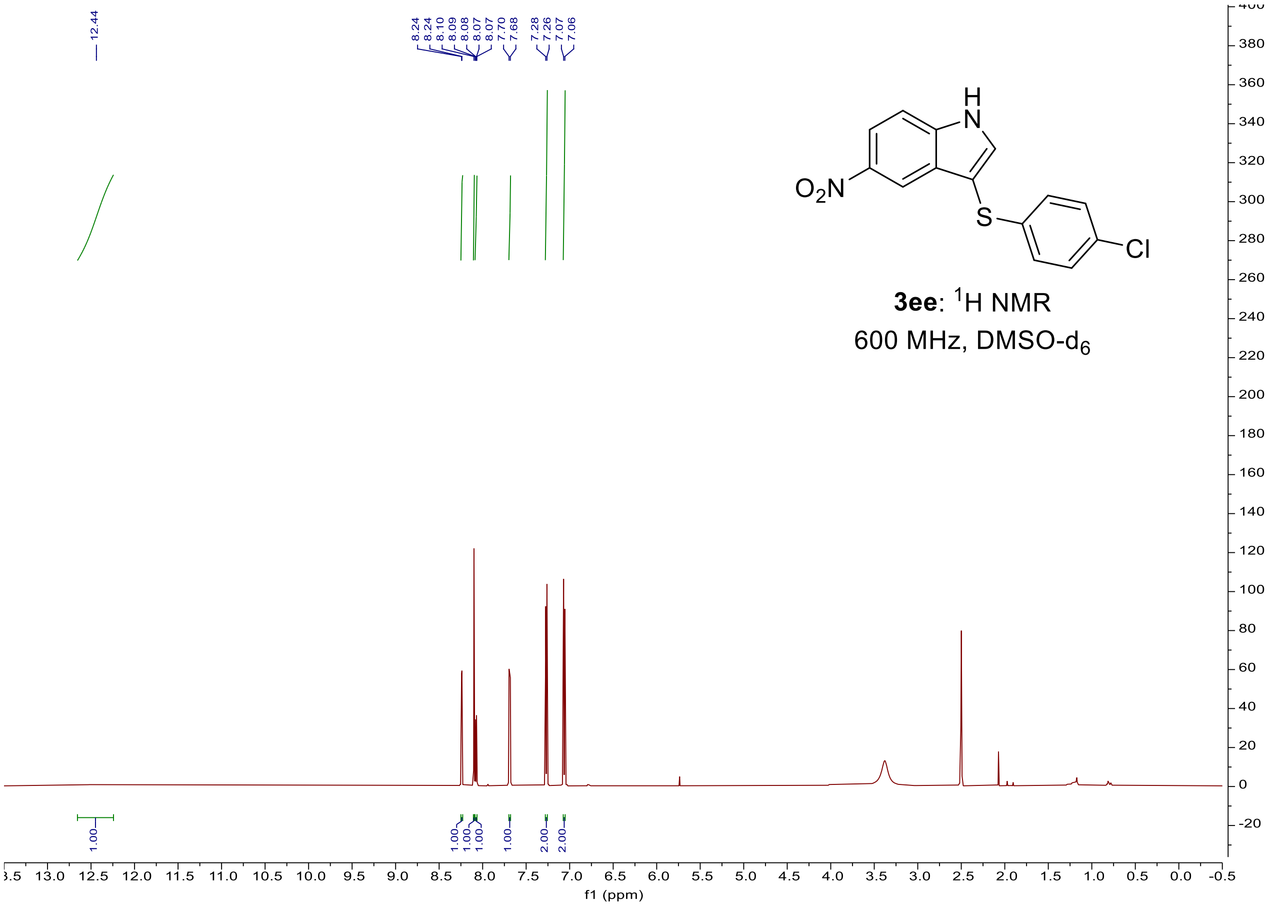
12.44
8.24
8.24
8.10
8.09
8.08
8.07
8.07
7.70
7.68
7.28
7.26
7.07
7.06
3ee: ¹H NMR
600 MHz, DMSO-d6
1.00
1.00
1.00
1.00
1.00
2.00
2.00
f1 (ppm)

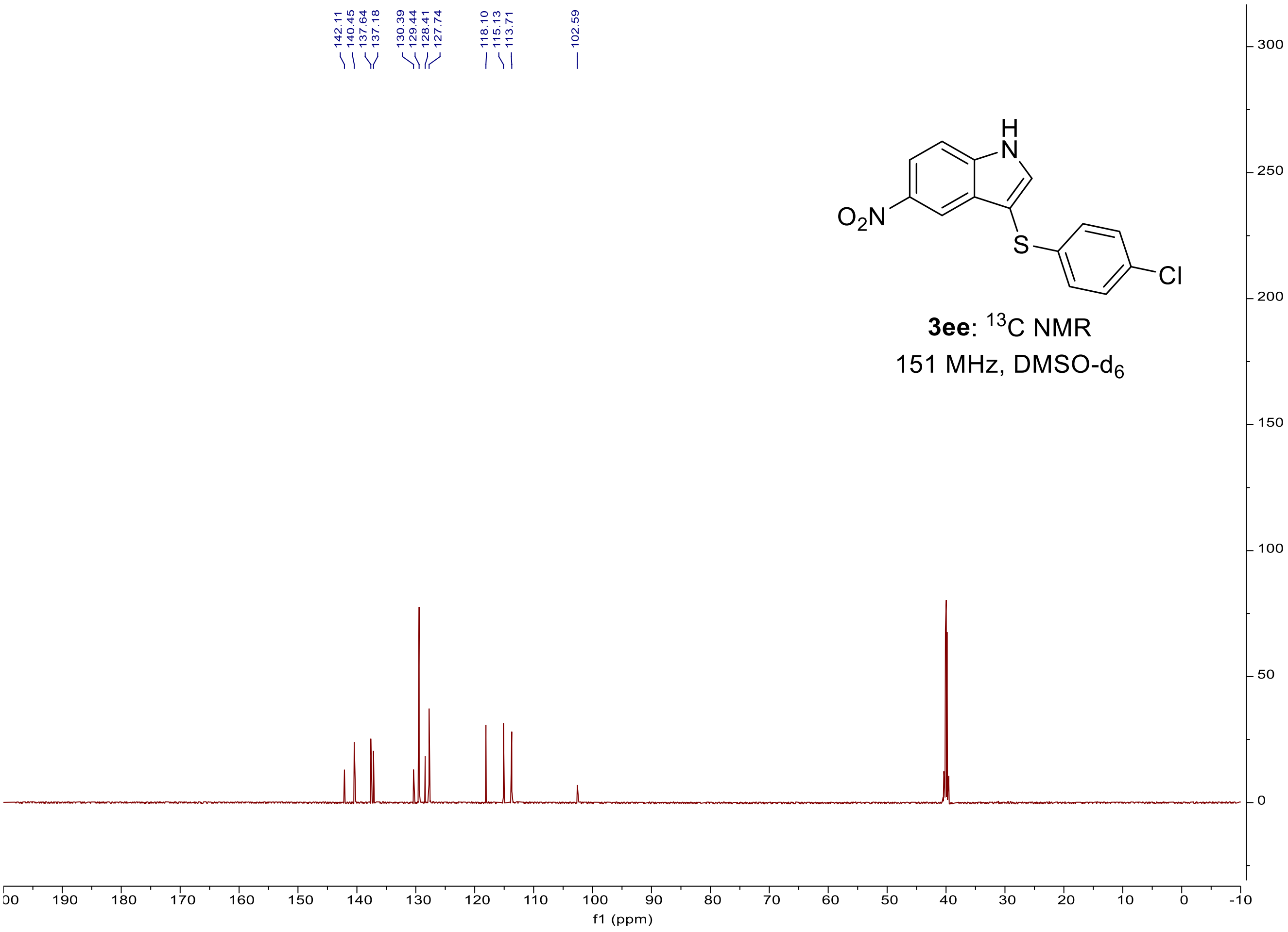
142.11
140.45
137.64
137.18
130.39
129.44
128.41
127.74
118.10
115.13
113.71
102.59
O2N
H
N
S
Cl
3ee: 13C NMR
151 MHz, DMSO-d6
f1 (ppm)

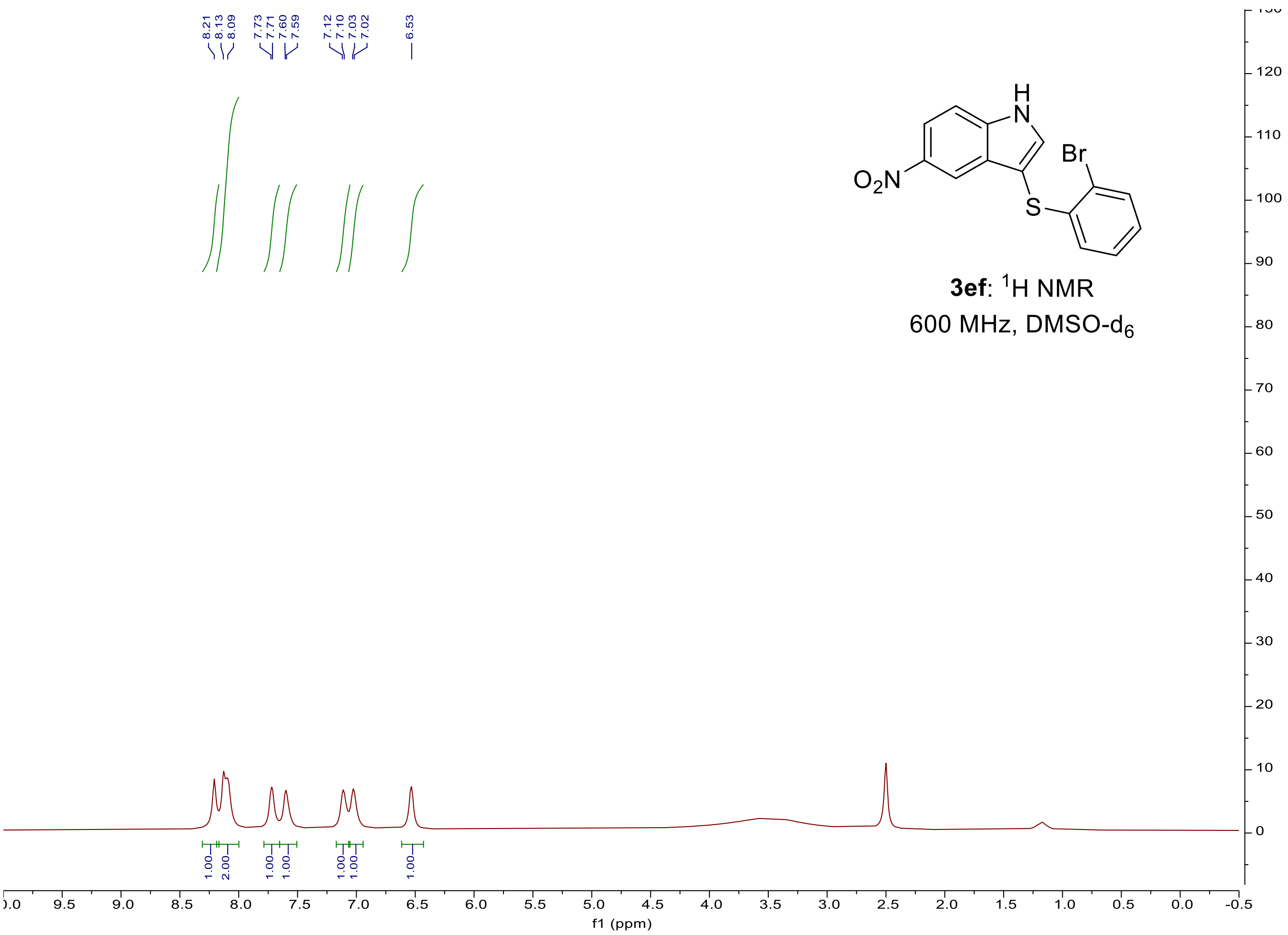

8.21
8.13
8.09
7.73
7.71
7.60
7.59
7.12
7.10
7.03
7.02
6.53
H
N
Br
O2N
S
3ef: 1H NMR
600 MHz, DMSO-d6
1.00
2.00
1.00
1.00
1.00
1.00
1.00
f1 (ppm)

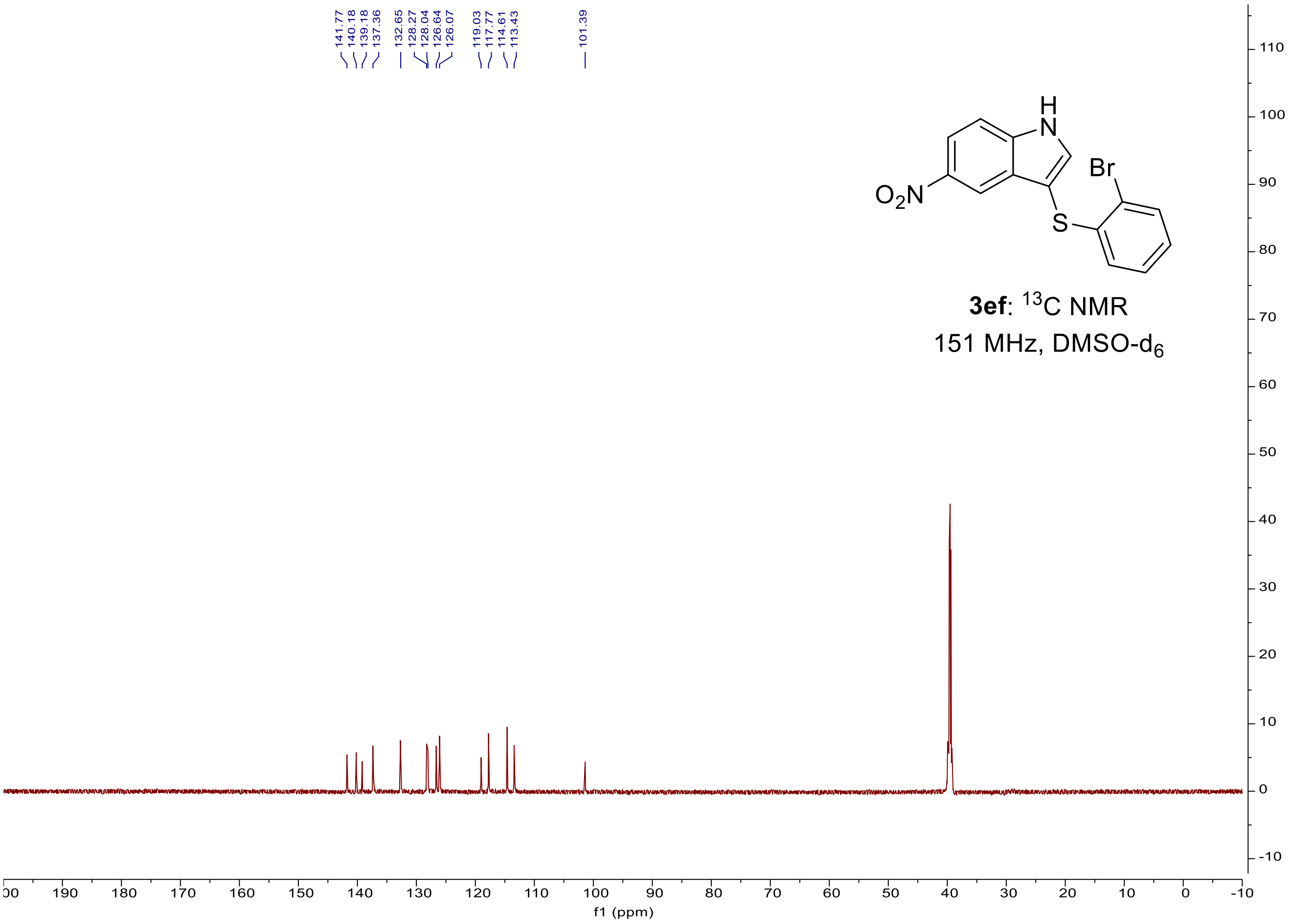
141.77
140.18
139.18
137.36
132.65
128.27
128.04
126.64
126.07
119.03
117.77
114.61
113.43
101.39
H
N
Br
O2N
S
3ef: 13C NMR
151 MHz, DMSO-d6
f1 (ppm)

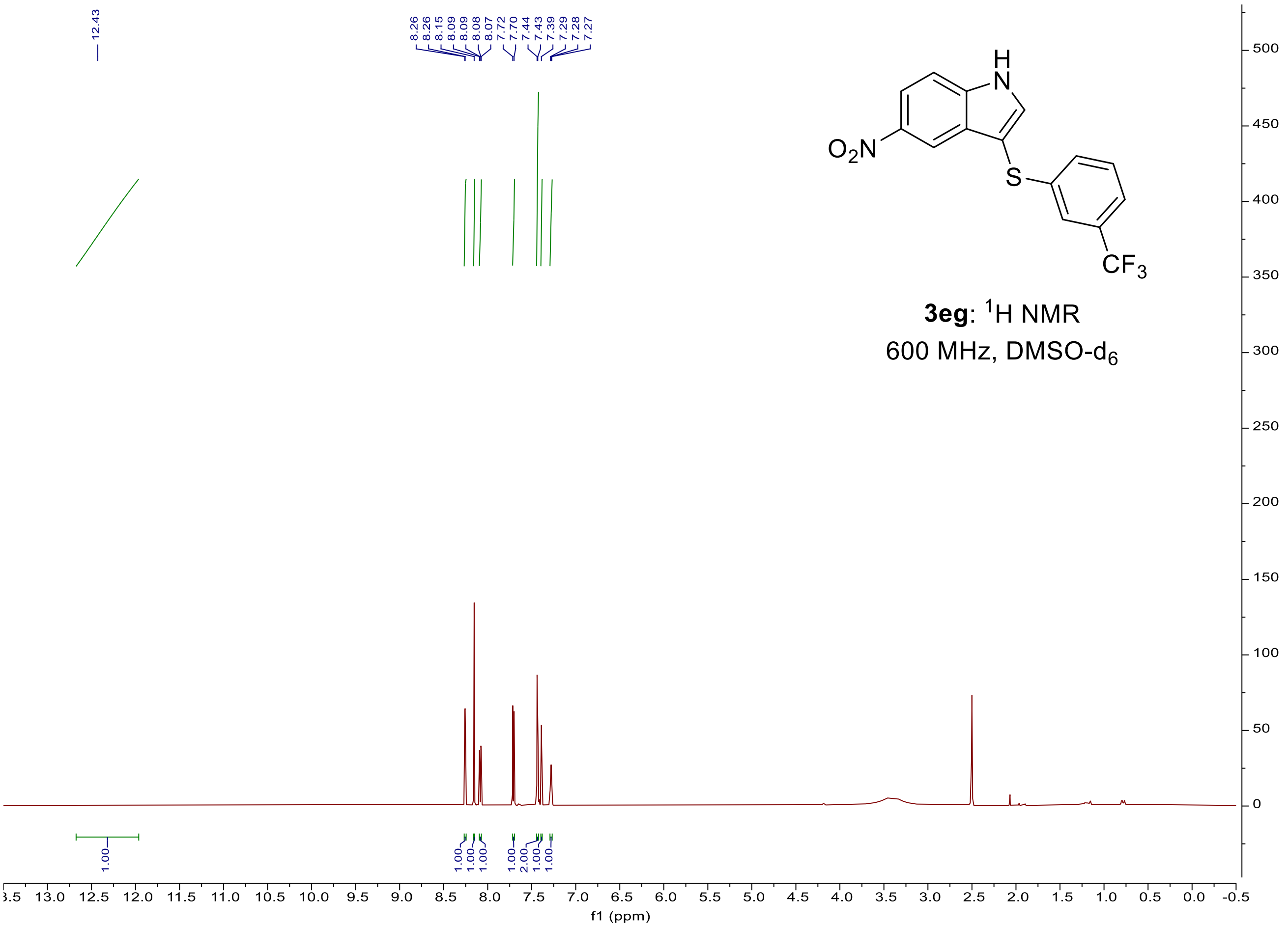

3eg: ^{1}H NMR

600 MHz, DMSO-d_6

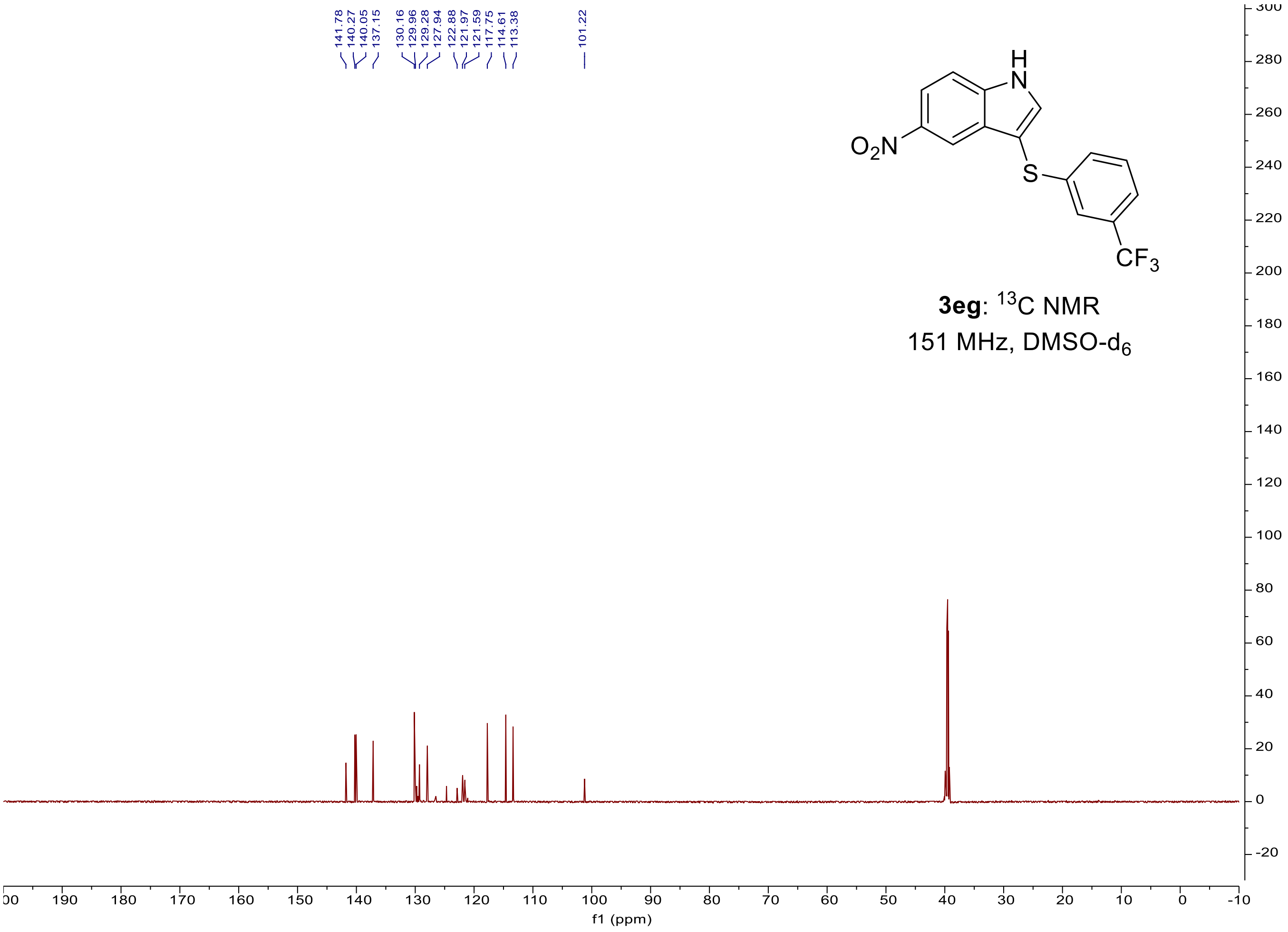
141.78
140.27
140.05
137.15
130.16
129.96
129.28
127.94
122.88
121.97
121.59
117.75
114.61
113.38
101.22
O2N
H
N
S
CF3
3eg: 13C NMR
151 MHz, DMSO-d6
f1 (ppm)

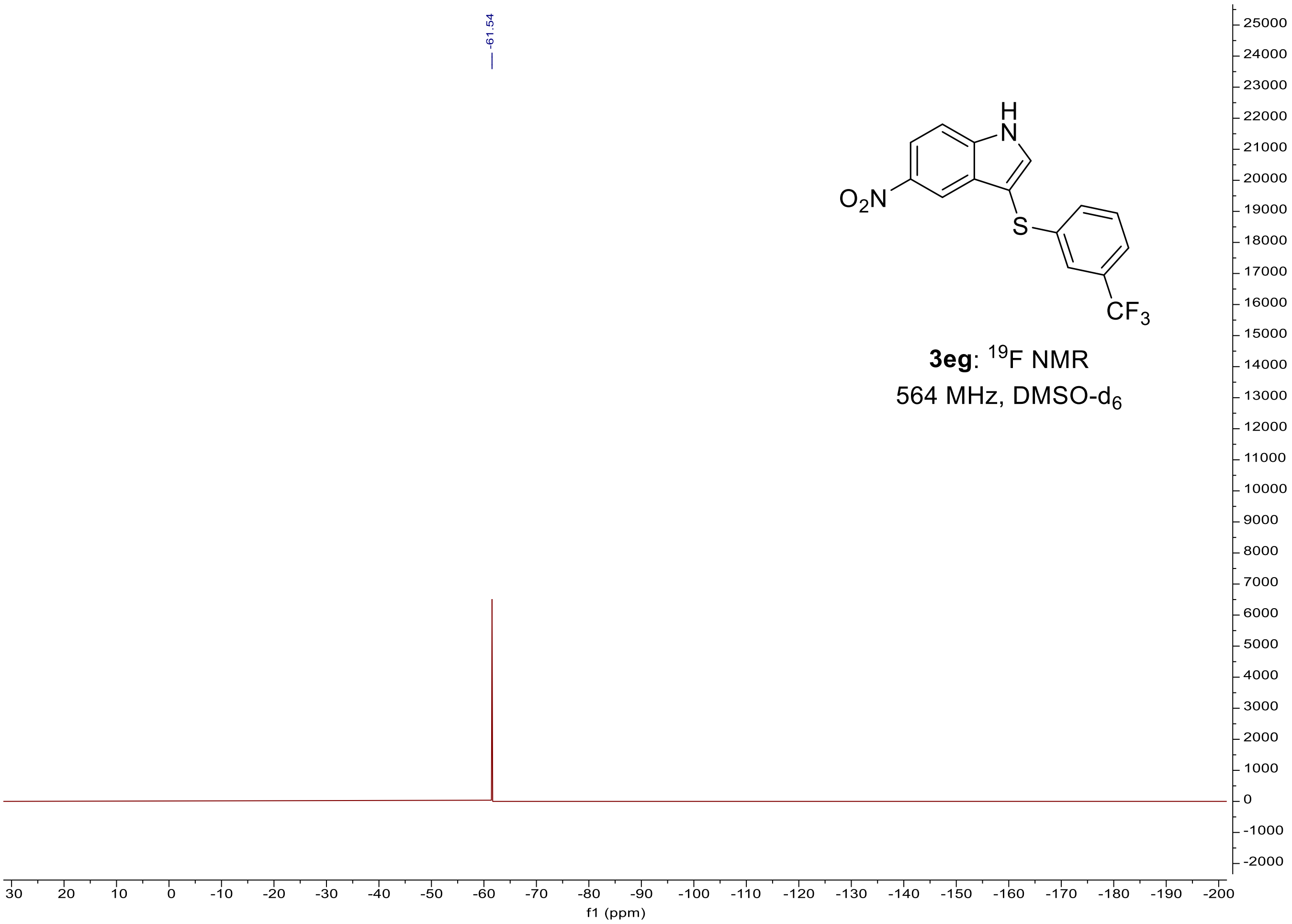
-61.54
3eg: ^{19}F NMR
564 MHz, DMSO-d$_6$
O_2N
H
N
S
CF_3
f1 (ppm)

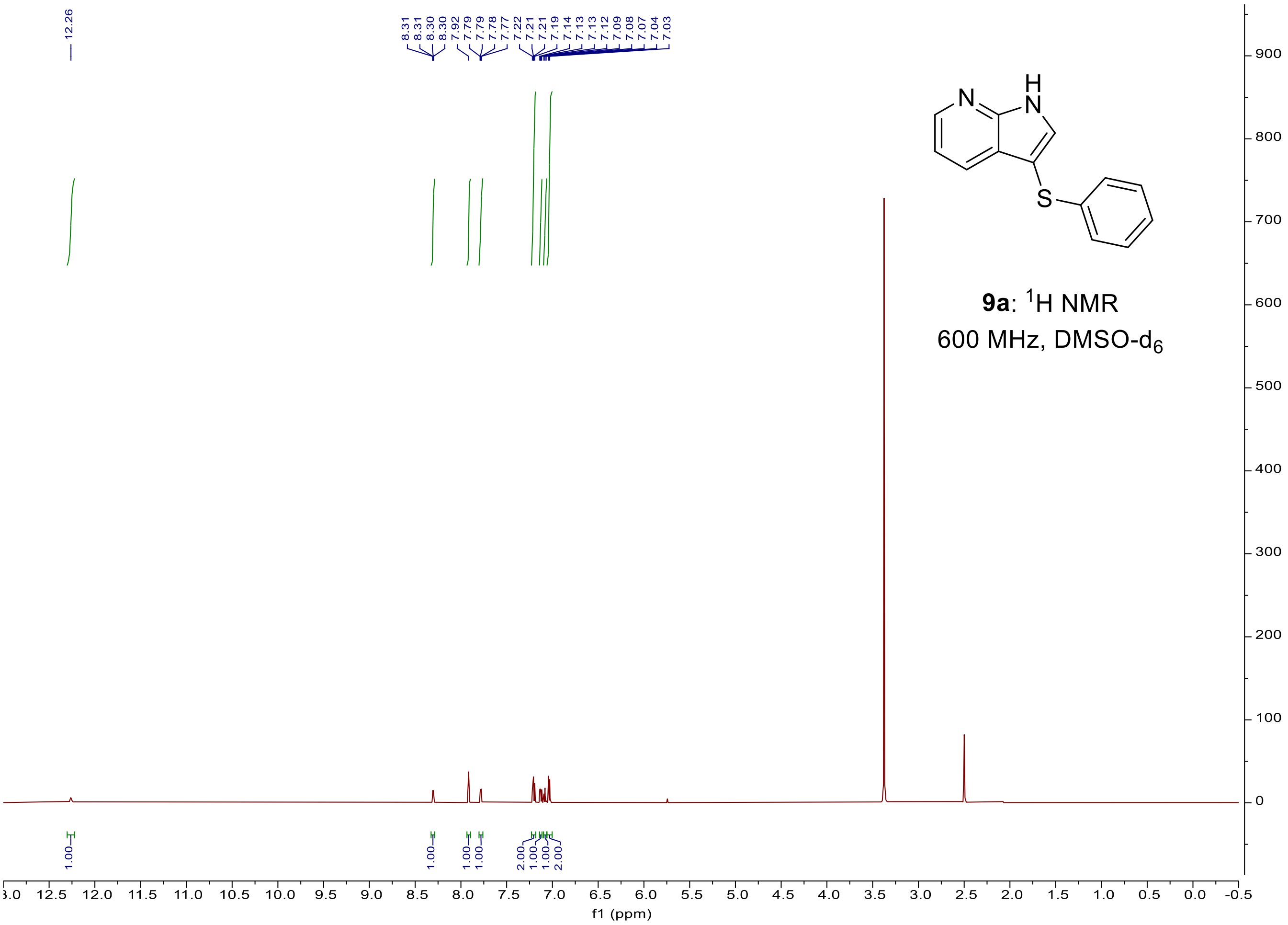
9a: 1H NMR
600 MHz, DMSO-d6
f1 (ppm)

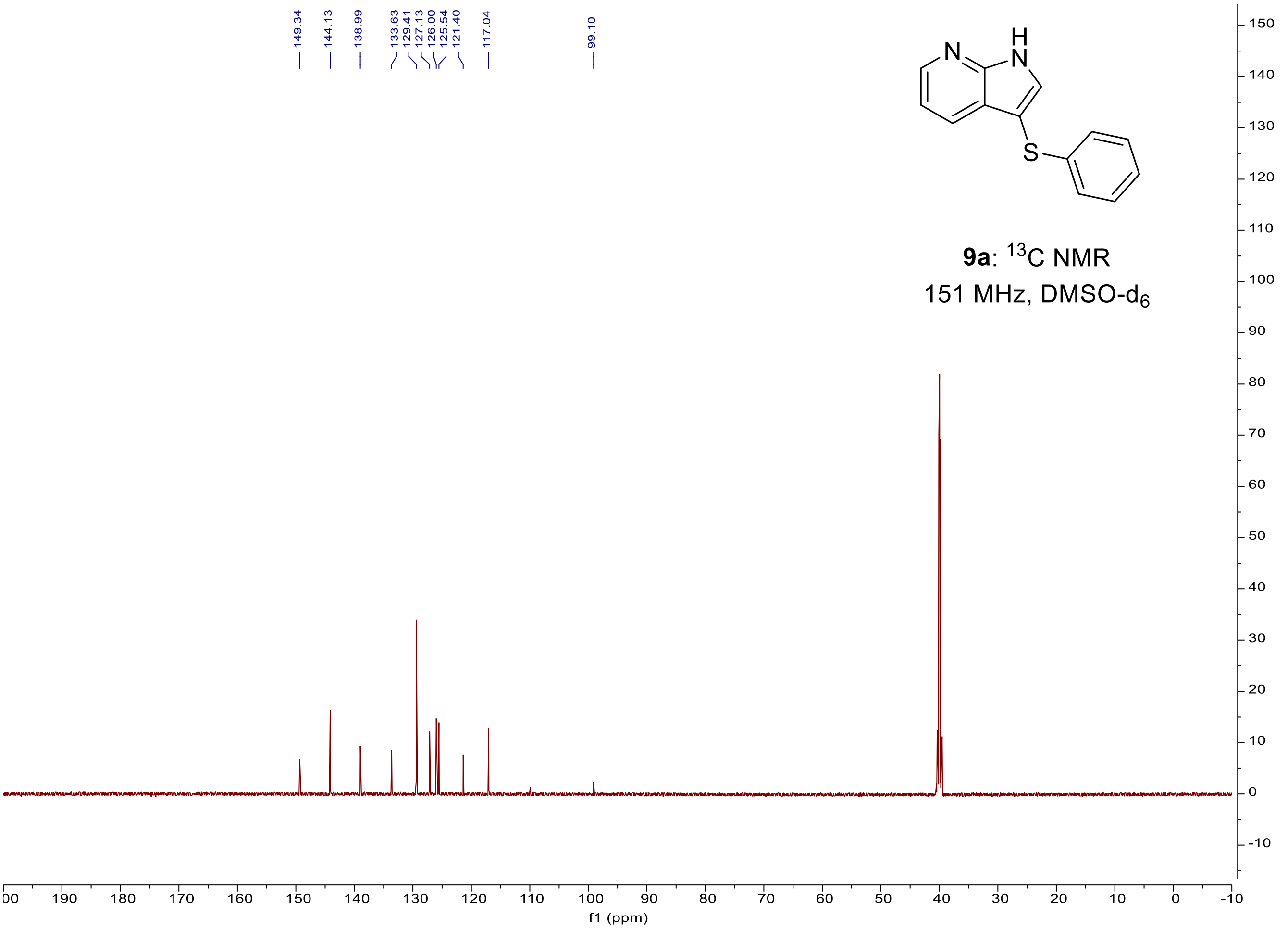
149.34
144.13
138.99
133.63
129.41
127.13
126.00
125.54
121.40
117.04
99.10
N
H
N
S
9a: 13C NMR
151 MHz, DMSO-d6
f1 (ppm)

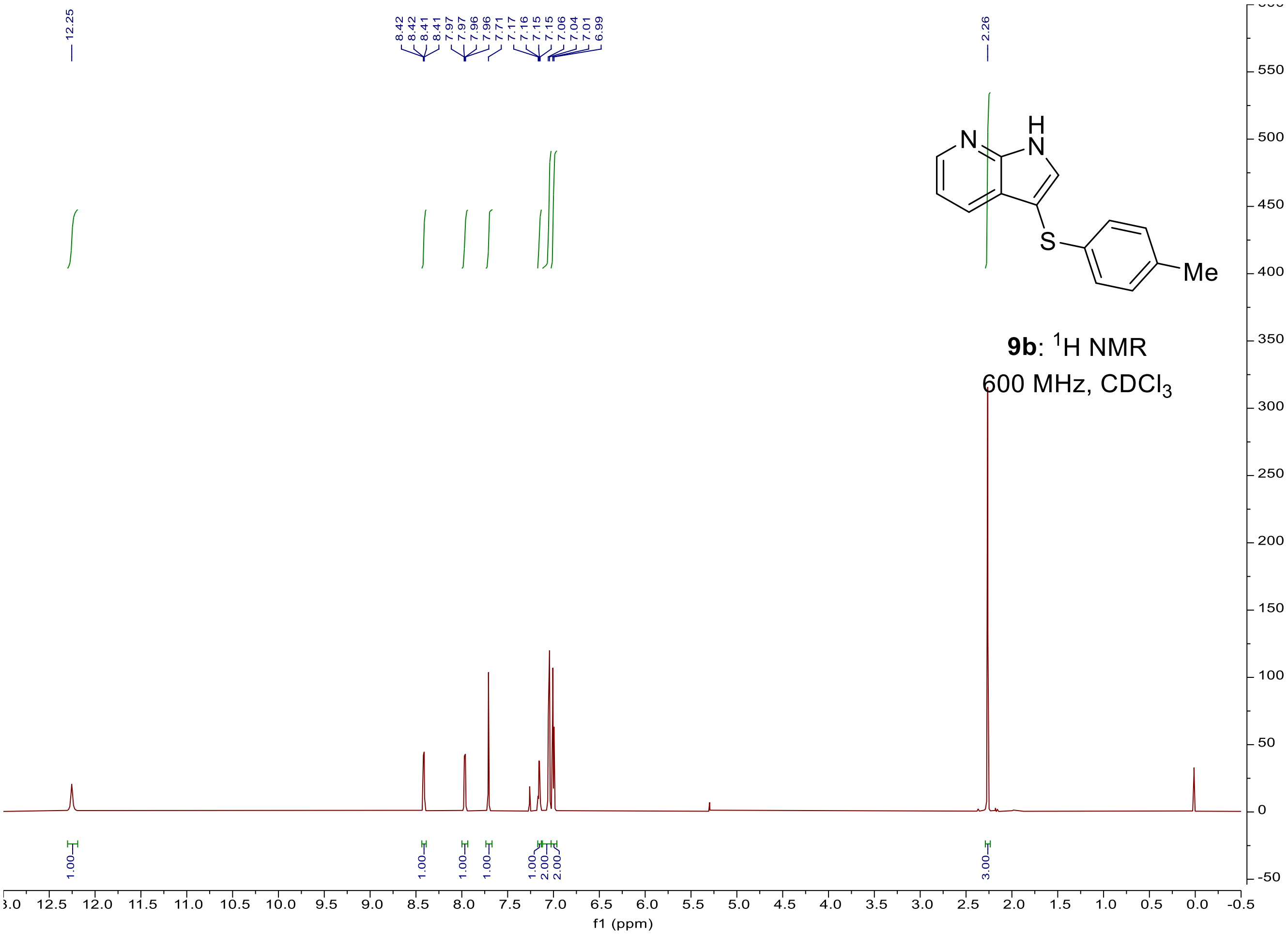

N
H
N
S
Me
9b: ¹H NMR
600 MHz, CDCl3
f1 (ppm)

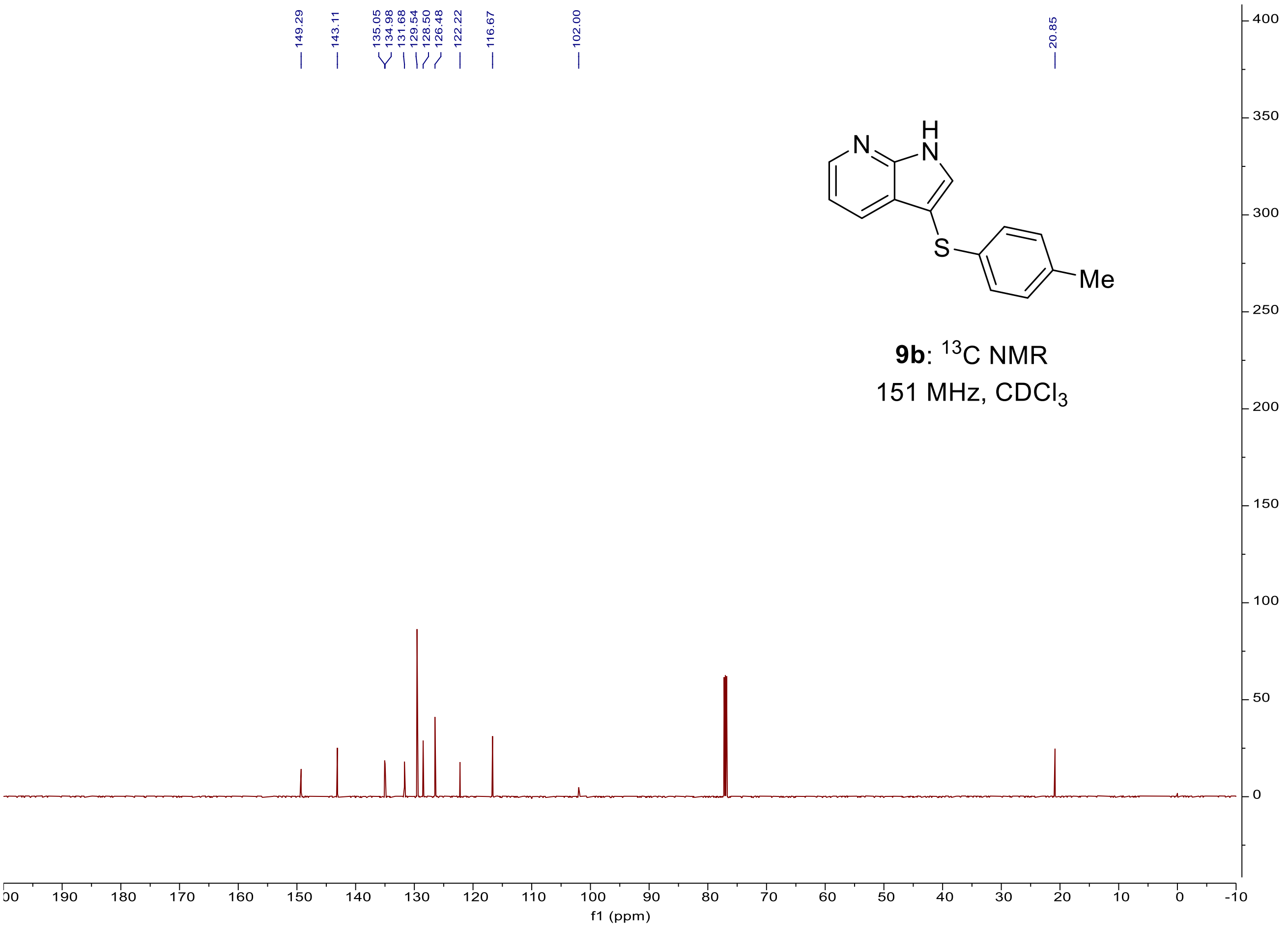
149.29
143.11
135.05
134.98
131.68
129.54
128.50
126.48
122.22
116.67
102.00
20.85
9b: 13C NMR
151 MHz, CDCl3
f1 (ppm)

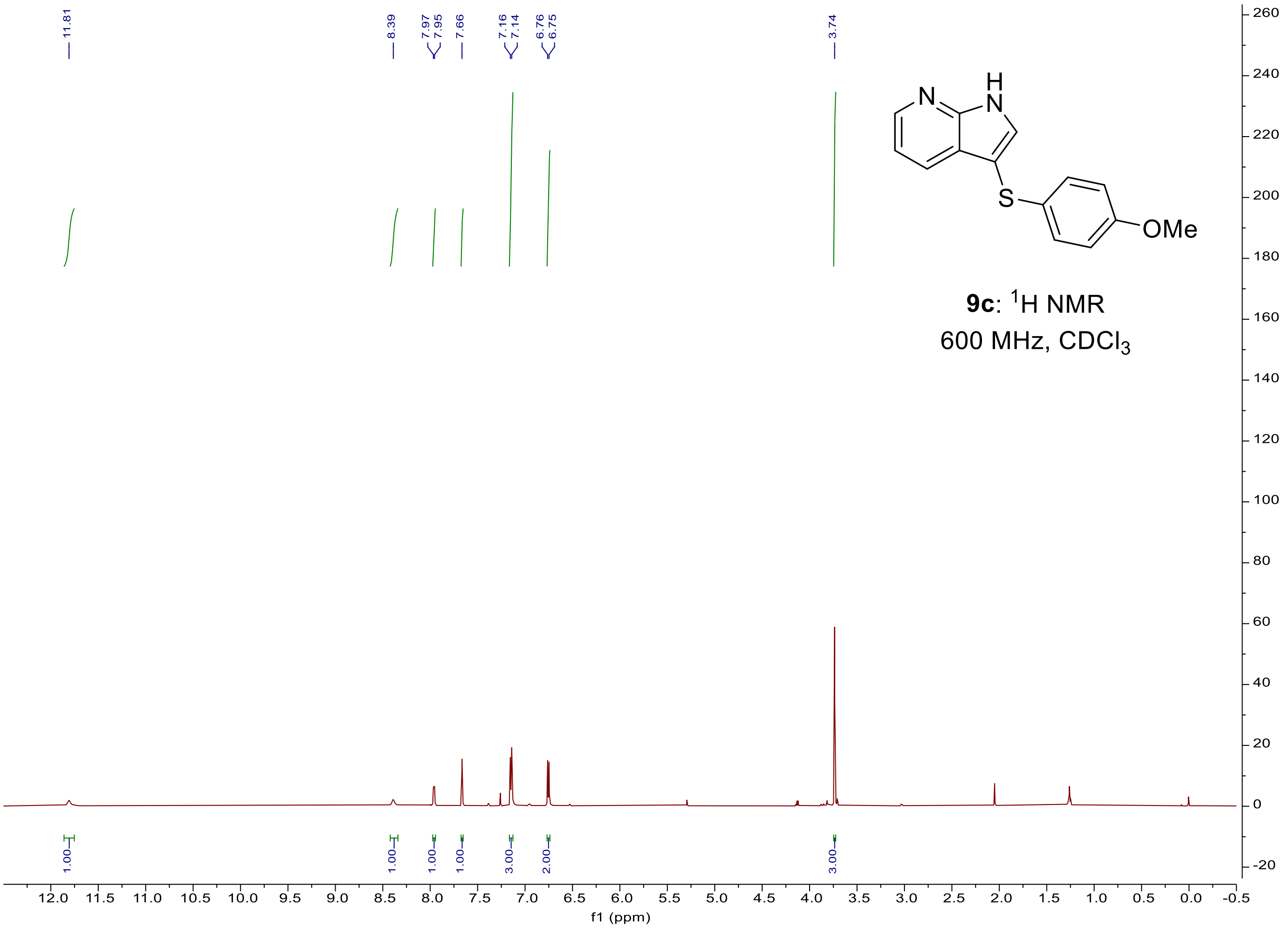
11.81
8.39
7.97
7.95
7.66
7.16
7.14
6.76
6.75
3.74
1.00
1.00
1.00
1.00
3.00
2.00
3.00
f1 (ppm)
OMe
S
N
H
9c: 1H NMR
600 MHz, CDCl3

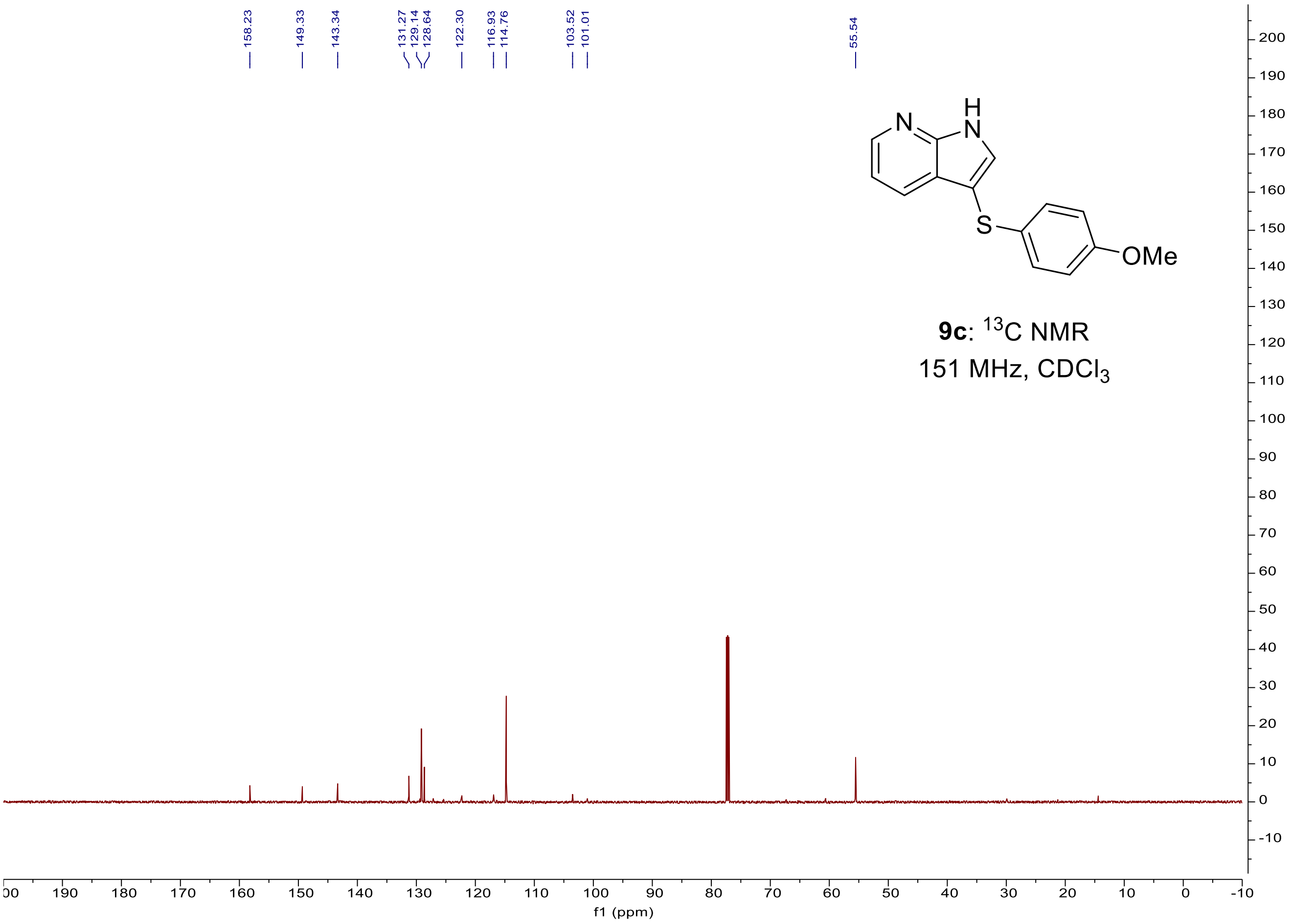

9c: ^{13}C NMR
151 MHz, $CDCl_3$

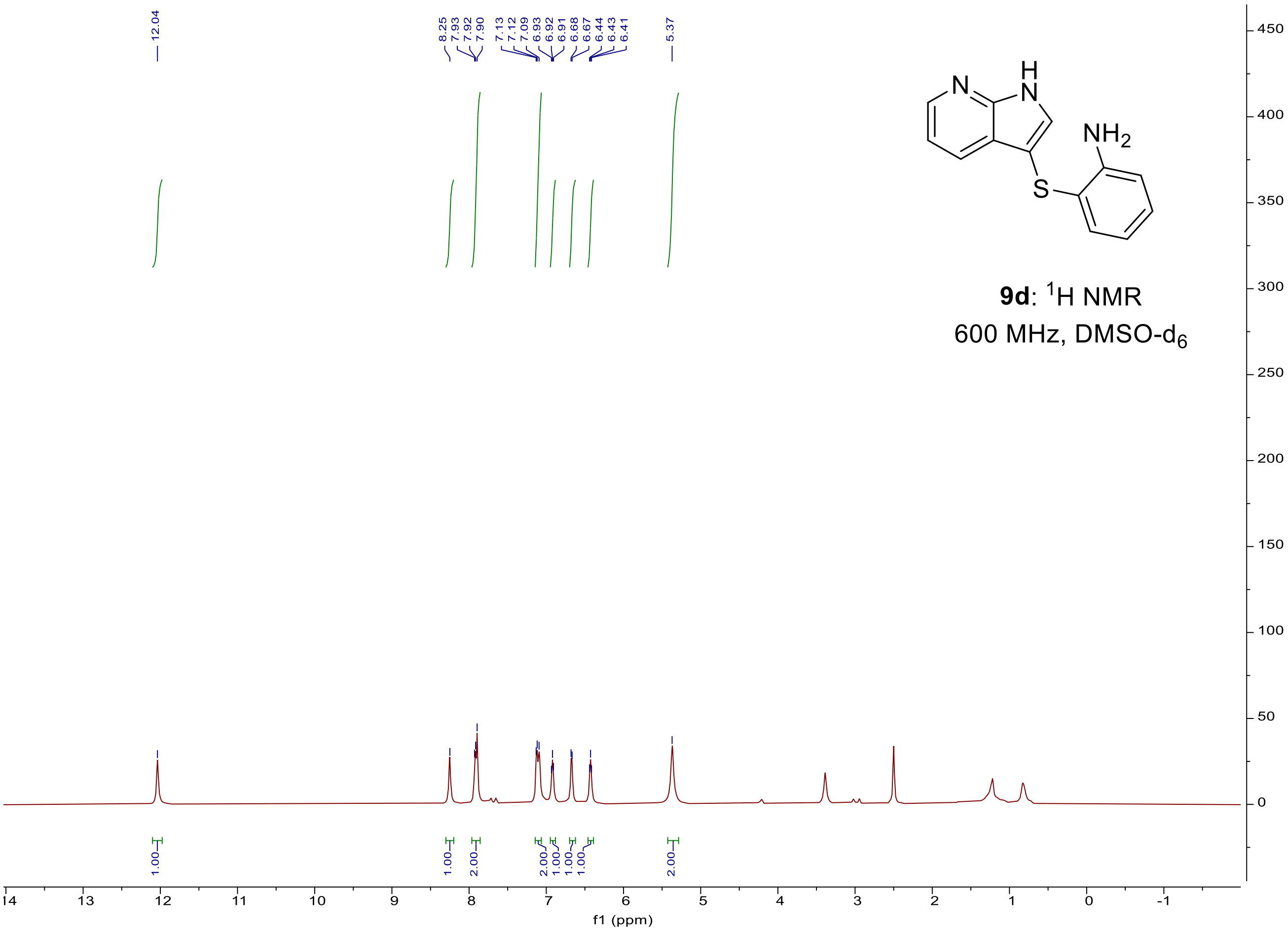
9d: ¹H NMR
600 MHz, DMSO-d6
NH2
N
H
S
12.04
8.25
7.93
7.92
7.90
7.13
7.12
7.09
6.93
6.92
6.91
6.68
6.67
6.44
6.43
6.41
5.37
1.00
2.00
2.00
1.00
1.00
1.00
2.00
f1 (ppm)

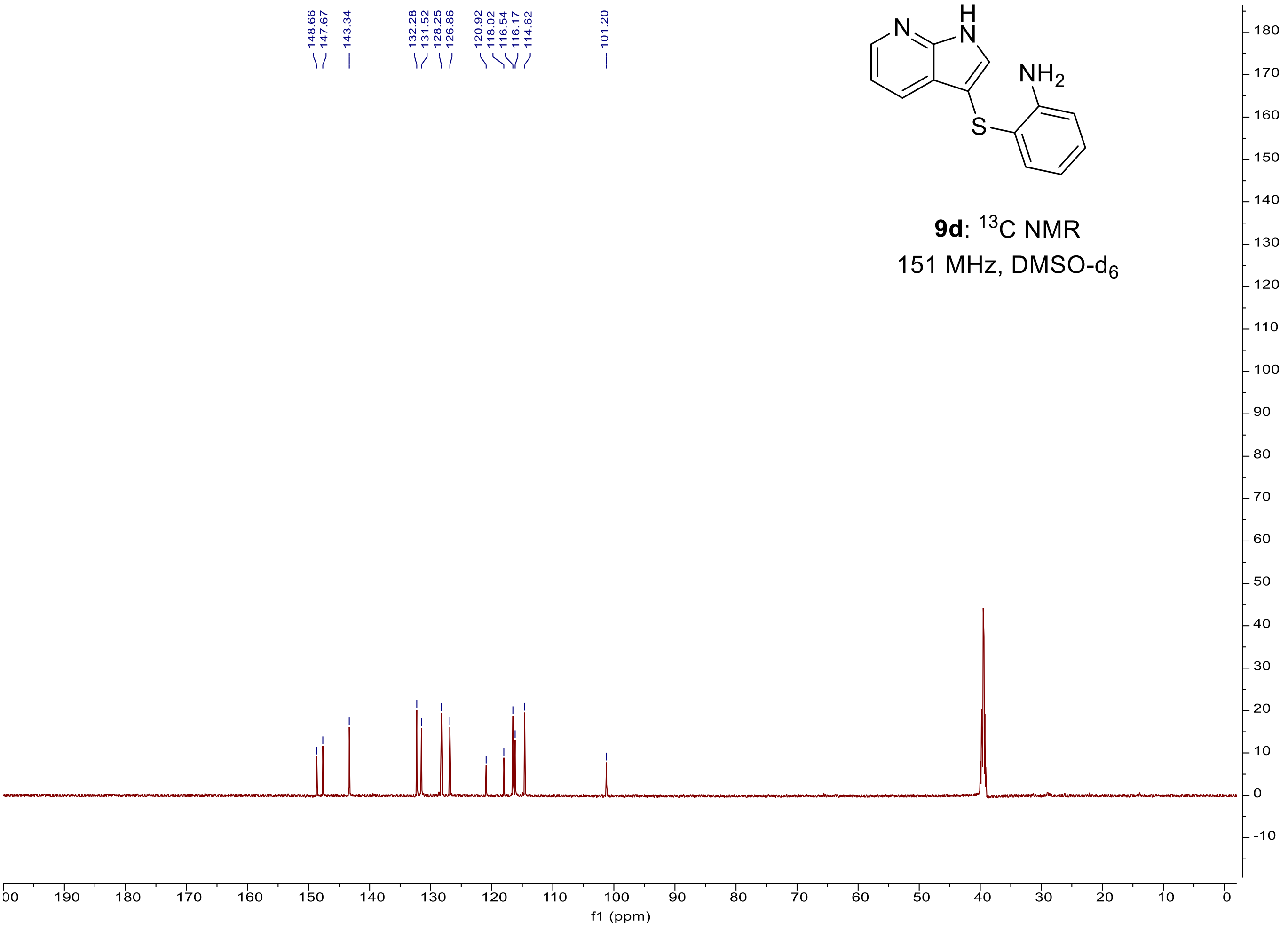
148.66
147.67
143.34
132.28
131.52
128.25
126.86
120.92
118.02
116.54
116.17
114.62
101.20
N
H
N
NH2
S
9d: 13C NMR
151 MHz, DMSO-d6
f1 (ppm)

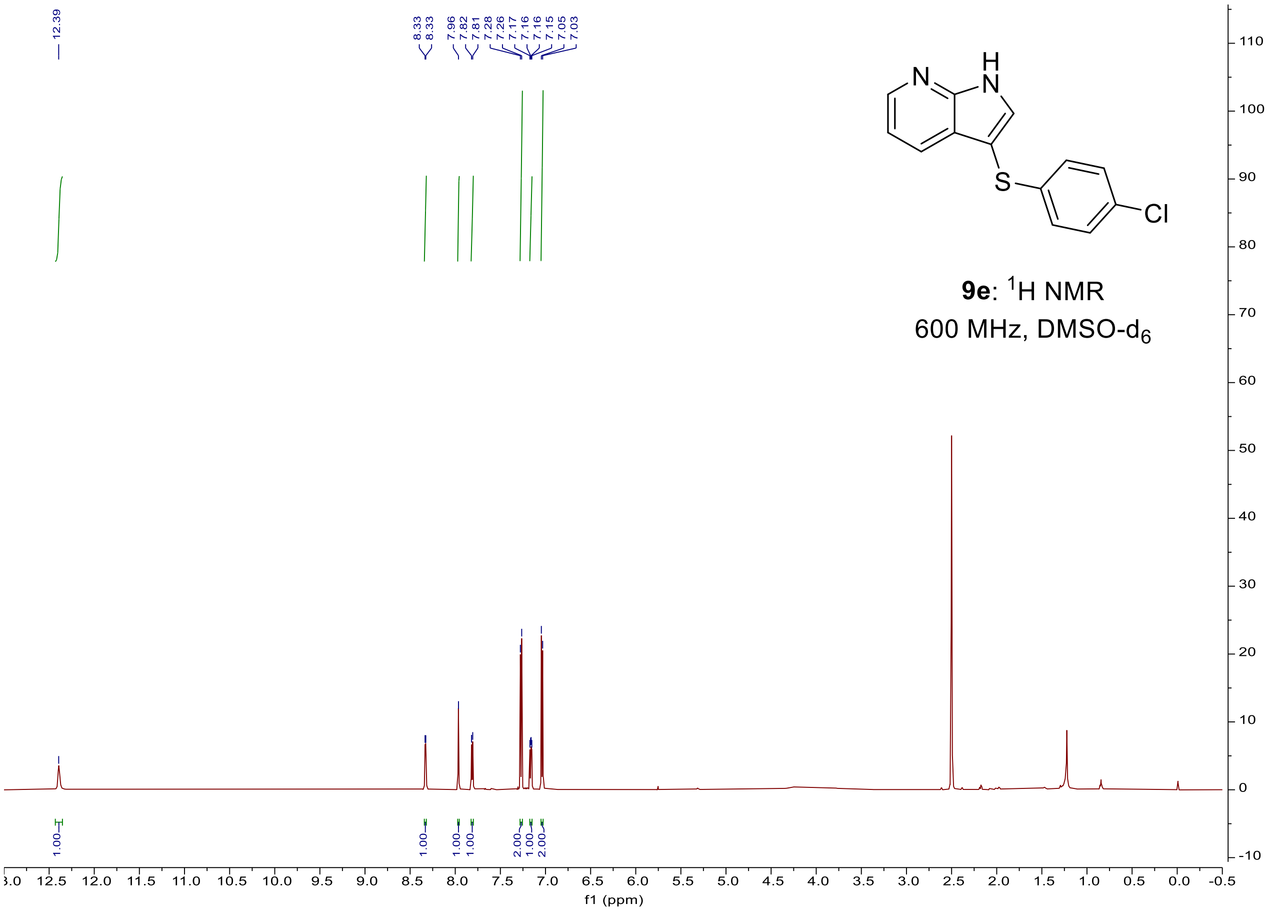
12.39
8.33
8.33
7.96
7.82
7.81
7.28
7.26
7.17
7.16
7.16
7.15
7.05
7.03
H
N
N
S
Cl
9e: 1H NMR
600 MHz, DMSO-d6
1.00
1.00
1.00
1.00
2.00
1.00
2.00
f1 (ppm)

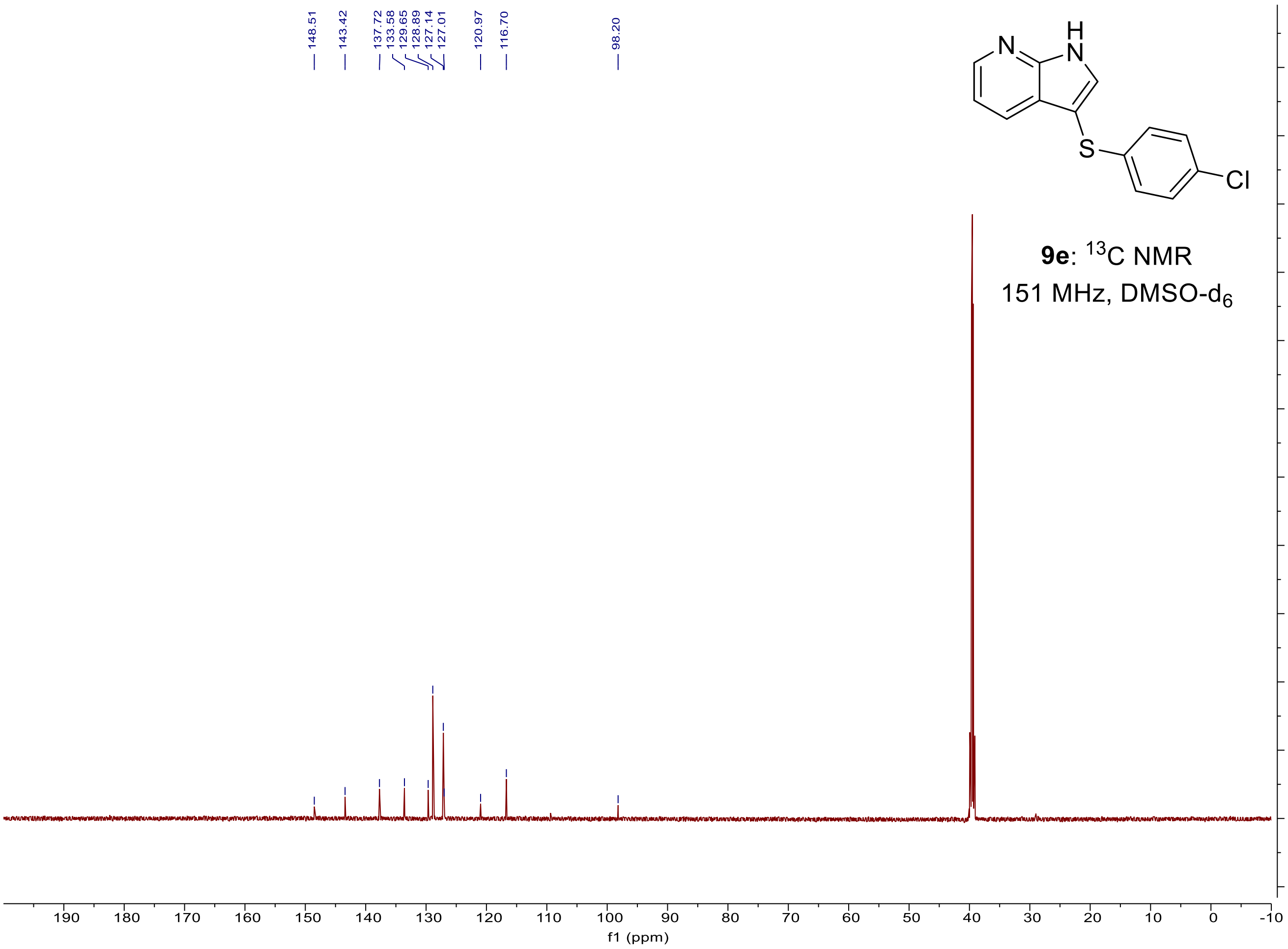
148.51
143.42
137.72
133.58
129.65
128.89
127.14
127.01
120.97
116.70
98.20
H
N
N
S
Cl
9e: 13C NMR
151 MHz, DMSO-d6
f1 (ppm)

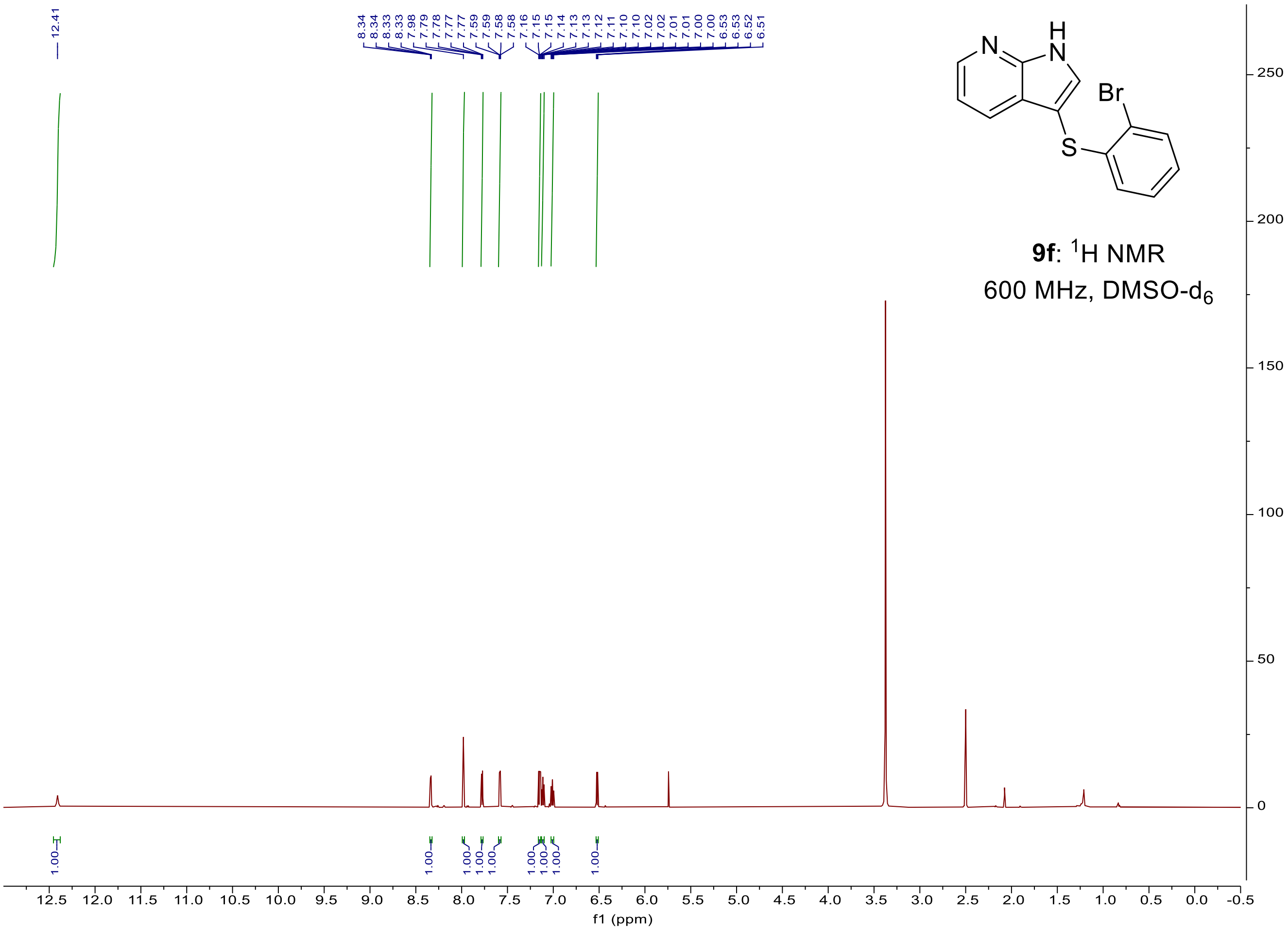
9f: 1H NMR
600 MHz, DMSO-d6
f1 (ppm)

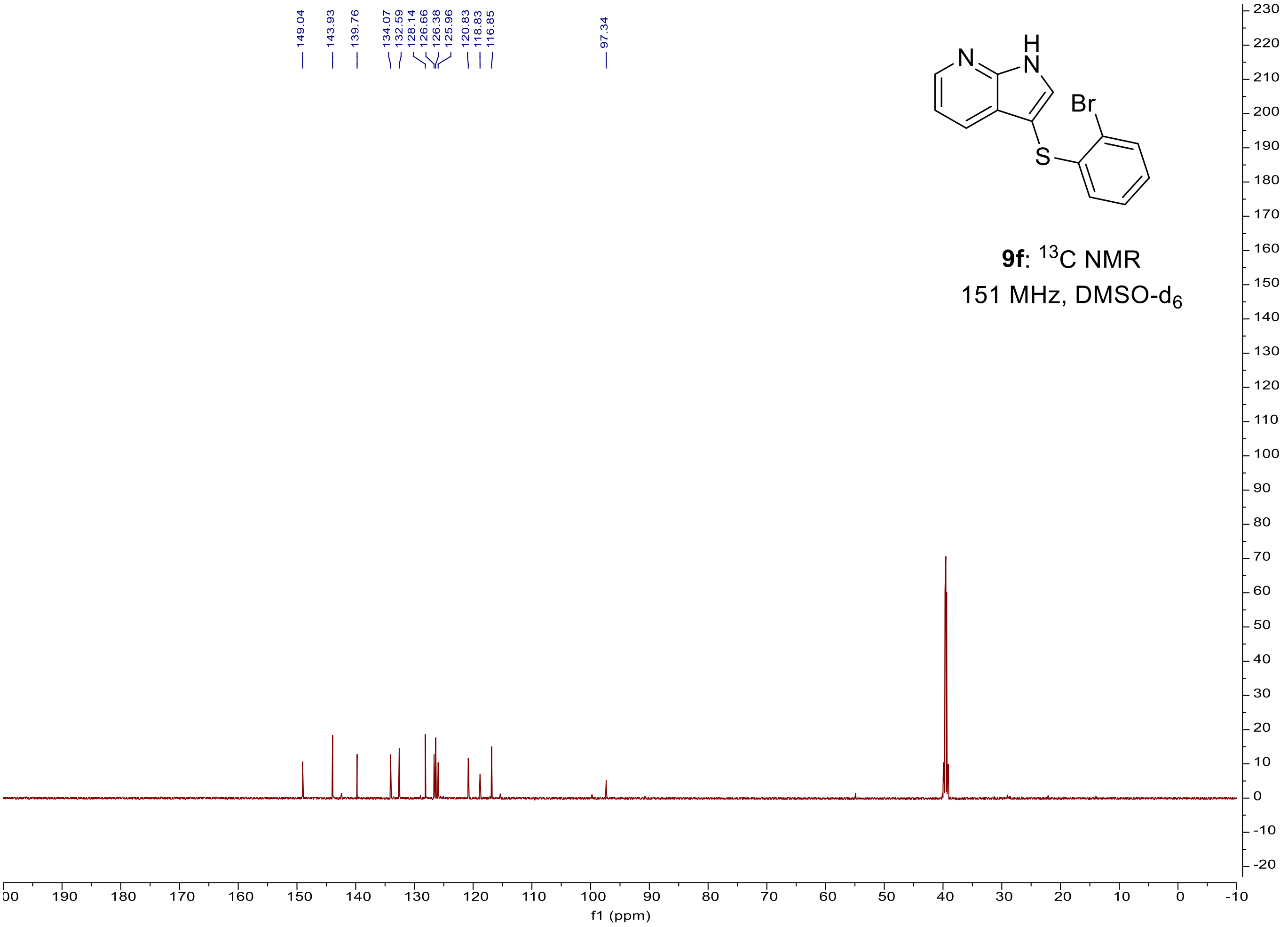
149.04
143.93
139.76
134.07
132.59
128.14
126.66
126.38
125.96
120.83
118.83
116.85
97.34
N
H
N
Br
S
9f: ^{13}C NMR
151 MHz, DMSO-d_6
f1 (ppm)

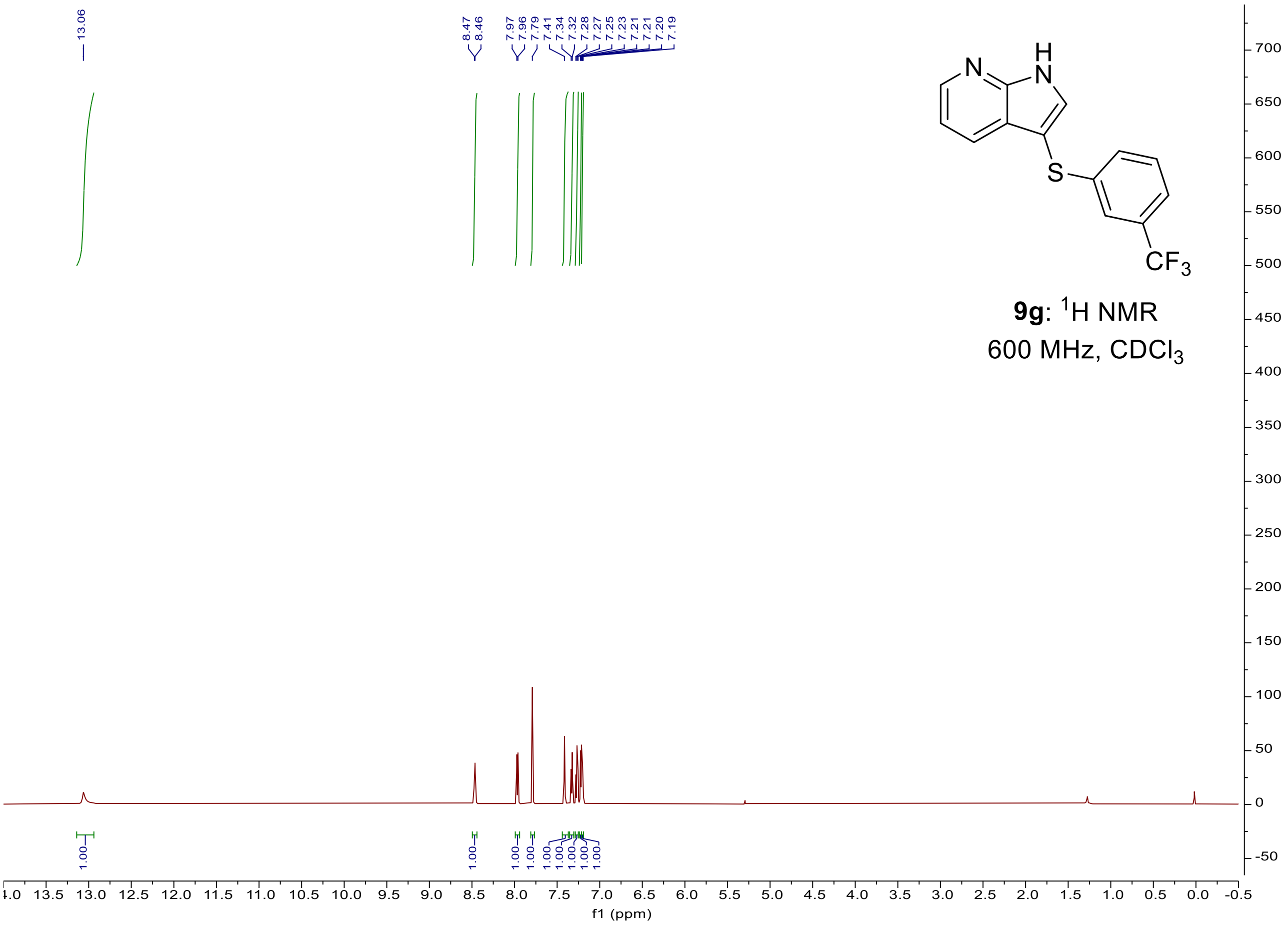
13.06
8.47
8.46
7.97
7.96
7.79
7.41
7.34
7.32
7.28
7.27
7.25
7.23
7.21
7.21
7.20
7.19
N
H
N
S
CF3
9g: 1H NMR
600 MHz, CDCl3
f1 (ppm)

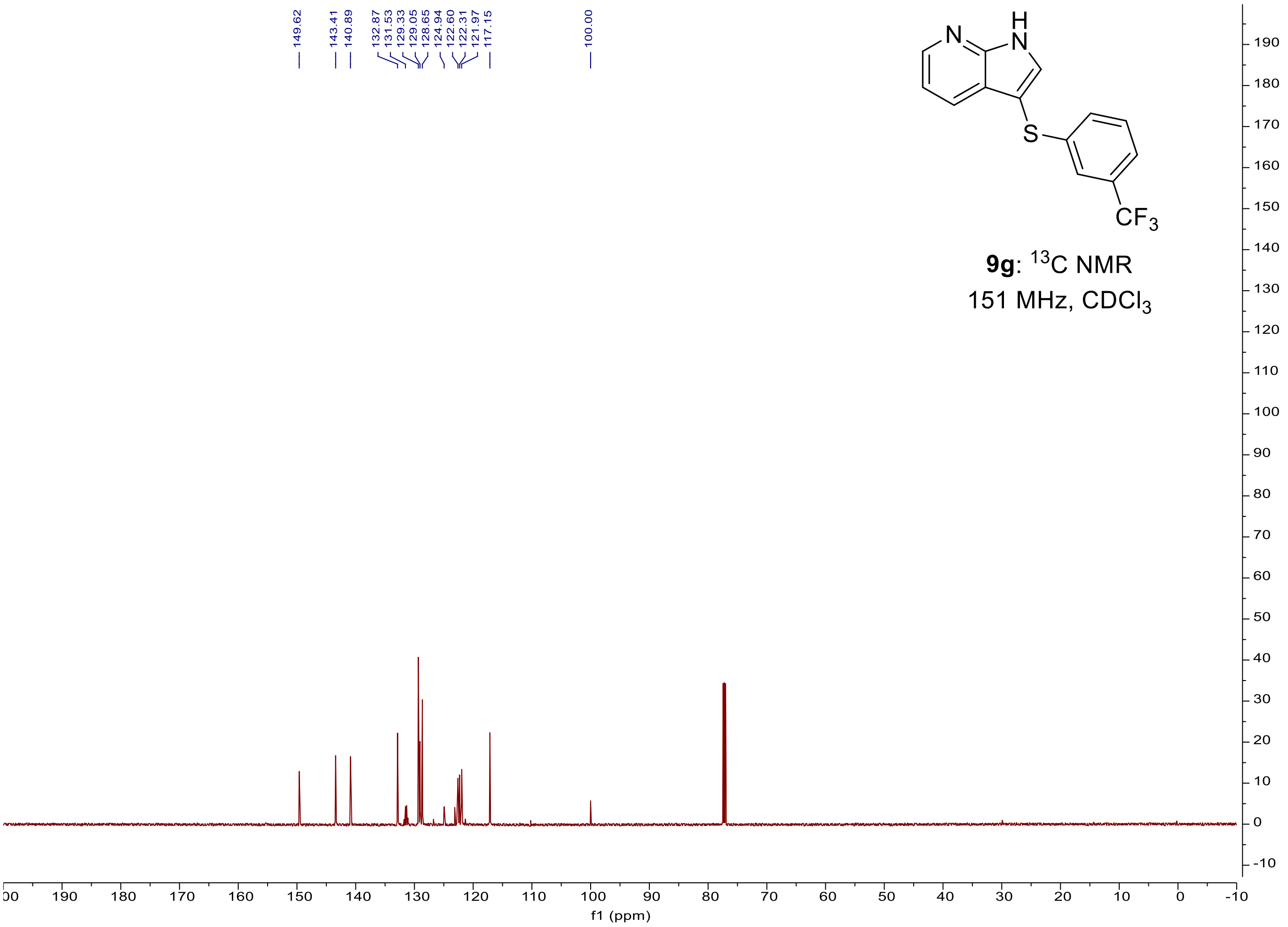
149.62
143.41
140.89
132.87
131.53
129.33
129.05
128.65
124.94
122.60
122.31
121.97
117.15
100.00
N
H
N
S
CF3
9g: 13C NMR
151 MHz, CDCl3
f1 (ppm)

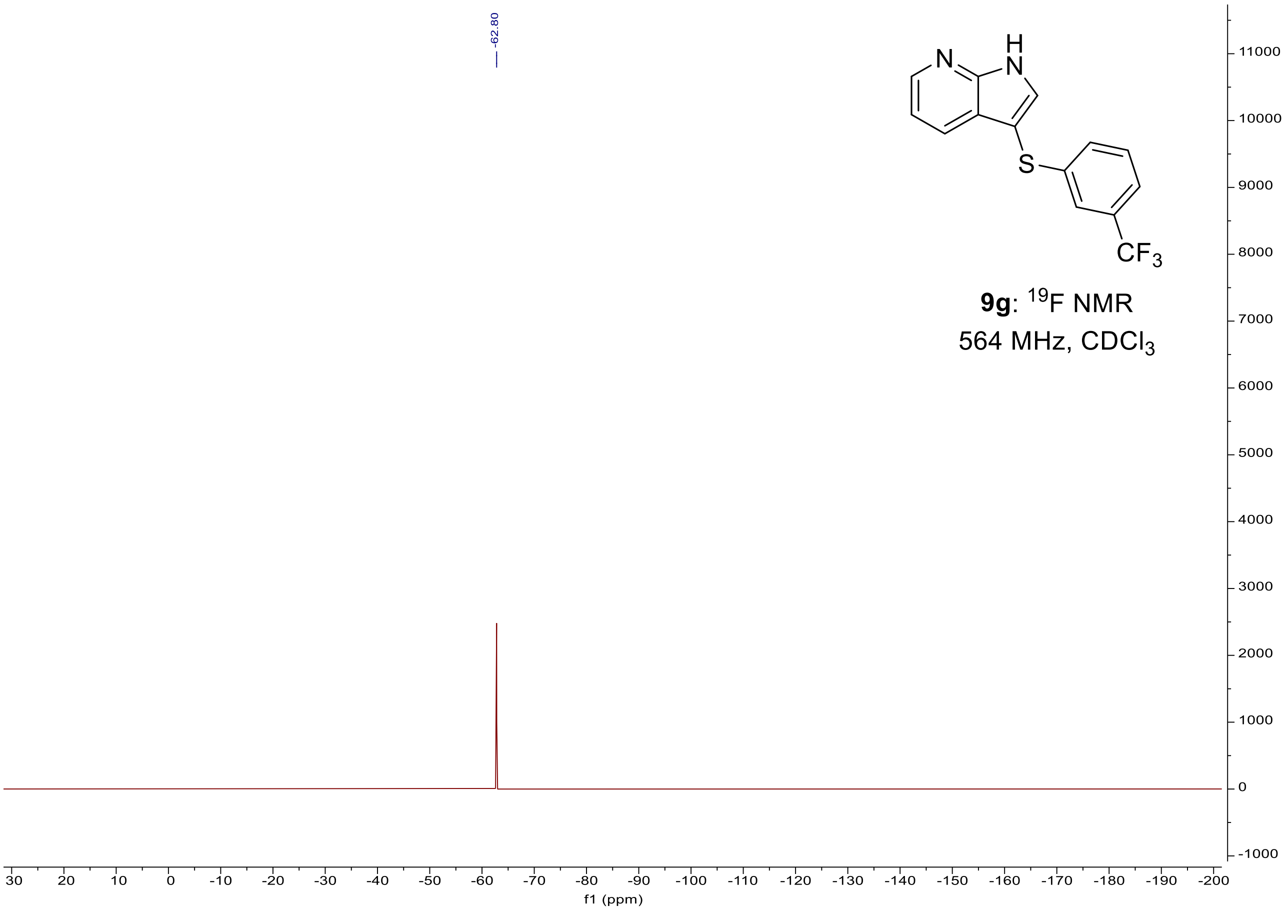
-62.80
9g: 19F NMR
564 MHz, CDCl3
f1 (ppm)
